# The Discovery of TNG456: A Highly Potent, Selective, Brain-Penetrant MTA-Cooperative PRMT5 Inhibitor for the Treatment of *MTAP*-Deleted Cancers

**DOI:** 10.1021/acs.jmedchem.6c00035

**Published:** 2026-05-18

**Authors:** Kevin M. Cottrell, Kimberly J. Briggs, Alice Tsai, Colin Liang, Patrick McCarren, Douglas A. Whittington, Minjie Zhang, Wenhai Zhang, Alan Huang, Jannik Andersen, John P. Maxwell

**Affiliations:** 506635 Tango Therapeutics, 201 Brookline Ave, Boston, Massachusetts 02215, United States

## Abstract

Homozygous deletion of the methylthioadenosine phosphorylase (*MTAP*) gene occurs in 10–15% of all human cancers and up to 50% of high-grade malignant gliomas, representing one of the largest opportunities for precision oncology. Loss of MTAP leads to the accumulation of 5′-methylthioadenosine (MTA), which sensitizes tumor cells to inhibition of protein arginine methyltransferase 5 (PRMT5). Herein we describe the discovery of **TNG456**, a potent and highly selective MTA-cooperative PRMT5 inhibitor that is brain penetrant in preclinical species and currently in Phase I/II clinical studies for the treatment of advanced or metastatic solid tumors with MTAP loss, with a focus on glioblastoma.

## Graphical abstract

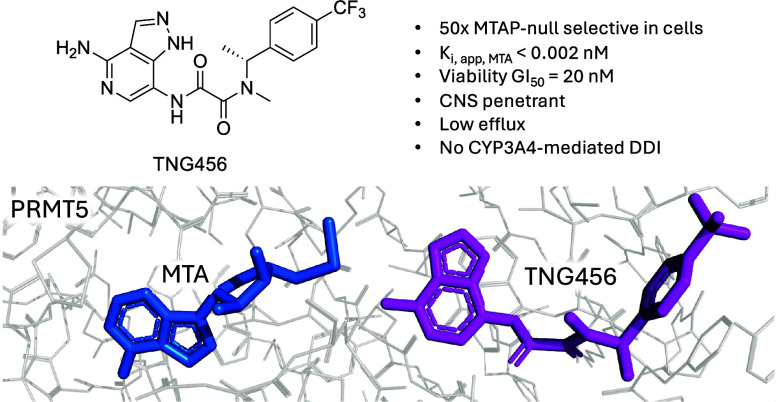

## Introduction

The methylthioadenosine phosphorylase (*MTAP*) gene is deleted in approximately 10–15% of human cancers due to its proximity to the tumor suppressor gene *CDKN2A.* ^1–3^ The *MTAP* gene encodes the MTAP enzyme which is involved in the methionine salvage pathway by catalyzing the phosphorolysis of 5′-methylthioadenosine (MTA). Loss of MTAP leads to accumulation of intracellular MTA, and elevated MTA selectively sensitizes MTAP-null tumor cells to inhibition of protein arginine methyltransferase 5 (PRMT5), a key regulator of gene expression, RNA splicing, and signal transduction.^4,5^ Since MTA acts as an *S*-adenosylmethionine (SAM)-competitive inhibitor of PRMT5,^6^ compounds such as vopimetostat (TNG462),^7^ AMG 193,^8^ BMS-986504 (MRTX1719),^9^ AZD3470,^10^ and TNG908,^11^ that bind PRMT5 cooperatively in complex with MTA (Figure 1), but not with SAM, can achieve selective inhibition in *MTAP*-deleted tumor cells while mitigating the dose-limiting toxicities in normal tissues that hindered first generation PRMT5 inhibitors. First generation clinical inhibitors were not MTA-cooperative and lacked *MTAP*-dependency as they inhibited PRMT5 by binding either competitively or cooperatively with SAM, thereby suppressing PRMT5 activity indiscriminately in both *MTAP*-deleted and *MTAP* intact normal cells.

**Figure 1:**
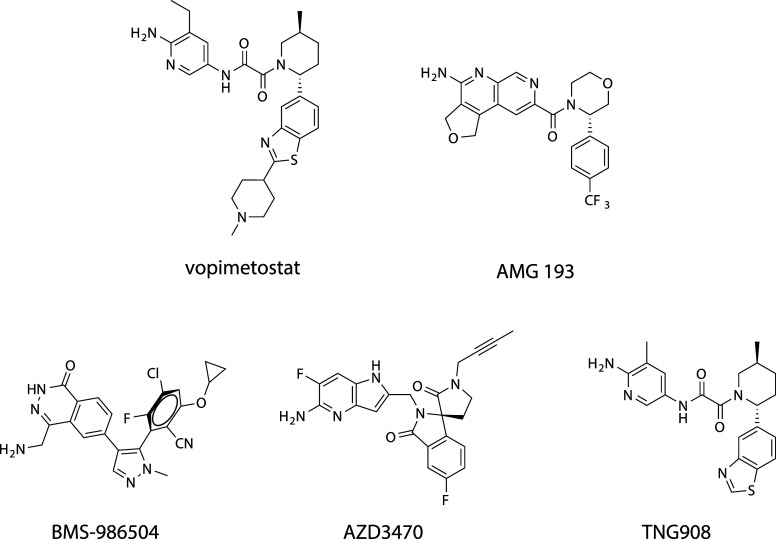
Chemical structures of MTA-cooperative PRMT5 inhibitors that are or have been in clinical development: vopimetostat (TNG462), AMG 193, BMS-986504 (previously MRTX1719), AZD3470, and TNG908.

TNG908 was the first development candidate from Tango Therapeutics targeting cancers with MTAP loss and it entered clinical studies in 2022. It has a PRMT5•MTA K_i,app_ = 80 pM, HAP1 MTAP-null cellular IC_50_ = 9 nM, cellular viability GI_50_ = 100 nM with 15-fold selectivity over MTAP-wild type (WT) cells.^11^ TNG908 is brain penetrant and was evaluated in clinical trials across multiple tumor types, including glioblastoma.^12^ As TNG908 was entering preclinical development, we continued lead optimization which ultimately led to the discovery of vopimetostat (TNG462).^7^ Vopimetostat is more potent and more selective for MTAP-null vs WT cells than TNG908, with a PRMT5•MTA K_i,app_ ≤ 300 fM, HAP1 MTAP-null cellular IC_50_ = 800 pM, viability GI_50_ = 4 nM, and average MTAP-null vs WT selectivity of 45-fold across multiple *MTAP*-isogenic pairs. Vopimetostat does not have properties typically associated with passive blood–brain barrier (BBB) penetration, i.e. permeability and efflux measured in an MDCK-II assay (MDCK-II A-B = 2.3 × 10^–6^ cm/s and MDR1 efflux ratio = 80). It entered clinical development in 2023.

During the preclinical development of TNG908 and vopimetostat, the depth and duration of PRMT5 target coverage required to drive maximal antitumor efficacy in humans remained undetermined. Based on preclinical pharmacology studies we expected the minimal trough concentrations required for efficacy to exceed the MTAP-null GI_50_ (which correlates with the MTAP-null PD SDMA IC_90_).^11^ However, the data suggested that achieving maximal single agent efficacy might require coverage beyond the MTAP-null GI_90_ at trough, roughly 3- to 5-fold greater than MTAP-null GI_50_. If such higher exposures were necessary, and encroached upon or exceeded PRMT5 WT GI_50_, tolerability could become dose-limiting, thereby eroding the therapeutic window between MTAP-null and WT contexts.

Additionally, for the treatment of CNS tumors such as glioblastoma, adequate brain exposure is essential. In the setting of brain penetration, the unbound brain-to-plasma ratio (K_p,uu_), which represents free brain vs free plasma concentrations,^13^ becomes a key parameter to consider in the context of efficacy relative to systemic exposure. A molecule with K_p,uu_ < 1 would require increased exposure in the periphery to achieve target exposures in the brain, thereby decreasing the maximal therapeutic index theoretically afforded by the MTAP-null vs WT potency differential.

Given the possibility that target coverage exceeding the HAP1 MTAP null GI_90_ might be required, as outlined above, and the risk that the K_p,uu_ of TNG908 in humans could be lower than that observed preclinically (predicted TNG908 K_p,uu_ = 0.9),^11^ it became apparent that the measured 15-fold *in vitro* selectivity of TNG908 in MTAP-null vs WT cells could be eroded in the clinical setting by one or both these factors. Consequently, the CNS concentration required for robust single-agent antitumor efficacy in tumors might not be achieved due to insufficient therapeutic index.

Considering this, we set out to identify a next generation brain penetrant molecule with potency, properties, and pharmacokinetics amenable to achieving exposure in the brain greater than MTAP-null GI_90_ at trough, yet remain below the WT GI_50_ in the periphery, thereby reducing toxicities driven by inhibition of PRMT5 in normal, MTAP proficient cells. To this end, the target candidate profile was defined as follows: HAP1 MTAP-null GI_50_ < 50 nM, selectivity vs WT > 40-fold, MDCK-II permeability > 10 × 10^–6^ cm/s (A-B), MDR1 efflux ratio < 3, and K_p,uu_ in cynomolgus monkey > 0.5. In addition, elimination of cytochrome P450 3A4 (CYP3A4)-related drug–drug interaction (DDI) potential was prioritized, as TNG908 had shown evidence *in vitro* for CYP3A4 time-dependent inhibition (TDI) (*vide infra*).

## Medicinal Chemistry

The aminopyrazolopyridine subseries that we had explored during the vopimetostat discovery effort provided a significant increase in potency relative to the analogous aminopyridine series (Figure 2), while maintaining excellent selectivity in most cases,^7^ so we decided to investigate additional structure activity relationships within this subseries. This work led to the discovery of compounds with a differentiated property profile.

**Figure 2:**
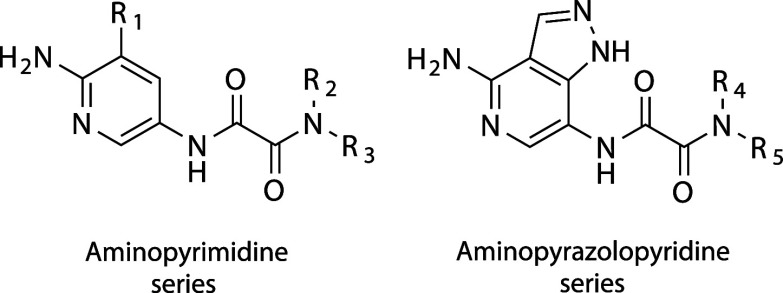
Oxamide subseries: Aminopyridines and aminopyrazolopyridines.

Despite the excellent potency and selectivity that had been demonstrated with the aminopyrazolopyridine series during the vopimetostat discovery campaign, the permeability and efflux profiles did not meet the program goals for a CNS penetrant molecule. For example, Compound **2** (Table 2) had low permeability (MDCK-II A-B = 2 × 10^–6^ cm/s) and high MDR1 efflux (ratio = 39), indicating suboptimal characteristics for brain exposure. We therefore sought to improve these properties while maintaining the favorable potency and selectivity profile of the series.

The physicochemical properties of a compound can greatly impact permeability and efflux, and consequently its potential to reach the CNS.^14–16^ Several molecular descriptors have been discussed in the literature as potentially relevant to brain penetrance, including hydrogen bond donors (HBD), topological polar surface area (TPSA), lipophilicity (cLogP), distribution coefficient (cLogD), compound ionization (pK_a_), and molecular weight (MW). Empirical analyses across CNS-penetrant small molecules have led to proposals of approximate optimal ranges: HBD ≤ 2, TPSA ≥ 40 Å^2^ and ≤ 90, cLogP ≤ 3, cLogD ≤ 2, pK_a_ ≤ 8, MW ≤ 360).^17–19^ While these serve as useful guidelines, the preferred values are not absolute and can vary among different chemical series, and the overall balance of these physicochemical properties is generally more predictive of brain exposure than any one single parameter. Achieving properties within these ranges while simultaneously maintaining potency, selectivity, and other favorable drug-like attributes presents significant challenges, as optimization of one parameter often negatively impacts others. We investigated whether compounds meeting our target profile could be identified within our aminopyrazolopyridine series.

Holding the core aminopyrazolopyridine-oxamide scaffold (Figure 2) constant puts two of the most important parameters affecting BBB penetration, HBD count^20^ and TPSA,^16,21^ outside the preferred range (HBD = 3 and TPSA = 117 Å^2^). Additionally, internal data collected during the vopimetostat discovery phase revealed that TPSA was highly correlated to MDR1 efflux ratio in the oxamide series (Figure 3). Specifically, 43% of compounds (26 of 60) with TPSA < 100 Å^2^ exhibited an MDR1 efflux ratio < 3, compared with 15% of compounds (26 of 169) with TPSA 100–120 Å^2^, and 0% (0 of 40) with TPSA > 120 Å^2^. Since we had set an MDR1 efflux ratio target of < 3 for a clinical candidate selection, these data suggested that utilizing a core with a starting TPSA of 117 Å^2^ could make achieving brain penetration within this series inherently challenging.

**Figure 3:**
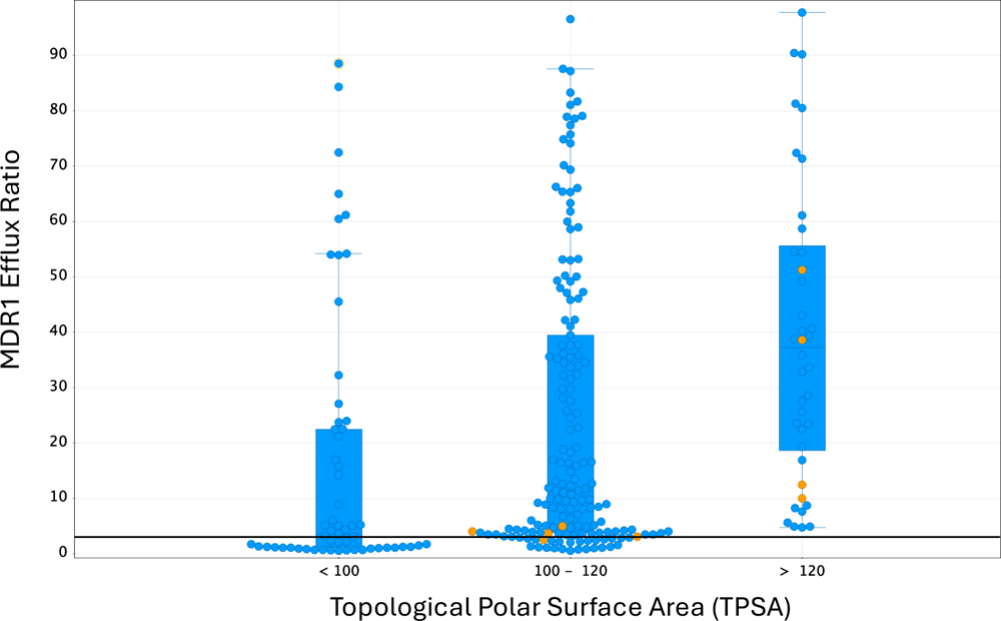
MDR1 efflux ratio vs topological polar surface area (TPSA) for oxamide series up to the discovery of vopimetostat (*N* = 269). The solid black line represents MDR1 efflux ratio = 3. Orange dots represent aminopyrazolopyridine analogs. The MDR1 efflux was ≤ 3 for 26/60 compounds with TPSA < 100 Å^2^, 26/169 compounds with TPSA between 100 and 120 Å^2^, and 0/40 compounds with TPSA > 120 Å^2^.

However, two intramolecular hydrogen bonds (IMHBs) ameliorate the effect of two of the three HBD groups; the NH in the pyrazolopyridine ring can form a 7-membered ring IMHB with the adjacent oxamide carbonyl and the NH of the oxamide can form a 5-membered ring IMHB with the other carbonyl thereby reducing the potential negative impact of hydrogen bond donors to the overall properties of the molecule.^22^ Density functional theory (DFT) calculations performed on a model aminopyrazolopyridine oxamide (truncated to the dimethyl variant for simplicity) confirm that the IMHBs are present in the energetically preferred conformation (Figure 4). With the polarity of these two HBDs effectively masked through IMHB formation we prioritized the design of analogs that introduced no additional heteroatoms beyond the aminopyrazolopyridine oxamide core, limiting the TPSA to a maximum of 117 Å^2^, to increase the potential to achieve the desired profile.

**Figure 4:**
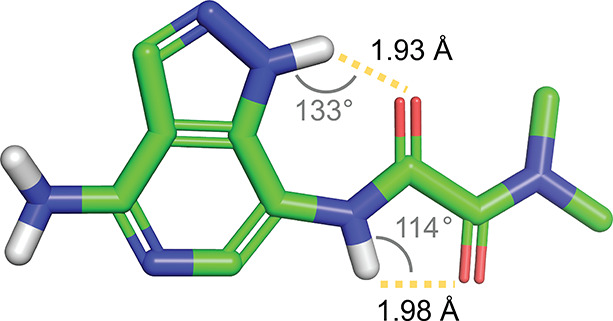
Lowest energy conformation of a model amino-pyrazolopyridineoxamide using DFT has an intramolecular hydrogen bond that is preferred by 5.5 kcal/mol after optimization in the gas phase (B3LYP-D3/6-311G**) and 2.6 kcal/mol after accounting for aqueous solvation using single-point CPCM implicit solvation model calculations with the same level of theory (CPCM/B3LYP-D3/6-311G**//B3LYP-D3/6-311G**) (see ).

Representative early analogs **1** and **2** in the pyrazolopyridine series both exhibited clogP and logD values near the desired range (calculated logP = 4.8 and 2.7; measured logD = 1.3 and 1.9 for **1** and **2**, respectively) (Table 2). Anticipating the need to reoptimize the piperidinyl amide portion of the molecule, we were encouraged that these early analogs already were close to our target lipophilicity range.

Previously, we reported that the aminopyrazolopyridine analogs containing a basic amine had significantly reduced MTAP-null to WT selectivity relative to the analogous aminopyridines, despite increased potency by engaging Glu320 in a salt bridge.^7^ For example, the matched molecular pair vopimetostat and **1** had similar MTAP-null viability potencies (HAP1 MTAP-null GI_50_ = 4 and 2 nM, respectively), yet the aminopyrazolopyridine analog (**1**) exhibited markedly lower selectivity (30-fold vs 6-fold, for vopimetostat and **1**, respectively). In contrast, without the basic amine, both potency and selectivity of the aminopyrazolopyridines was generally excellent, as demonstrated by the matched molecular pair **1** and **2** (HAP1 MTAP-null GI_50_ = 2 and 6 nM; HAP1 MTAP-null selectivity vs WT = 6-fold vs 35-fold, respectively). Removal of the highly basic piperidine in the series reduced pK_a_ from ∼ 10 to ∼ 6, bringing it into the desired range for CNS penetration. Importantly, this adjustment did not compromise potency in this subseries relative to analogs containing the basic amine, suggesting that designing molecules with pK_a_ more favorable for brain exposure would be possible.

The final goal of the program was to minimize drug–drug interaction (DDI) potential. TNG908 had an IC_50_ shift in both CYP3A4 time-dependent inhibition (TDI) and k_inact_/K_I_ assays (Table 1). In contrast, the majority of aminopyrazolopyridines analogs evaluated at the start of this effort did not shift IC_50_ in the CYP3A4 TDI assay (data not shown), indicating a substantially lower risk for CYP3A4-mediated TDI within this subseries. These findings increased the overall appeal of the series and justified its continued exploration.

**Table 1:** CYP3A4 Time-Dependent Inhibition by TNG908: Summary of IC_50_ Shift, K_I_, and k_inact_ Data

CYP3A4 IC_50_ shift assay			
Substrate	IC_50_ (-NADPH), μM	IC_50_ (+NADPH), μM	Ratio
Midazolam	7.7	1.07	7.2
Testosterone	6.3	0.83	7.6

CYP3A4 k_inact_/K_I_ assay			
Substrate	K_I,_ μM	k_inact_, min^–1^	k_inact_/K_I_, min^–1^ μM^–1^
Midazolam	11.7	0.291	0.025
Testosterone	9.6	0.255	0.027

## Results

Since lower molecular weight is more favorable for brain penetration, a series of compounds incorporating incremental reduction in the molecular weight of the substituent at the piperidine 2-position (Table 2) was prepared. Removing the thiazole portion of **2** gave the phenyl analog **3** which maintained comparable potency (HAP1 MTAP-null GI_50_ = 6 and 7 nM, respectively). In contrast, the matched pair change from benzothiazole to phenyl in the aminopyridine series led to ∼ 5-fold loss in potency.^11^ The modification maintained good selectivity (HAP1 MTAP-null selectivity vs WT = 35- and 45-fold, respectively), and significantly improved permeability and efflux properties (MDCK A-B = 2 and 12 × 10^–6^ cm/s, MDR1 efflux ratio = 39 and 2, respectively). The observed improvement in passive permeability and reduction in efflux upon removal of the heteroatoms supported our hypothesis that highly permeable, low efflux compounds could be achieved with 3 HBD and PSA = 117 Å^2^. Collectively, these results increased confidence in the developability of the series. Compound **3**, which met the potency, selectivity, permeability, and efflux goals, was advanced for further ADME and DMPK evaluation.

**Table 2:**
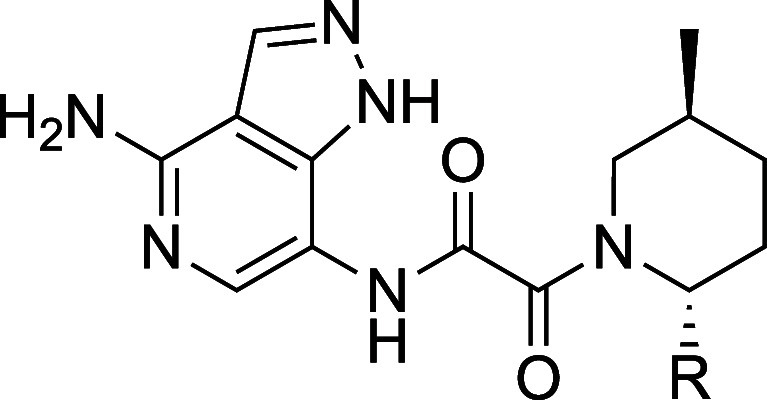

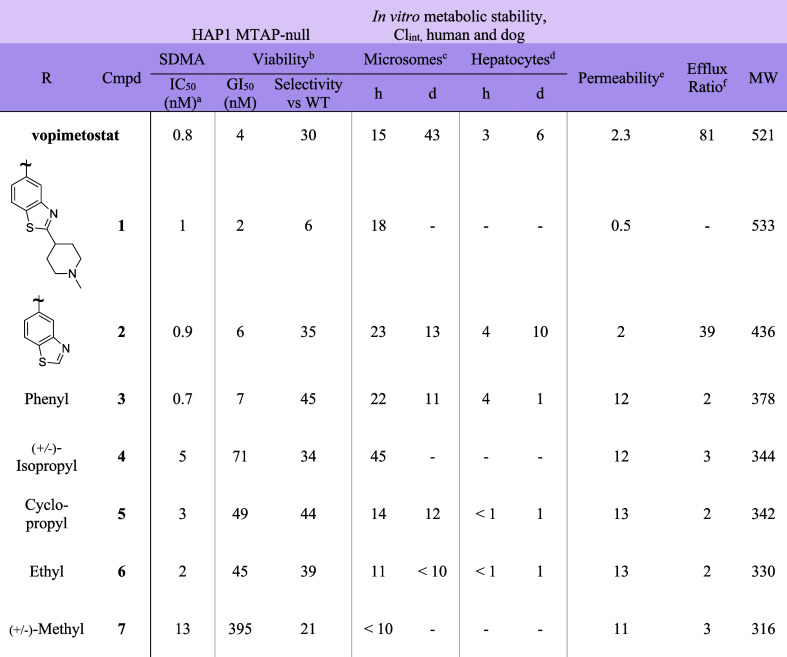
Cellular potency and selectivity, *In Vitro* Metabolic Stability, MDCK Permeability, MDR1 Efflux, and Molecular Weight Data for Vopimetostat and Compounds **1**–**7**

a Inhibition of PRMT5 determined by an SDMA in-cell western assay in the HAP1 *MTAP*-isogenic cell line pair following 24 h compound treatment.

b Viability growth inhibition assessed after 7 days using a CellTiter-Glo luminescence-based assay in HAP1 MTAP-null and HAP1 MTAP WT cells.

c Human and dog liver microsomes, CL_int_, μL min^–1^ mg^–1^.

d Human and dog hepatocytes, CL_int_, μL min^–1^ (10^6^ cells)^−1^.

e MDCKII-WT cells, A-B (10^–6^ cm/s).

f MDCKII-MDR1 cells (A-B)/(B/A).

The *in vitro* stability of **3** in liver microsomes was moderate to high in humans and dogs (hCL_int, mic_ = 22 μL/min/mg; dCL_int, mic_ = 11 μL/min/mg). As previously reported,^11^ the use of rodents in PK evaluation of oxamides was avoided due to instability in rodent plasma, thus *in vivo* assessments were conducted in dogs. A single intravenous dose (1 mpk IV) of **3** in beagle dogs gave a CL = 13 mL/min/kg and oral administration (3 mpk PO) demonstrated 100% bioavailability (F = 100%). Despite these favorable PK properties, **3** was deprioritized due to brain penetration observed in the cynomolgus cerebrospinal fluid (CSF) model lower than the program target (K_p,uu_ < 0.3) and a predicted short human half-life, which suggested a three-times-daily dosing regimen to maintain coverage above the efficacious target (data not shown) would likely be required.

Further reducing molecular weight, we examined (*±*)*-*isopropyl (**4**), cyclopropyl (**5**), ethyl (**6**), and (*±*)-methyl (**7**) in place of phenyl. Compounds **4**, **5**, and **6** had a 6- to 10-fold decrease in cellular viability potency relative to **3** (HAP1 MTAP-null GI_50_ = 71, 49, and 45 nM, respectively) but retained good selectivity (vs WT = 34-, 44-, and 39-fold, respectively) while **7** lost > 50-fold in potency. All analogs had favorable permeability and efflux profiles (MDCK A-B (× 10^–6^ cm/s) = 12, 13, 13, and 11 respectively and MDR1 efflux ratio = 3, 2, 2, and 3, respectively).

Compounds **4**, **5**, and **6** exhibited properties approaching the desired profile and were entered into ADME testing. **4** had poor human microsomal stability (hCL_int, mic_ = 45 μL/min/mg), whereas **5** and **6** had good stability in both human and dog liver microsomes (hCL_int, mic_ = 14 and 11 μL/min/mg and dCL_int, mic_ = 12 and <10 μL/min/mg, respectively) and hepatocytes (hCL_int, hep_ < 1 μL/min/10^6^ cells and dCL_int, hep_ = 1 μL/min/10^6^ cells for both). And although neither compound exhibited reversible or time-dependent inhibition of CYP3A4, *in vivo* single dose (1 mpk IV) studies in beagle dogs revealed higher than expected clearance values (CL = 41 and 31 mL/min/kg, for **5** and **6**, respectively), despite their favorable *in vitro* stability. Due to this lack of *in vitro–in vivo* correlation we continued the exploration to identify molecules with improved metabolic behavior, as we were encouraged that low molecular weight analogs such as **6** (MW = 330), could meet program goals for potency, selectivity, permeability, and efflux.

Earlier in the development of the oxamide series, acyclic analogs were explored in addition to the piperazine, morpholine, and piperidine analogs that ultimately led to the clinical candidates TNG908 and vopimetostat. Although the acyclic analogs had promising potency, their exploration was limited due to rapid advancement of the piperidine series (data not reported). The emergence of the more potent aminopyrazolopyridine scaffold provided an opportunity to revisit these acyclic designs.

Previously disclosed acyclic aminopyridine and carboxamidopyridine analogs (**8** and **9**, Table 3 **)** had potencies within a few fold of their corresponding *trans*-5-methyl-2-phenyl piperidine analogs (**10** and **11**) (HAP1 MTAP-null GI_50_ = 1800, 1200, 700, and 400 nM for **8**, **9**, **10**, and **11**, respectively). A cocrystal structure of **8** in the PRMT5•MTA active site (Figure 5) reveals that one of the phenyl groups overlays the phenyl at the piperidinyl 2-position of **10** and the other overlays the 4- and 5- positions of the piperidine. While not obvious in the 2D representations, this overlap suggested the dibenzylamide could serve as an interesting 1,4-*trans*-substituted piperidine bioisostere offering a starting point for design of acyclic analogs with additional flexibility for physicochemical optimization.

**Figure 5:**
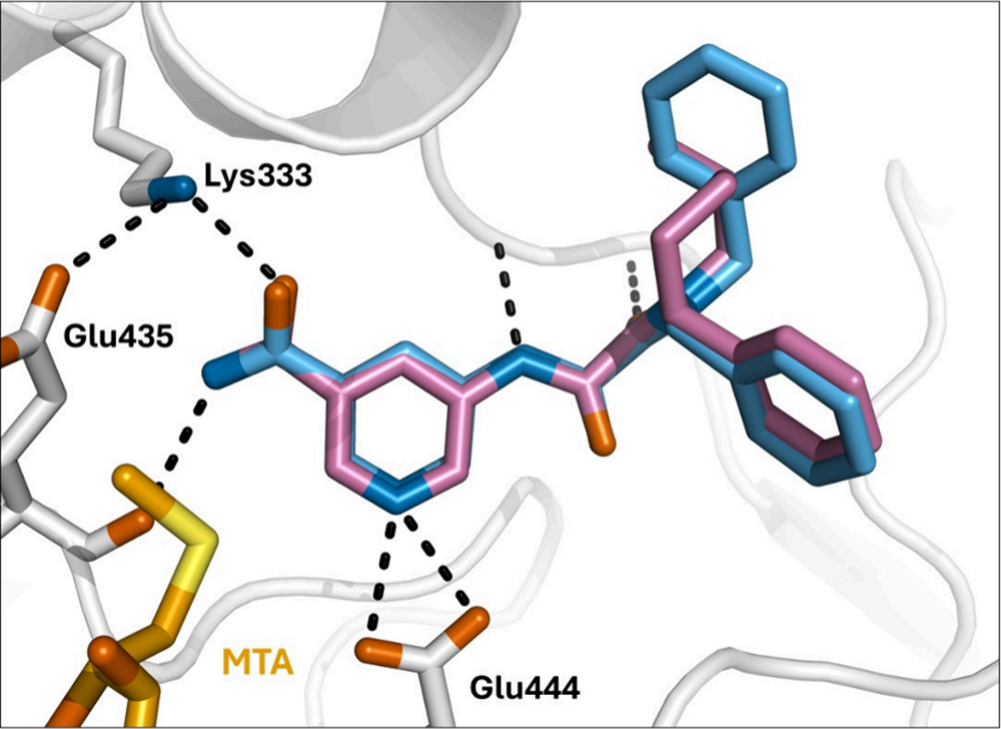
Overlay of **8** (blue, PDB 9ZL2) and **10 (**violet, PDB 8VEW) from their cocrystal structures with PRMT5:MEP50•MTA showing the spatial overlap of the dibenzyl substituents of **8** with the cyclic piperidine moiety of **10**.

**Table 3:**
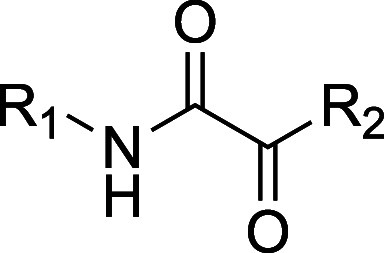

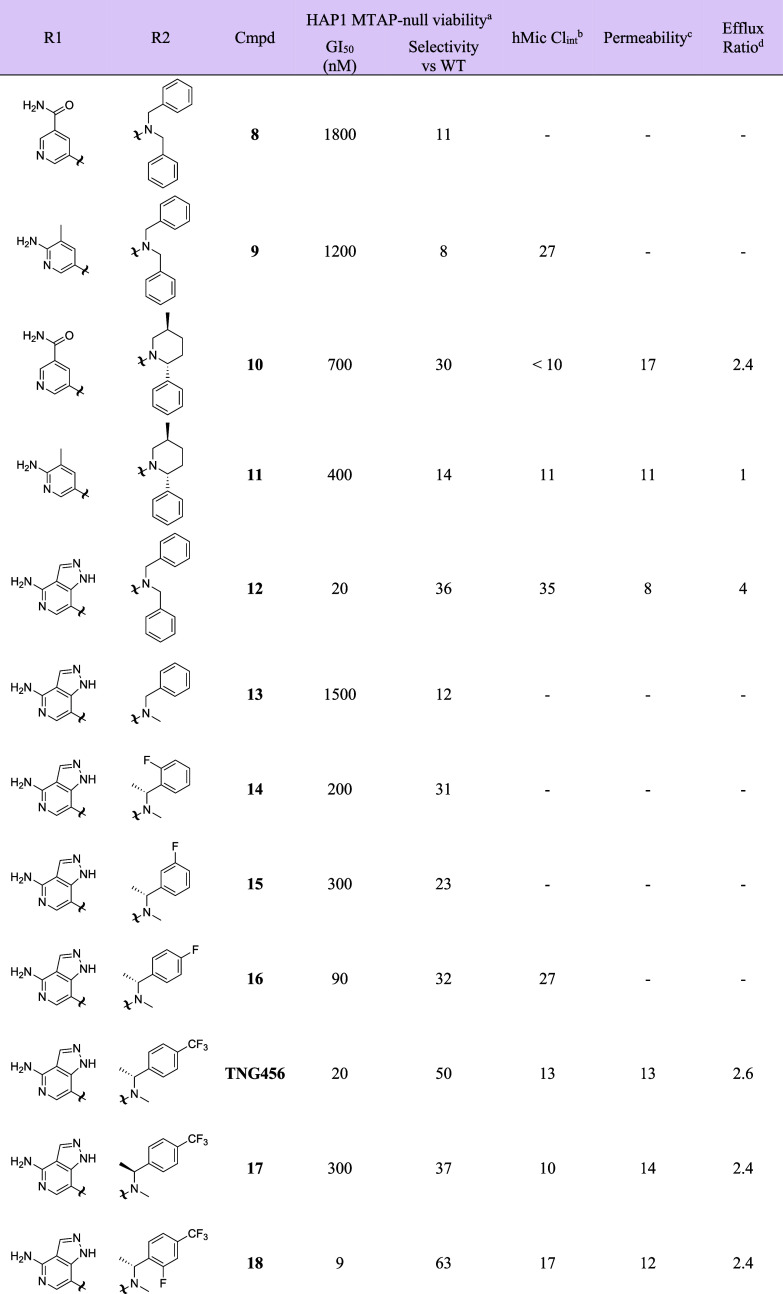

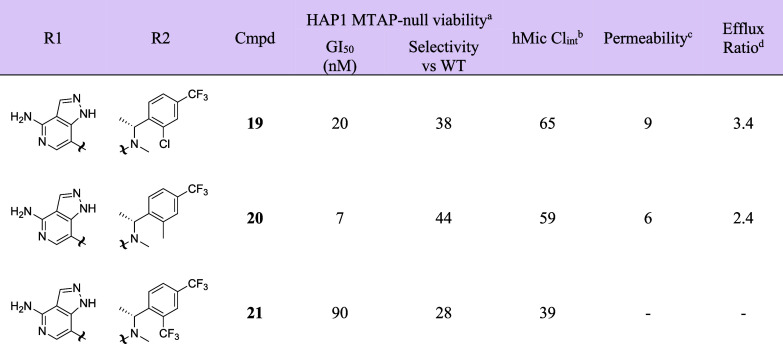
Cellular Viability, Microsomal, Permeability, and Efflux Data for Compounds **8–23** and **TNG456**

a Viability growth inhibition assessed after 7 days using a CellTiter-Glo luminescence-based assay in HAP1 MTAP-null and HAP1 MTAP WT cells.

b Human liver microsomes, CL_int_, μL min^–1^ mg^–1^.

c MDCKII-WT cells, A-B (10^–6^ cm/s).

d MDCKII-MDR1 cells (A-B)/(B/A).

Next we prepared the analogous aminopyrazolopyridine analog (**12**) and it had strong activity (HAP1 MTAP-null GI_50_ = 20 nM) and favorable selectivity vs WT (36-fold). However, metabolic stability was poor (hCL_int, mic_ = 35 μL/min/mg) and permeability and efflux were only moderate (MDCK A-B = 8 × 10^–6^ cm/s, MDR1 efflux ratio = 4). To further optimize the balance of potency, stability, and physicochemical properties, we explored modifications to reduce MW while avoiding additional polarity.

Removal of one of the phenyl rings (**13**) led to a significant loss of potency (HAP1 MTAP-null GI_50_ = 1500 nM). This reduction in potency likely reflected decreased binding affinity due to both increased flexibility and reduced hydrophobic interactions than previous analogs. We hypothesized that increasing the barrier to rotation of the benzyl group would reduce the entropy difference on binding and improve affinity. A partially relaxed torsional DFT scan of the benzylic bond suggested the addition of a methyl group would effectively select for a single rotamer by penalizing one of the two rotamers seen in the unsubstituted benzyl compound. To test this hypothesis, the first set of compounds we prepared were the benzylic *R*-methyl analogs with *ortho*, *meta*, or *para*-fluorophenyl, **14**–**16**, since the *R*-enantiomer was preferred to the *S*-enantiomer when docked into the protein (see for details). Addition of this chiral benzylic methyl improved potency across all three analogs compared to **13** (HAP1 MTAP-null GI_50_ = 200, 300, and 90 nM for **14**, **15**, and **16**, respectively). Selectivity against MTAP WT cells remained high in these compounds, however metabolic stability needed further improvement (hCL_int, mic_ = 27 μL/min/mg for **16**).

Subsequent SAR exploration revealed that further MW reductions resulted in unacceptable potency loss (data not shown), so focus shifted to exploration of substitutions around the phenyl ring. Potential strategies to improve oxidative metabolic stability are to add polarity, to block potential sites of metabolism, and/or reduce electron density in aromatic rings with electron withdrawing groups.^23^ Since additional heteroatoms to increase polarity conflicted with our CNS penetration objectives, we focused instead on modulating the electronic properties of the aromatic ring. The results of the ‘fluorine walk’ had suggested that simply blocking aromatic sites might not be sufficient, prompting exploration of stronger electron withdrawing groups such as CF_3_ on the ring to improve metabolic stability. Since the *para-* position had the best tolerance to substitution in the fluorine walk, and crystal structures and SAR throughout the program suggested the *para-* position likely provided the most tolerance to larger substituents, we prepared the *para*-CF_3_ analog (**TNG456**).

This compound was initially prepared from racemic *N*-methyl-1-(4-(trifluoromethyl)­phenyl)­ethan-1-amine with separation of the enantiomers by chiral HPLC (**TNG456** and **17**). **TNG456** had excellent potency and selectivity (HAP1 MTAP-null GI_50_ = 20 nM, selectivity vs WT = 50-fold) and was 15-fold more potent than its *S*-enantiomer (**17**), one of several *S*-enantiomers that were prepared to support our earlier hypothesis that the *R*-enantiomer would be preferred. **TNG456** had promising metabolic stability, permeability, and efflux (hCL_int, mic_ = 13 μL/min/mg, MDCK A-B = 13 × 10^–6^ cm/s, MDR1 efflux ratio = 2.6), suggesting it had potential to cross the BBB. As for TDI, it had no signal in the CYP3A4 TDI IC_50_ shift assay, indicating a very low risk for CYP3A4 TDI and a reduced likelihood of drug–drug interactions in clinical settings.

Subsequent batches of **TNG456** were prepared from the known *R*-enantiomer of the benzylamine, confirming the stereochemical assignment of **TNG456**. A cocrystal structure of **TNG456** with PRMT5•MTA (Figure 6) and a small molecule crystal structure (See ) provided further proof. In the cocrystal structure, the benzylic group flips in the pocket relative to the aromatic substituents in the piperidine series and forms a π-stacking interaction with Phe580 and Tyr304 and the CF_3_ group fills a pocket created by the movement of the α helix formed by residues 296–301. The pocket is formed from the significant movements of Phe300, Tyr297, and Phe577 relative to their locations when 2-aryl-5-methylpiperidine analogs are bound. The bound conformation of TNG456 (Figure 6) differs from the IMHB conformation (Figure 4) by 5.9 kcal/mol (B3LYP-D3/6-311G**//B3LYP-D3/6-31G*), but the extensive hydrogen bond network achieved upon binding compensates for that energetic penalty.

**Figure 6:**
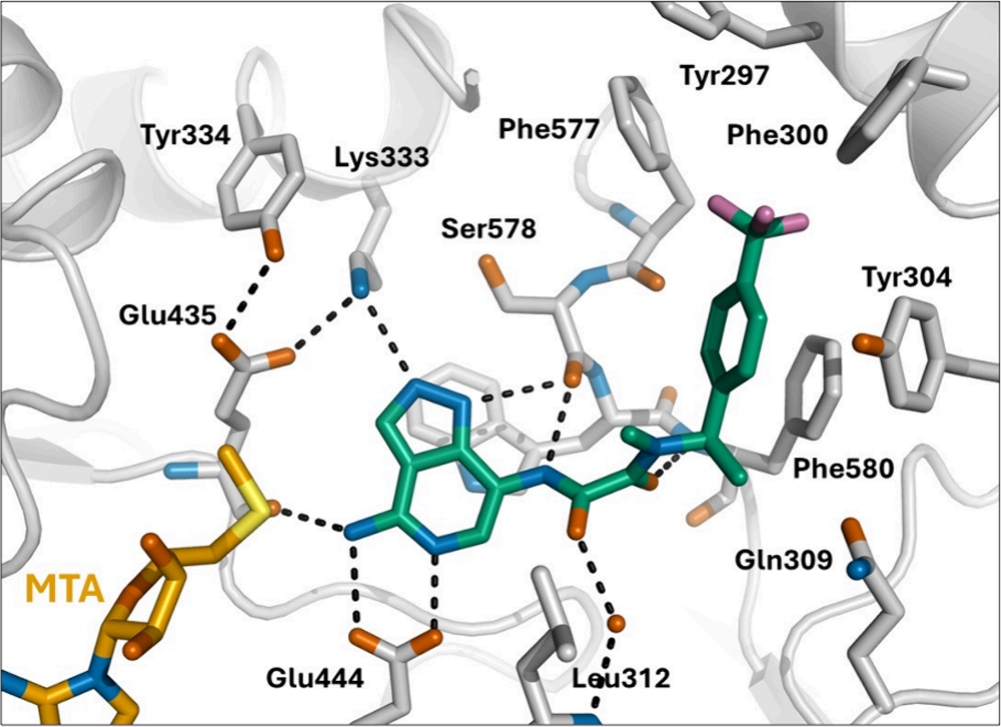
Cocrystal structure of **TNG456** (green) with PRMT5:MEP50•MTA (PDB 9ZL4). Dashed lines indicate hydrogen bonds.

Exploration of disubstituted analogs with *para*-CF_3_ and a variety of *ortho*- position substituents that intentionally avoided the inclusion of additional polarity (**18**–**21**) gave potent and selective compounds. For example, disubstituted analogs with F, Cl, CH_3_, and CF_3_ at the *ortho* position had HAP1 MTAP-null GI_50_ = 10, 20, 7, and 90 nM and selectivity vs WT = 63-, 38-, 44-, and 28-fold, respectively. However, all of them, except the fluorine analog (**18**) had reduced metabolic stability. Interestingly, the protein–ligand crystal structure of **18** showed evidence of a fluorine orthogonal multipolar interaction^24^ where the fluorine points toward the CO backbone of Trp579 (Figure 7).

**Figure 7:**
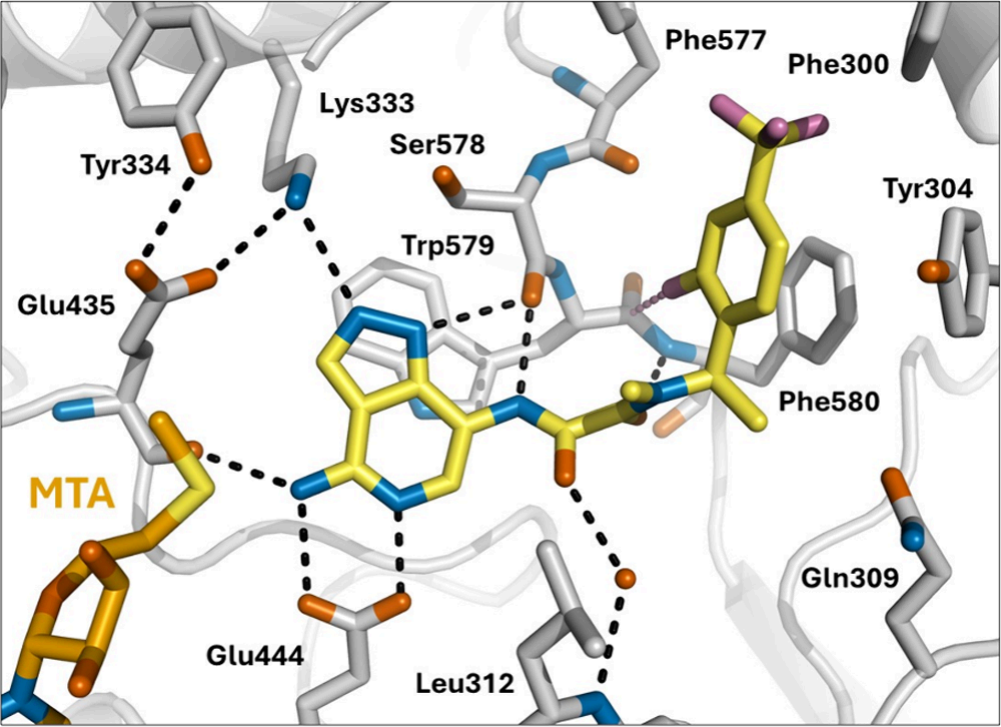
Cocrystal structure of **18** (yellow) with PRMT5:MEP50•MTA (PDB 9ZL3). Black dashed lines indicate hydrogen bonds, and the fluorine–carbonyl interaction is indicated in purple.

MDR1 assay results of the aminopyrazolopyridine analogs highlight that compounds with MDR1 efflux <3 could be identified within the series. The data confirm that introducing additional polar atoms typically increased the efflux ratio beyond the desired range, thereby compromising the potential BBB penetration. As shown in Figure 8, 58% (N = 31) compounds in the series with TPSA = 117 Å^2^ (corresponding to the unmodified core structure with no additional polar atoms) had efflux ratios < 3, whereas only 1% (N = 73) with TPSA > 117 Å^2^ maintained an efflux ratio within the desired range (Figure 8).

**Figure 8:**
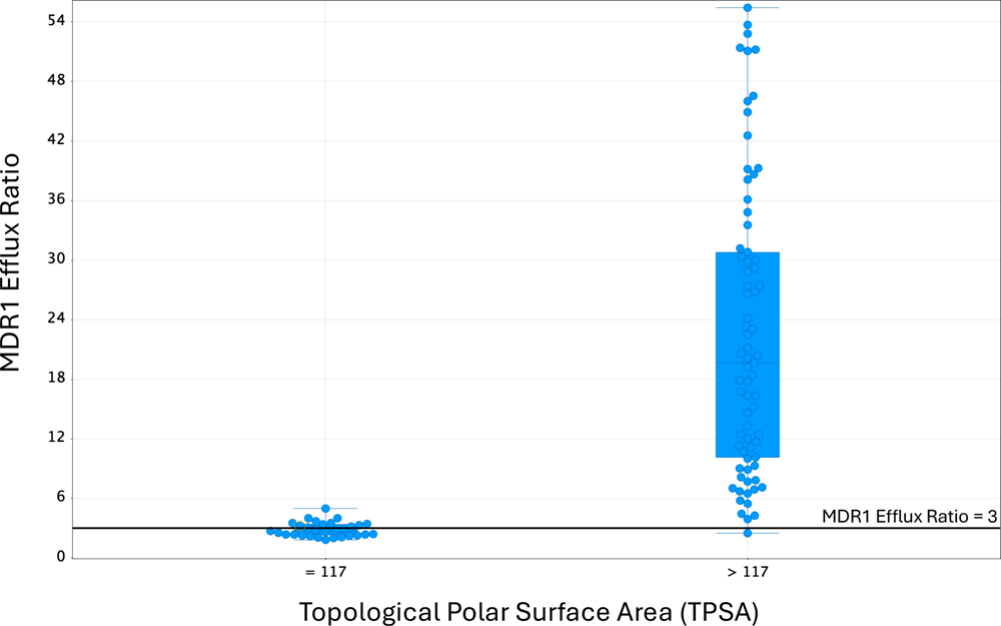
Box plot of MDR1 efflux ratio vs TPSA for all aminopyrazolopyridine analogs (N = 104). The solid black line represents MDR1 efflux ratio = 3. 18/31 of compounds with no additional polarity beyond the core aminopyrazolopyridine-oxamide core (TPSA = 117 Å^2^) achieve MDR1 efflux ratio < 3, whereas only 1/73 with additional polarity (TPSA > 117 Å^2^) had MDR1 efflux ratio < 3.

With **TNG456** in hand, we had a compound that met the potency and selectivity goals of the program with good *in vitro* metabolic stability, high permeability, low efflux, and low risk of CYP3A4 TDI, that warranted further profiling. As for the tunable properties we considered for improving BBB penetration, the values for **TNG456** were: HBD = 3, TPSA = 117 Å^2^, MW = 406, pK_a_ = 6.1, measured logD = 2.4. While **18** also met the target profile for the program and was an equally good candidate, **TNG456** was given priority for advanced study.

## Biochemical Characterization

Like vopimetostat, **TNG456** exhibited potency which was below the detection limits of both the fluorescence polarization peptide displacement and the radiometric FlashPlate assays, in the presence or absence of MTA. Surface plasmon resonance (SPR) binding assays were not informative due to the extraordinarily slow dissociation rate of **TNG456**, which necessitated extended dissociation measurement times making the stability of the PRMT5 enzyme complex on the SPR sensor chip a concern. Consequently, as with vopimetostat,^7^ an enzyme activity recovery assay was employed to biochemically determine the rate of compound dissociation from the enzyme–inhibitor complex, thereby enabling estimation of potency boundaries for **TNG456**. Without MTA, **TNG456** exhibited an estimated apparent inhibition constant (K_i,app_) of approximately 30 pM. Upon addition of MTA, a further reduction in the initial apparent enzymatic rate was observed, consistent with enhanced potency of **TNG456** in the presence of MTA. However, accurate K_i_ determination is challenging because the initial enzymatic rate approached the background control signal in which no enzyme is present. Based on these data, the K_i,app_ of **TNG456** for PRMT5•MTA is estimated to be < 2 pM.

## Further *In Vitro* Characterization

**TNG456** was profiled for off-target activity against a panel of 40 methyltransferases at 10 μM and had no significant activity other than with PRMT5:MEP50 (see the ). **TNG456** was also tested in an *in vitro* toxicology safety panel (SAFETYscan E/IC_50_) of 78 known off-target binding and functional assays up to 30 μM and had no activity of concern (see the ). In a hERG SyncroPatch assay, **TNG456** had an IC_50_ = 9.4 μM representing nearly a 100-fold safety margin relative to the targeted MTAP-null GI_90_ (100 nM).

In 7-day viability studies using *MTAP*-isogenic cell line pairs spanning multiple histologies, **TNG456** had an average 55-fold selectivity for MTAP-null cancer cells compared to their MTAP WT counterparts (Table 4).

**Table 4:** Cellular Viability^a^ Data in Four *MTAP*-Isogenic Cell Lines

	GI_50_ ± SD (μM)		
Cell Line (Histology)	MTAP-null	MTAP WT	Selectivity (fold)
LU99 (NSCLC)	0.040 ± 0.012	1.5 ± 0.16	38
LN18 (GBM)	0.054 ± 0.007	3.5 ± 1.2	65
HCT116 (CRC)	0.015 ± 0.005	0.98 ± 0.098	65
HAP1 (CML)	0.022 ± 0.005	1.1 ± 0.43	50
			Average = 55

a Viability growth inhibition assessed after 7 days using a CellTiter-Glo luminescence-based assay in the indicated *MTAP*-isogenic cell line pairs.

The broad selectivity of **TNG456** relative to TNG908, vopimetostat, and the SAM-uncompetitive PRMT5 inhibitor pemrametostat was further confirmed in a large cancer cell line panel comprised of 72 MTAP WT and 71 MTAP-null cancer cell lines spanning multiple histologies (Figure 9). These results further highlighted the excellent MTAP-dependent selectivity profile of **TNG456**.

**Figure 9:**
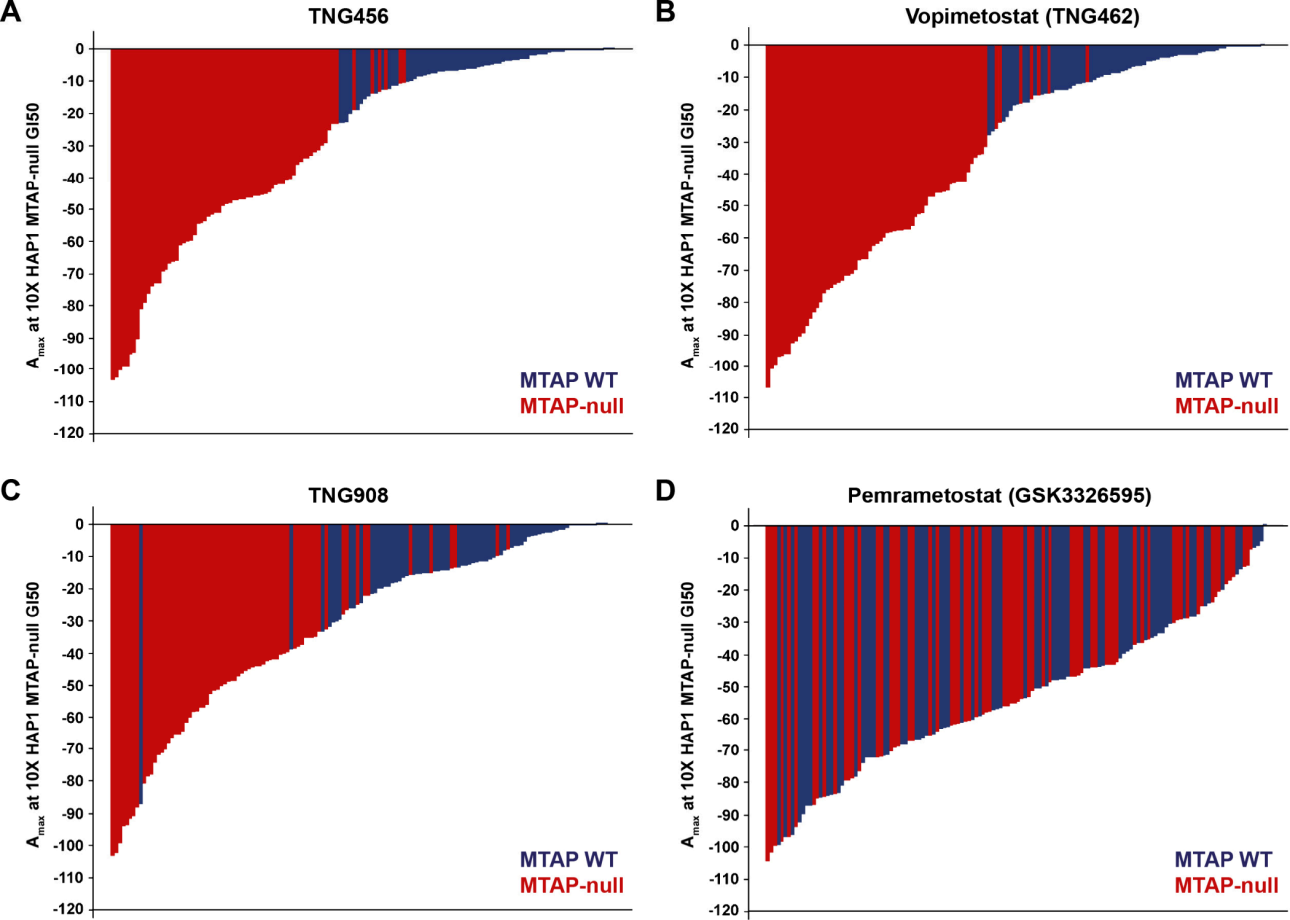
**TNG456** (A) in a 143-cancer cell line panel, using a 7-day viability assay, demonstrating excellent selectivity for MTAP-null cell lines relative to vopimetostat (B), TNG908 (C), and pemrametostat (a SAM-uncompetitive PRMT5 inhibitor; GSK3326595) (D). Maximum effect relative to the HAP1 MTAP-null cell line. 72 MTAP WT cell lines and 71 MTAP-null cell lines.

## Pharmacokinetic Profiling of TNG456 in Preclinical Species

The PK properties of **TNG456** were evaluated in beagle dogs and cynomolgus monkeys (Table 5). Following a 1 mg/kg IV dose in beagle dogs (n = 3), clearance was 3.0 mL/min/kg, volume of distribution was 1.5 L/kg, and the terminal half-life was 6.4 h. Oral administration of a 3 mg/kg dose to beagle dogs resulted in a C_max_ of 0.802 μg/mL, AUC_inf_ of 9.2 h-μg/mL, with 55% bioavailability. In cynomolgus monkeys (n = 3), a 1 mg/kg IV dose of **TNG456** gave a clearance of 16 mL/min/kg, volume of distribution of 4.0 L/kg, and half-life of 3.9 h. Oral administration at 3 mg/kg produced a C_max_ of 0.366 μg/mL, AUC_inf_ of 2.55 h-μg/mL, and 85% bioavailability. The predicted human half-life of **TNG456** is approximately 6 h, suggesting that a twice-daily (BID) dosing regimen would maintain exposures with a C_max_/C_min_ ratio ≤ 2. This regimen is projected to sustain concentrations above the MTAP-null GI_90_ at trough while keeping peak exposures below those expected to affect MTAP WT cells. Brain penetration of **TNG456** was confirmed in two independent cynomolgus monkey CSF studies, which demonstrated mean K_p,uu_ values of 0.59 (0.57 and 0.61 in individual studies). Consistent CNS exposures were also observed in beagle dogs, with brain and time-matched plasma samples collected at the end of a non-GLP dose-range finding toxicity study with K_p,uu_ values averaging 0.64 (ranging from 0.44 to 1.1).

**Table 5:** *In Vivo* PK Characterization of **TNG456**

Species	CL (mL/min/kg)	V_d,ss_ (L/kg)	T_1/2_ (h)	%F	PPB^c^	K_p,uu_
Dog^a^	3.0	1.5	6.4	55	10	0.64
Cynomolgus monkey^b^	16	4.0	3.9	85	19	0.59

a IV/PO dosing in beagle dog (vehicle, IV: 1 mg/kg solution of 1% v/v DMSO/99% of 20% w/v HP-β-CD in saline; PO: 3 mg/kg suspension in 0.5% MC, n = 3 per arm).

b IV/PO dosing in cynomolgus monkey (vehicle, IV: 1 mg/kg solution of 1% v/v DMSO/99% of 20% w/v HP-β-CD in saline; PO: 3 mg/kg suspension in 0.5% MC, n = 3 per arm).

c Plasma Protein Binding, % unbound.

## TNG456 Shows Strong Antitumor Efficacy in MTAP-Null Models

The pharmacodynamic activity of **TNG456** was evaluated in the U87MG MTAP-null glioblastoma (GBM) cell line-derived xenograft model inoculated subcutaneously in BALB/c nude immunocompromised mice. Tumor-bearing mice were either dosed by oral gavage with vehicle or **TNG456** at well-tolerated doses (3, 10, 30, or 90 mg/kg BID), resulting in dose-dependent plasma exposures. Following 7-days of dosing, tumors were collected at 8 or 24 h postlast dose and levels of symmetric dimethylarginine (SDMA)-modified protein levels were quantified by immunoblot analysis. Dose-dependent decreases in SDMA-modified protein levels were observed following **TNG456** treatment, with greater than 90% inhibition achieved at doses ≥ 30 mg/kg BID (Figure 10A). Given previous evidence that > 90% inhibition of PRMT5 activity is required for efficacy,^25^ these data suggest that doses ≥ 30 mg/kg BID are predicted to be efficacious. Strong, dose-dependent antitumor activity was demonstrated with **TNG456** in the U87MG CDX model at well tolerated doses. Consistent with the results of the PK/PD study, the strongest efficacy was observed at doses ≥ 30 mg/kg BID (84% tumor growth inhibition (TGI) at 30 mpk BID and 56% tumor regression at 90 mpk BID) (Figure 10B–C).

**Figure 10:**
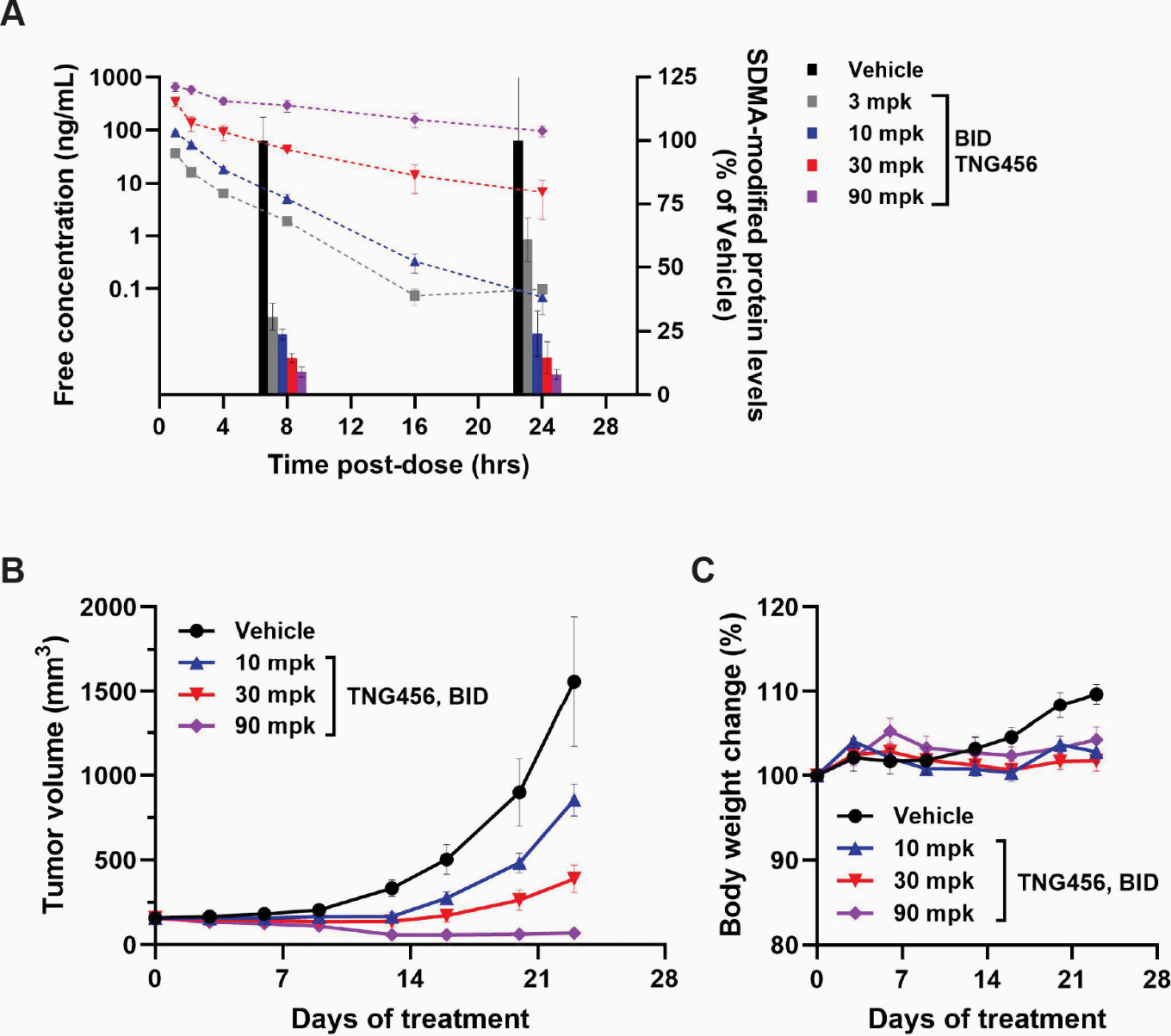
**TNG456** treatment drives dose-dependent PD activity and efficacy in the U87MG MTAP-null GBM xenograft model. A) PK/PD analysis of **TNG456** following compound treatment for 7 days. PRMT5 activity by quantification of a single SDMA-modified protein substrate and free **TNG456** plasma concentrations were determined at the indicated time points postlast dose. N = 4 tumors/time point/dose level. Data are presented as mean ± SEM for the PD analyses. B) Efficacy and C) tolerability were determined for **TNG456** at the indicated dose levels for 23 days. N = 8 mice/group. Data are presented as mean ± SEM.

To evaluate potential histology-dependent differences in response, **TNG456** was profiled across a panel of 13 MTAP-null patient-derived xenograft (PDX) models representing tumor types with high frequencies of *MTAP*-deletion. Oral administration of **TNG456** (90 mg/kg BID) drove strong antitumor activity across all PDX models evaluated, with 5 of 13 (38%) showing 72–99% TGI and 8 of 13 (62%) demonstrating tumor shrinkage including complete and durable responses in nonsmall cell lung cancer (NSCLC) and glioblastoma (GBM) models that were maintained after treatment cessation (Figure 11).

**Figure 11:**
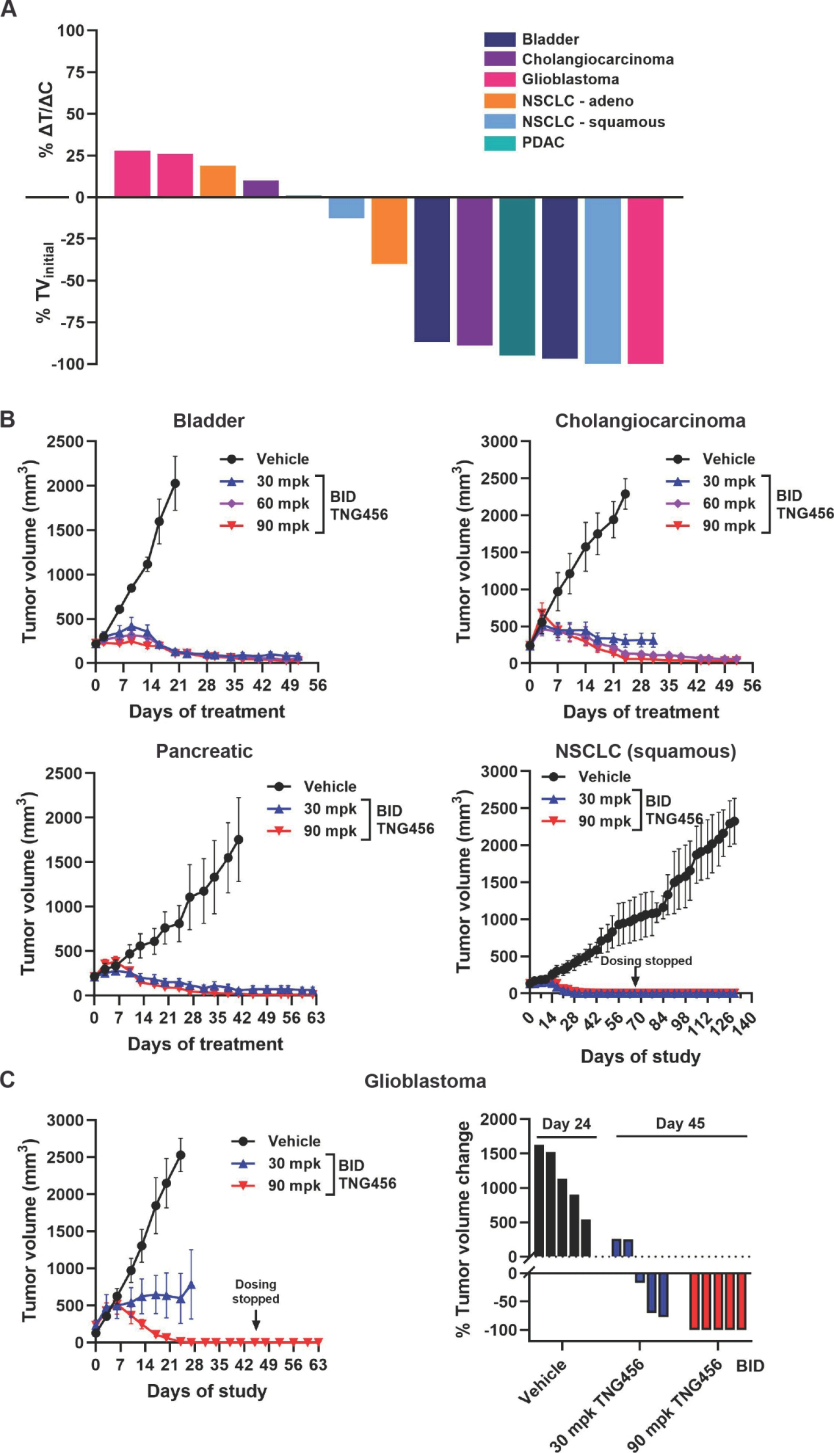
**TNG456** is efficacious in a panel of MTAP-null PDX models representing clinically relevant indications. A) **TNG456** was dosed orally at 90 mg/kg BID. N = 3–5 mice/group/model. B) Selected PDX models from A) representing the indicated tumor types. For the NSCLC (squamous) PDX model, **TNG456** was dosed at either 30 or 90 mg/kg BID until day 66 when treatment was withdrawn. The mice were observed until day 129. Data are presented as mean ± SEM. C) Selected GBM PDX model from A). **TNG456** was dosed until day 45 when treatment was withdrawn. The mice were observed until day 63. Data are presented as mean ± SEM. Percent tumor volume change is reported at either Study Day 24 (Vehicle-treated mice) or Day 45 (**TNG456**-treated mice).

Collectively, these data demonstrate that **TNG456** exerts potent, on-target, and dose-dependent antitumor activity across diverse *MTAP*-deleted models representing multiple tumor histologies including CNS malignancies. **TNG456** monotherapy achieved tumor regressions, including complete responses, in the majority of PDX models tested, underscoring its potential to deliver meaningful clinical efficacy in patients with *MTAP*-deleted cancers.

## Studies with TNG456 and Abemaciclib in MTAP-Null Efficacy Models Show Strong Combination Benefit

Although **TNG456** monotherapy demonstrated striking efficacy and tolerability in preclinical xenograft studies, combination therapies in the clinic may promote deeper and more durable responses by overcoming drug resistance or tumor heterogeneity.^26^ Glioblastoma is well-known for its pronounced heterogeneity, contributing to treatment challenges and poor prognosis.^27^ All *MTAP*-deleted tumors are expected to harbor *CDKN2A*-deletion which has been shown to sensitize cells to CDK4/6 inhibition.^12^ Since abemaciclib is a clinical brain-penetrant CDK4/6 inhibitor, we investigated whether combination therapy with **TNG456** would enhance antitumor activity. In highly aggressive MTAP-null glioblastoma xenograft models, where neither **TNG456** nor abemaciclib monotherapy induced tumor regressions, the combination yielded significant therapeutic benefit. In both the AM38 CDX or a GBM PDX model, **TNG456** was administered either at a subtherapeutic dose with a clinically relevant dose of abemaciclib (150 mg QD), or at a therapeutic dose in combination with a reduced abemaciclib dose. Regardless of the dosing regimen, robust combination benefit was observed, including complete responses in the GBM PDX model (Figure 12). Notably, abemaciclib is a sensitive CYP3A4 substrate, emphasizing the importance of a development candidate with low CYP3A4 DDI risk, a criterion met by **TNG456**.

**Figure 12:**
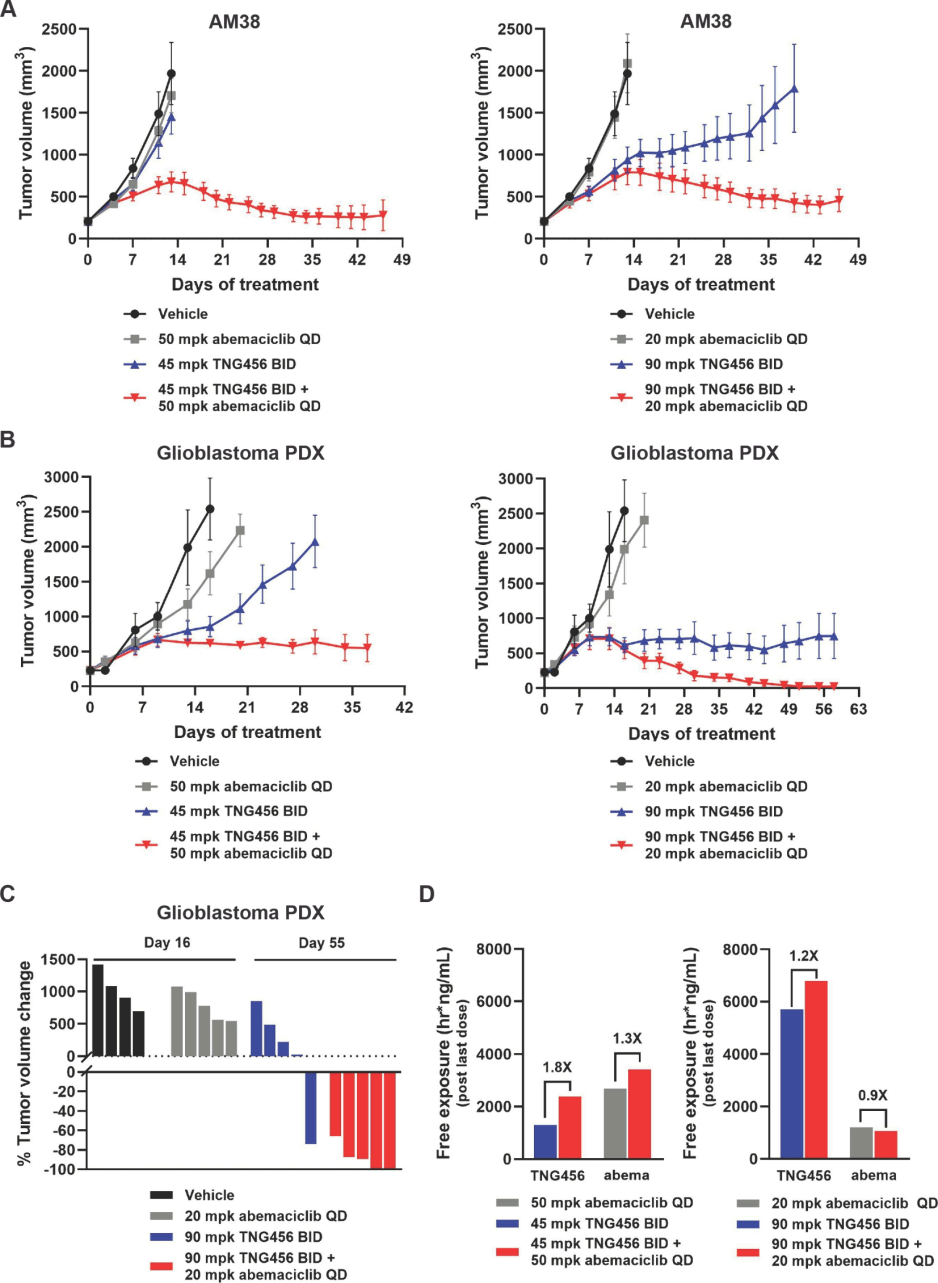
Combination of **TNG456** with abemaciclib drives a strong benefit in glioblastoma xenograft models. A-B) **TNG456** was dosed orally at either 45 or 90 mg/kg BID; abemaciclib was dosed orally at either 20 or 50 mg/kg QD. N = 8 mice/group in the AM38 MTAP-null GBM CDX model A) or n = 5 mice/group in an MTAP-null GBM PDX model B). Data are presented as mean ± SEM. C) Percent tumor volume change is reported at either Study Day 16 (Vehicle-treated or abemaciclib-treated mice) or Day 55 (**TNG456**-treated or combination-treated mice) from panel B). D) Terminal free plasma exposures from A). All treatments were well-tolerated.

## Synthesis

Final oxamide analogs were prepared through two general methods. Most were prepared through an Ullmann–Goldberg type reaction (Scheme 1) in which 7-bromo-1*H*-pyrazolo­[4,3-*c*]­pyridin-4-amine, protected with either a tetrahydropyran (THP) or 2-(trimethylsilyl)­ethoxymethyl (SEM) group, was coupled to the appropriate primary oxamide in the presence of copper, copper iodide, cesium carbonate, and (1*R, 2R)*-*N* ^1^, *N* ^2^-dimethylcyclohexane-1,2-diamine in refluxing dioxane followed by the removal of the protecting group with 4 M HCl in dioxane. Chiral chromatography was used to separate the two enantiomers when racemic amides were used. Compounds **8** and **9** were prepared through amide coupling of the appropriate oxamic acid with dibenzylamine using HATU and diisopropylethylamine in dimethylformamide (Scheme 2), with deprotection through refluxing dioxane for **9**.

**Scheme 1:**
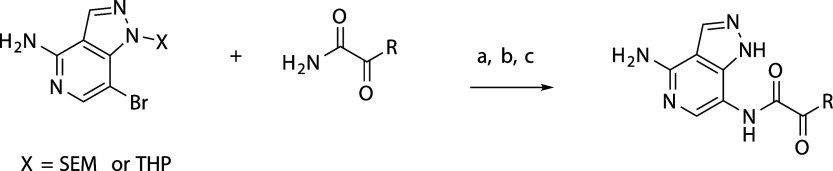
General Synthesis of Oxamides: Compounds **3**–**7**, **12**–**23**, **TNG456**

a Reagents and conditions: (a) Cu, CuI, Cs_2_CO_3_, (*1R*, *2R*)-*N1, N2*,-dimethylcyclohexane-1,2-diamine, dioxane, 100 °C, 18 h; (b) 4 M HCl in dioxane, rt, 1 h; (c) chiral chromatography (when appropriate).

**Scheme 2:**
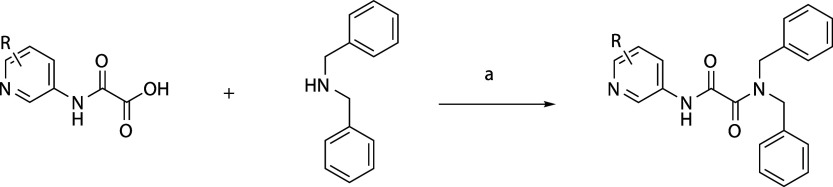
General Synthesis of Oxamides: Compounds **8** and **9**

a Reagents and conditions: (a) HATU, DIPEA, DMF, rt, 1h.

The primary oxamides used in the Ullmann–Goldberg couplings were prepared by coupling the appropriate amine with 2,2,2-trifluoroethyl 2-chloro-2-oxoacetate or its methyl analog in the presence of triethylamine in THF to give the oxamic ester which was then converted to the primary oxamide with ammonia in THF.

Scheme 3 shows the synthesis of 2-((2*R*,5*S*)-5-methyl-2-phenylpiperidin-1-yl)-2-oxoacetamide (**28**), exemplifying the procedure used to prepare the primary oxamide-piperidine analogs. Ring opening of *tert*-butyl (*S*)-5-methyl-2-oxopiperidine-1-carboxylate with phenyl magnesium bromide in THF yielded *tert*-butyl (*S*)-(2-methyl-5-oxo-5-phenylpentyl)­carbamate, **24**. Boc removal and ring closing to the imine, **25**, was achieved through the treatment with TFA in DCM. Reduction with NaBH_4_ in MeOH led to (2*R*,5*S*)-5-methyl-2-phenylpiperidine, **26**, typically in a 9:1 ratio of *trans*:*cis*. Subsequent oxamic ester and primary oxamide (**27** and **28**, respectively) formation was done as described. Varying the Grignard reagent would deliver the other related analogs.

**Scheme 3:**
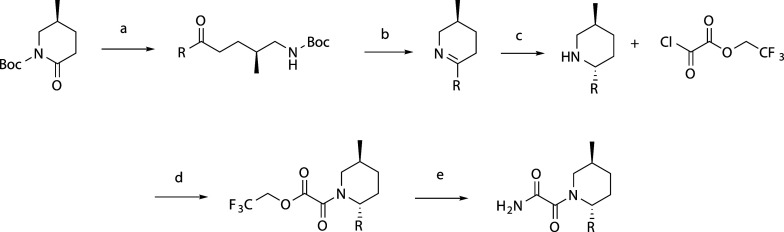
Representative Synthesis of Substituted Piperidine Oxamides

a Reagents and conditions: (a) RMgBr, THF, −78 °C → rt, 1 h (**24,** R = Ph); (b) TFA, DCM, rt, 1 h (**25,** R = Ph); (c) NaBH_4_, MeOH, 0 °C → rt, overnight (**26,** R = Ph); (d) TEA, THF (**27,** R = Ph); (e) NH_3_, THF (**28,** R = Ph).

Scheme 4 shows the synthesis of **TNG456**. (*R*)-*N*-methyl-1-(4-(trifluoromethyl)­phenyl)­ethan-1-amine was coupled to methyl 2-chloro-2-oxoacetate in DCM in the presence of triethylamine to afford methyl (*R*)-2-(methyl­(1-(4-(trifluoromethyl)­phenyl)­ethyl)­amino)-2-oxoacetate (**29)**. Treatment with ammonia in THF yielded (*R*)-*N* ^1^-methyl-*N* ^1^-(1-(4-(trifluoromethyl)­phenyl)­ethyl)­oxalamide (**30)**. Ullmann–Goldberg type coupling with THP protected 7-bromo-1*H*-pyrazolo­[4,3-*c*]­pyridin-4-amine as described above and subsequent deprotection with HCl in dioxane yielded **TNG456**.

**Scheme 4:**
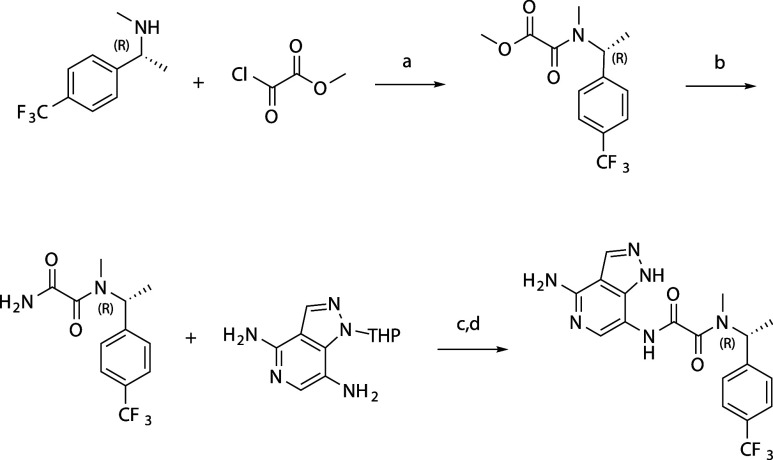
Synthesis of **TNG456**

a Reagents and conditions: (a) TEA, DCM, rt, 1 h; (b) NH_3_, THF, rt, 18 h; (c) Cu, CuI, Cs_2_CO_3_, (*1R*, *2R*)-*N1, N2*,-dimethylcyclohexane-1,2-diamine, dioxane, 100 °C, 48 h; (d) 4 M HCl in dioxane, rt, 1 h.

## Conclusions

In summary, a series of aminopyrazolopyridine oxamide compounds was developed. Through strategic optimization, leveraging the potency of the aminopyrazolopyridine motif, while minimizing MW and limiting polarity beyond the core scaffold, we achieved a balance of properties culminating in the highly potent and selective candidate **TNG456**. Moreover, **TNG456** has high potential to be brain penetrant in humans based on preclinical *in vitro* and *in vivo* data. **TNG456** exhibits a PRMT5•MTA K_i,app_ < 2 pM, HAP1 MTAP-null GI_50_ = 20 nM with 50-fold selectivity vs HAP1 MTAP WT cells. Its high permeability (13 × 10^–6^ cm/s in MDCK cells), low MDR1 efflux ratio (2.6), and K_p,uu_ ranging from 0.5 to 1.0 across preclinical species (mean 0.6) supports its potential to achieve therapeutically relevant CNS exposure in patients. It also demonstrates a favorable CYP3A4 profile, with no preclinical signals of CYP3A4 inhibition, time-dependent inhibition, or induction suggesting a very low risk of clinical CYP3A4 mediated DDIs. This feature supports the feasibility of combination therapies, including with abemaciclib, a sensitive CYP3A4 substrate. Finally, the preclinical DMPK profile of **TNG456** predicts human exposure sufficient to maintain MTAP-null GI_90_ coverage in the brain at trough supporting its advancement as a clinical candidate. On this basis, **TNG456** was nominated for development with Phase I/II clinical studies initiated in 2025 (NCT05732831).

## Experimental Section

### General Procedures

All chemicals were provided by Enamine Ltd., WuXi AppTec, or other commercial suppliers and used as received unless otherwise indicated. All solvents were treated according to standard methods. All reactions were monitored by LC-MS analysis using Agilent 1260 LC/MSD instruments with an Agilent Poroshell 120 SB-C18 4.6 × 30 mm 2.7 μm column, column Temperature: 60 °C, mobile phase: A – water (0.1% formic acid), B – acetonitrile (0.1% formic acid), flow rate: 1.5 mL/min, gradient: 0.01 min – 1% B, 5.00 min – 100% B, 5.99 min – 100% B, MS Ionization mode: Electrospray ionization (ESI), MS Scan range: 83–1000 *m*/*z*, UV detection: 215 nm, 254 nm, 280 nm unless otherwise specified. Thin-layer chromatography (TLC) with precoated silica gel GF254 (0.2 mm) was used, and the results were visualized using either UV light or KMnO_4_ stain. Proton nuclear magnetic resonance (^1^H NMR) spectra were recorded at 400, 500, or 600 MHz on Varian or Bruker instrumentation; chemical shifts were calibrated using residual nondeuterated solvents CHCl_3_ (δ = 7.26 ppm), DMSO (δ = 2.50 ppm) or MeOH (δ = 3.31 ppm) and expressed in δ ppm. Coupling constants (*J*), when given, are reported in hertz (Hz). Multiplicities are reported using the following abbreviations: s = singlet, d = doublet, dd = doublet of doublets, t = triplet, q = quartet, m = multiplet (range of multiplets is given), br = broad signal, dt = doublet of triplets. ^19^F-NMR spectra were recorded at 376 MHz (Varian), ^13^C NMR spectra were recorded at 101, 126, or 151 MHz (Varian). ^13^C NMR chemical shifts are reported relative to the central CHCl_3_ (δ = 77.16 ppm), DMSO (δ = 39.52 ppm) or MeOH (δ = 49.00 ppm) and chemical shifts are reported in parts per million (ppm). All final compounds were purified by reverse phase high-performance liquid chromatography (HPLC) or supercritical fluid chromatography (SFC) or silica gel chromatography (100–200 mesh). HPLC was done with an Agilent 1260 HPLC instrument (Agilent Technologies, Germany) equipped with a G7161A Preparative Binary Pump, a G7157A Prep Autosampler, a G7115A DAD WR and a G7159B Preparative Fraction Collector. The Open Lab CDS software (version C.01.10 was used for instrument control, data acquisition and data handling. SFC was done with a Waters 100q Prep SFC System. Chiral HPLC analytical analysis was done with an Agilent 1200 HPLC instrument (Agilent Technologies, Germany) equipped with a G1379B degasser, a G1312A Binary Pump, a G1329A ALS autosampler, a G1315A Diode Array Detector. Chiral SFC analytical analysis was done with an Agilent 1260 SFC instrument (Agilent Technologies, Germany) equipped with a G1379B degasser, a G1312B Binary Pump, a G1313A ALS autosampler, a G1316A thermostated column compartment, a G1315D Diode Array Detector and an Aurora SFC system. Melting points were taken using OptiMelt Automated Melting Point System Digital Image Processing Technology SRS Stanford Research Systems, 2 °C/min (5 °C/min at high melting point). Optical rotation was measured with Rudolph Autopol V/Mettler Toledo XSR205DU. Standard conditions for analysis: solution concentration 0.5 g/50 mL (methanol solvent), wavelength 589 nm, temperature 25 °C. All oxamides exist as rotamers in ^1^H NMR spectra. All compounds are >95% pure by HPLC.

#### General Procedure for Ullmann–Goldberg Type Reaction in Scheme 1

Oxamide starting material (246.8 mg, 785.2 μmol, 1 equiv), protected 7-bromo-1*H*-pyrazolo­[4,3-*c*]­pyridin-4-amine (1.5 equiv), Cu (0.1 equiv), CuI (0.3 equiv), cesium carbonate (1.5 equiv) and (1*R*,2*R*)-*N1*,*N2*-dimethylcyclohexane-1,2-diamine (0.3 equiv) were mixed in dioxane (0.2 M), purged with Ar for 2 min and then heated in a sealed tube at 100 °C for 18 h. The mixture was filtered and the filtrate was evaporated in vacuo to afford the desired oxamide, followed by the removal of the protecting group with 4 M HCl in dioxane. Chiral chromatography was used to separate the two enantiomers when racemic amides were used.

*Copper was prepared by the following procedure: Zn powder (2 equiv) was added slowly under intensive stirring to a 10% solution of Cu­(NO_3_)_2_ (10 equiv) in water. The mixture was stirred for 90 min, then the resulting solid was filtered, washed with a 10% solution of Cu­(NO_3_)_2_, and distilled water. The solids were dried for 5 h at 80 °C to give a black powder of copper

**1,4-Dioxane was dried over molecular sieves.

***The insoluble materials must be removed with centrifuge before HPLC.

#### General Procedure for Amide Coupling in Scheme 2

Amine (1 equiv) was dissolved in DCM (0.2 M) and TEA (2.5 eq or 3.5 eq if amine is a salt) was added. The reaction mixture was cooled and 2,2,2-trifluoroethyl 2-chloro-2-oxo-acetate (1 equiv) was added dropwise. The reaction was stirred at room temperature overnight. Water was added and the organic layer was washed with brine, dried over Na_2_SO_4_, and concentrated under vacuum to afford the oxamic ester which was dissolved in MeOH/NH_3_ (0.2 M) and stirred overnight at room temperature. The solution was concentrated under vacuum to afford the oxamide.

#### *tert*-Butyl (*S*)-(2-methyl-5-oxo-5-phenylpentyl)­carbamate (**24**)

To a dry 2-necked flask was added THF (150 mL) and *tert-*butyl (*5S*)-5-methyl-2-oxo-piperidine-1-carboxylate (8.00 g, 37.51 mmol) with stirring and the solution was cooled to −78 °C. Phenyl magnesium bromide in THF (1.5 equiv) was added to the *Boc*-lactam over 1 h, maintaining the internal temperature below −70 °C. The solution was warmed to room temperature and sat. NH_4_Cl (aq) was added. The aqueous layer was extracted with DCM (3 × 50 mL) and the organic layers combined, dried over Na_2_SO_4_, filtered and concentrated in vacuum. *tert*-butyl (*S*)-(2-methyl-5-oxo-5-phenylpentyl)­carbamate (**24**) (12 g, crude) was obtained as a light-yellow oil and was used in the next step without further purification. ^1^H NMR (400 MHz, CDCl_3_) δ 0.93 (d, 3H), 1.44 (s, 9H), 1.57 (m, 4H), 3.01 (m, 4H), 7.45 (m, 3H), 7.93 (d, 2H).

#### (*S*)-3-Methyl-6-phenyl-2,3,4,5-tetrahydropyridine (**25**)

A solution of **24** (1.0 equiv) in TFA was stirred at 25 °C for 1 h and then concentrated under vacuum. Crushed ice was added to the residue, and the resulting mixture was basified to pH 10 with 10% aqueous potassium carbonate solution and extracted with DCM (2x). The combined organic extracts were dried over sodium sulfate and concentrated under reduced pressure to afford (*S*)-3-methyl-6-phenyl-2,3,4,5-tetrahydropyridine (**25**), 84% yield. ^1^H NMR (500 MHz, CDCl_3_) δ 0.99 (d, 3H), 1.39 (m, 1H), 1.73 (m, 1H), 1.92 (m, 1H), 2.58 (m, 1H), 2.77 (m, 1H), 3.26 (m, 1H), 3.99 (m, 1H), 7.37 (m, 3H), 7.78 (m, 2H).

#### (2*R*,5*S*)-5-Methyl-2-phenylpiperidine (**26**)

**25** (1 equiv) was dissolved in MeOH and the resulting solution was cooled to 0 °C in an ice bath. Sodium borohydride (2 equiv) was added portion-wise to the solution and upon completion the reaction mixture was allowed to warm to room temperature and stirred overnight. Water was added and the resulting mixture was concentrated under vacuum. The residue was diluted with water and extracted with DCM (2X), dried over Na_2_SO_4_, filtered, and concentrated to obtain (2*R*,5*S*)-5-methyl-2-phenylpiperidine (**26**), 91% yield. ^1^H NMR (500 MHz, CDCl_3_) δ 0.90 (d, 3H), 1.15 (m, 1H), 1.78 (m, 2H), 1.82 (m, 3H), 2.42 (m, 1H), 3.13 (m, 1H), 3.55 (m, 1H), 7.35 (m, 5H).

#### 2,2,2-Trifluoroethyl 2-((2*R*,5*S*)-5-methyl-2-phenylpiperidin-1-yl)-2-oxoacetate (**27**)

**26** (1 equiv) and TEA (1.1 equiv) were dissolved in THF and cooled to 0 °C followed by the dropwise addition of 2,2,2-trifluoroethyl 2-chloro-2-oxo-acetate (1.1 equiv) under Ar atomosphere. The reaction mixture was stirred for 12 h at room temperature and evaporated under reduced pressure to give 2,2,2-trifluoroethyl 2-((2*R*,5*S*)-5-methyl-2-phenylpiperidin-1-yl)-2-oxoacetate (**27**) which was used in the next step without further purification, 61% yield. LCMS (ESI): [M]^+^ *m*/*z*: calcd 329.2; found 330.2; Rt = 2.657 min.

#### 2-((2*R*,5*S*)-5-Methyl-2-phenylpiperidin-1-yl)-2-oxoacetamide (**28**)

Ammonia was bubbled through a solution of **27** (1 equiv) in THF for 10 min at 0 °C. The reaction mixture was then stirred for 18 h at room temperature. The reaction mixture was filtered, and the filtrate was concentrated under vacuum to give 2-((2*R*,5*S*)-5-methyl-2-phenylpiperidin-1-yl)-2-oxoacetamide (**28**) which was used in the next step without further purification, 65% yield. HPLC conditions: Column: YMC Triart C18 100 × 20 mm, 5 μM; 0–5 min 15–35% water-MeCN + 0.1% NH_4_OH 30 mL/min; (loading pump 4 mL/min MeCN). LCMS (ESI): [M]^+^ *m*/*z*: calcd 246.2; found 247.2; Rt = 1.150 min.

#### Methyl (*R*)-2-(methyl­(1-(4-(trifluoromethyl)­phenyl)­ethyl)­amino)-2-oxoacetate (**29**)

To a solution of (1*R*)-1-[4-(trifluoromethyl)­phenyl]­ethanamine (4 g, 17.73 mmol, HCl) and TEA (39.00 mmol, 5.44 mL) in THF (50 mL) was added methyl 2-chloro-2-oxo-acetate (2.39 g, 19.50 mmol) at room temperature under an Ar atmosphere. After stirring at room temperature for 1 h the mixture was filtered through a pad of Na_2_SO_4_, evaporated to dryness, added THF (50 mL), and concentrated again. The residue was dissolved in THF and washed with saturated NaHCO_3_ (aq), brine, dried, and evaporated to give methyl 2-[methyl-[(1*R*)-1-[4-(trifluoromethyl)­phenyl]­ethyl]­amino]-2-oxo-acetate, **29** (3.9 g, 13.48 mmol, 76% yield) as a colorless gum. LCMS (ESI): [M + H]+ *m*/*z*: calcd 289.09; found 290.0; Rt = 1.249 min.

#### (*R*)-*N* ^1^-Methyl-*N* ^1^-(1-(4-(trifluoromethyl)­phenyl)­ethyl)­oxalamide (**30**)

Ammonia was bubbled through a solution of **29** (3.9 g, 13.48 mmol) in MeOH (50 mL) at room temperature. After stirring for 18 h, the reaction mixture was evaporated to dryness, dissolved in DCM, and washed with saturated NaHCO_3_ (aq), dried and evaporated to give *N′*-methyl-*N′*-[(1*R*)-1-[4-(trifluoromethyl)­phenyl]­ethyl]­oxamide, **30** (3.4 g, 12.40 mmol, 92% yield) as an orange solid. LCMS (ESI): [M- C3H5N2O2]+ *m*/*z*: calcd 274.09; found 173.2; Rt = 2.770 min.

#### (*R*)-*N* ^1^-(4-Amino-1*H*-pyrazolo­[4,3-*c*]­pyridin-7-yl)-*N* ^2^-methyl-*N* ^2^-(1-(4-(trifluoromethyl)­phenyl)­ethyl)­oxalamide, **TNG456**

**30** (1.5 g, 4.38 mmol), 7-bromo-2-tetrahydropyran-2-yl-pyrazolo­[4,3-*c*]­pyridin-4-amine (1.3 g, 4.38 mmol), copper (13.9 mg, 218.79 μmol), copper­(I) iodide (416.7 mg, 2.19 mmol), (1*R*,2*R*)-*N* ^1^,*N* ^2^-dimethylcyclohexane-1,2-diamine (466.8 mg, 3.28 mmol) and cesium carbonate (2.9 g, 8.75 mmol) were mixed in dioxane (40 mL). The resulting mixture was purged with Ar for 30 s. The vial was sealed and heated at 100 °C for 48 h. The reaction mixture was cooled and filtered. The filter cake was washed with MeOH (100 mL) and the filtrate was concentrated in vacuo. The resulting solid was redissolved in MeOH (25 mL) and HCl (4.0 M in dioxane, 4.38 mmol, 25 mL) was added. After 1 h the reaction mixture was filtered, rinsed with MeOH, and evaporated. The residue was purified by HPLC (column: PFP C18 19 × 100 mm, 5 μM; gradient elution: 23–30–80–100% of water +15 mM NH_4_HCO_3_)-MeOH, 30 mL/min; loading pump 4 mL/min, MeOH) to afford (*R*)-*N* ^1^-(4-amino-1*H*-pyrazolo­[4,3-*c*]­pyridin-7-yl)-*N* ^2^-methyl-*N* ^2^-(1-(4-(trifluoromethyl)­phenyl)­ethyl)­oxalamide, **TNG456** (407 mg, 1.00 mmol, 23% yield). Optical rotation [α]_D_^21^ + 46.3 (*c* = 0.5, MeOH, 21 °C). ^1^H NMR (600 MHz, DMSO-*d*_6_) δ 1.52–1.69 (m, 3H), 2.58–2.88 (m, 3H), 3.40–3.44 (m, 1H), 5.52–5.87 (m, 1H), 6.65 (s, 2H), 7.49–7.59 (m, 1H), 7.62–7.66 (m, 1H), 7.66–7.71 (m, 1H), 7.73–7.78 (m, 2H), 8.15–8.19 (m, 1H), 9.85–12.23 (m, 1H). LCMS (ESI): [M + H]^+^ *m*/*z*: calcd 406.15; found 407.0; Rt = 2.178 min.

### HAP1 MTAP WT and MTAP-null In Cell Western Assay

Detailed methods can be found in Cottrell et al., 2024.^28^ In brief, the HAP1 *MTAP*-isogenic cell line pair was acquired from Horizon Discovery (HZGHC004894c005) and maintained in DMEM (high glucose) + 10% FBS in a humidified, 10% CO_2_ tissue culture incubator. The SAM-cooperative PRMT5 inhibitor, GSK3326595, was sourced from Selleck Chemicals and maintained as a 10 mM DMSO stock. HAP1 MTAP WT and MTAP-null cells were treated with compounds for 24 h in 384-well microtiter plates, and then normalized SDMA levels were determined using a multi-mAb SDMA antibody (Cell Signaling 13222) and DRAQ5 (LiCor 926-32211 and VWR 10761-508). Background signal was determined by signal from wells treated with 1 μM GSK3326595. Data analysis was performed using the 4-parameter logistic (4-PL) Hill equation with maximal effect constrained to 0. The fit was performed using GraphPad Prism or in Dotmatics Studies 5.4 as part of a customized data analysis protocol.

### Cell Line Viability Assays

Detailed methods can be found in Cottrell et al., 2024.^28^ In brief, the HAP1 and HCT116 *MTAP*-isogenic cell line pairs were acquired from Horizon Discovery (HZGHC004894c005 and HD R02-033, respectively), the LU99 and LN18 *MTAP*-isogenic cell line pairs were engineered by stable introduction of a full-length *MTAP* cDNA under the control of a UbiC promoter. All cell lines were maintained in DMEM (high glucose) + 10% FBS in a humidified, 10% CO_2_ tissue culture incubator and confirmed for their MTAP status by immunoblot. Cell viability was determined by CellTiter-Glo following 7-days of compound treatment. Data are plotted as % of the DMSO control wells and fit using a 4-parameter logistic (4-PL) Hill equation with maximal effect or baseline constrained to 0. The fit was performed using GraphPad Prism or the default IC_50_ fitting procedure in Dotmatics Studies 5.4 as part of a customized data analysis protocol. Absolute IC_50_s are reported for each cell line. For the 143-cancer cell line panel potency is reported as a relative IC_50_ as determined by a 4-parameter logistic (4-PL) Hill equation (GraphPad Prism) and selectivity was visualized by plotting the maximum effect (Amax) of **TNG456** at 2200 nM according to the curve fit.

### In Vivo Pharmacology

All protocols for *in vivo* pharmacology studies were approved by the relevant Institutional Animal Care and Use Committees (Pharmaron, Beijing, China; CrownBio, San Diego, CA, and Taicang and Beijing, China; Champions Oncology, Rockville, MD; and XenoSTART, San Antonio, TX; following the guidance of the Association of Assessment and Accreditation of Laboratory Animal Care. Following acclimatization, cancer cells were injected subcutaneously into the right flank of 6- to 8-week-old female BALB/c nude mice and allowed to form palpable tumors. Mice were randomized to treatment groups with a mean tumor volume of approximately 160 mm^3^ (U87MG efficacy), 325 mm^3^ (U87MG PK/PD) or 205 mm^3^ (AM38 combination efficacy) in size. **TNG456** was formulated in 5% DMA/20% Captisol. Abemaciclib was formulated in 1% hydroxyethyl cellulose +0.1% antifoam in 25 mM PB pH 2. PDX studies were conducted with similar study designs. The glioblastoma PDX models used in Figure 11 and Figure 12 are two different models. Tumor volumes were measured using calipers and calculated as (length × width × width)/2. For data analysis, tumor growth inhibition % TGI = [1 – (Treated TV_final_ – Treated TV_initial_)/(Vehicle TV_final_ – Vehicle TV_initial_)] × 100; tumor regression % TV= [mean TV_final_ – mean TV_initial_] × 100. Tumor volume data were analyzed using GraphPad Prism software.

### Western Blotting

Protein lysates were generated by lysis of frozen tumor tissue using RIPA buffer. Samples were normalized by protein concentration using Pierce Rapid Gold BCA Protein Assay Kit (A53225). SDS-PAGE was run using Invitrogen NuPAGE 4–12% Bis-Tris Midi Protein Gels (WG1402BOX). Antibodies SDMA (CST#13222), ACTB (CST#3700) were used at 1:1000 dilution.

All animal studies were conducted in accordance with protocols approved by the Institutional Animal Care and Use Committee (IACUC) of WuXi AppTec (Suzhou) Co. The corresponding IACUC case numbers include NJ-20211026-Dogs, SZ20220524-Monkeys-A, NJ-20221109-Dogs, SZ20230409-Dogs, NT20240325-Dogs, PK02-001-2021v1.0, PK01-001-2021v1.0, PK01-001-2021v1.4, PK02-001-2021v1.8, and PK02-SH003-2023v1.2.

## Supplementary Material





## Notes

Molecular formula strings (CSV)
General experimental and chemical procedures; NMR spectra and HPLC/LCMS traces of final compounds; Analytical data for TNG456; Small molecule crystal structure of TNG456; Intramolecular hydrogen bonding for a model aminopyrazolopyridine oxamide; Prediction of conformational restriction of *N*-methylbenzyl, *R*-methylbenzyl, and *S*-methylbenzyl oxamides; Docking of fixed conformations of *R*-methylbenzyl and *S*-methylbenzyl; Biochemical recovery assay; Methyltransferase panel data for TNG456; Eurofins SAFETYscan panel for TNG456; MDR1-MDCKII assay; Human liver microsomes assay; Hepatocyte stability assay; hERG assay; *in vivo* PK; Plasma protein binding assay; In cell western assay; Cellular viability assay; *in vivo* pharmacology; Western blotting; PRMT5:MEP50 expression and purification; PRMT5:MEP50 crystallography (PDF)

## Notes

Authors will release the atomic coordinates upon article publication.

## Abbreviations

ADME: absorption, distribution, metabolism, and excretion
BBB: blood–brain barrier
BID: *bis in die*
CL: clearance
CNS: central nervous system
CPCM: conductor-like polarizable continuum model
CSF: cerebrospinal fluid
DDI: drug–drug interaction
DFT: density functional theory
DMPK: drug metabolism and pharmacokinetics
GBM: glioblastoma multiforme
GI_50_: growth inhibition 50%
GLP: good laboratory practice
HBD: hydrogen bond donor
hERG: human ether-a-go-go related gene
IMHB: intramolecular hydrogen bond
MDCKII: Madin-Darby Canine Kidney-II cells
MDR1: multidrug resistant 1
MTA: 5′-methylthioadenosine
MTAP: methylthioadenosine phosphorylase
PDX: patient-derived xenograft
PRMT5: protein arginine methyltransferase 5
QD: *quaque die*
SAM: *S*-adenosylmethionine
SDMA: symmetric dimethylarginine
SPR: surface plasmon resonance
TGI: tumor growth inhibition
TPSA: topological polar surface area
