# Supplementary material for: The Discovery of TNG456: A Highly Potent, Selective, Brain-Penetrant MTA-Cooperative PRMT5 Inhibitor for the Treatment of MTAP-Deleted Cancers
Source: J Med Chem. 2026 May 18;69(11):12853–69. doi: 10.1021/acs.jmedchem.6c00035 (PMC13266975; doi:10.1021/acs.jmedchem.6c00035)
Supplement: Supplementary file 2 [file jm6c00035_si_002.pdf]

**SUPPORTING INFORMATION**

# THE DISCOVERY OF TNG456: A HIGHLY POTENT, SELECTIVE, BRAIN-PENETRANT MTA-COOPERATIVE PRMT5 INHIBITOR FOR THE TREATMENT OF *MTAP*-DELETED CANCERS

Kevin M. Cottrell*‡, Kimberly J. Briggs‡, Alice Tsai‡, Colin Liang‡, Patrick McCarren‡, Douglas A. Whittington‡, Minjie Zhang‡, Wenhai Zhang‡, Alan Huang‡, Jannik Andersen‡, and John P. Maxwell‡.

‡Tango Therapeutics, Boston, MA 02215, United States
*Corresponding Author kcottrell@tangotx.com

Table of Contents

General experimental and chemical procedures……S1
NMR Spectra and HPLC/LCMS traces of final compounds……S17
Analytical data for **TNG456**……S43
Small molecule crystal structure of **TNG456**……S53
Intramolecular hydrogen bonding for a model aminopyrazolopyridine oxamide……S53
Prediction of conformational restriction of *R*-methylbenzyl and *S*-methylbenzyl……S53
Docking of *R*-methylbenzyl and *S*-methylbenzyl oxamides……S53
Biochemical recovery assay……S55
Methyltransferase panel for **TNG456**……S56
Eurofins SAFETYscan panel for **TNG456**……S57
MDR1-MDCKII assay……S59
Human liver microsomes assay……S60
Hepatocyte stability assay……S60
hERG assay……S60
In vivo PK……S61
Plasma protein binding assay……S61
In cell western assay……S61
Cellular viability assay……S61
In vivo pharmacology……S61
Western blotting……S61
PRMT5:MEP50 expression and purification……S61
PRMT5:MEP50 crystallography……S61

## GENERAL EXPERIMENTAL AND CHEMICAL PROCEDURES

All chemicals were provided by Enamine Ltd., WuXi Apptech, or other commercial suppliers and used as received unless otherwise indicated. All solvents were treated according to standard methods. All reactions were monitored and analysis of final compounds performed by LC-MS using Agilent 1260 LC/MSD instruments, with an Agilent Poroshell 120 SB-C18 4.6 x 30mm 2.7 µm column, column Temperature: 60 °C, mobile phase: A – $H_2O$ (0.1% formic acid), B – ACN (0.1% formic acid), flow rate: 1.5 mL / min, gradient: 0.01 min – 1% B, 5.00 min – 100% B, 5.99 min – 100% B, MS Ionization mode: Electrospray ionization (ESI), MS Scan range: 83 – 1000 m/z, UV detection: 215 nm, 254 nm, 280 nm unless otherwise specified. Thin-layer chromatography (TLC) with pre-coated silica gel GF254 (0.2 mm) was used and the results were visualized using either UV light or $KMnO_4$ stain. Proton nuclear magnetic resonance ($^1$H-NMR) spectra were recorded at 400, 500 or 600 MHz on Varian or Bruker instrumentation; chemical shifts were calibrated using residual non-deuterated solvents $CHCl_3$ (δ = 7.26 ppm), DMSO (δ = 2.50 ppm) or MeOH (δ = 3.31 ppm) and expressed in *δ* ppm. Coupling constants (*J*), when given, are reported in hertz. Multiplicities are reported using the following abbreviations: s = singlet, d = doublet, dd = doublet of doublets, t = triplet, q = multiplet (range of multiplets is given), br = broad signal, dt = doublet of triplets. $^{19}$F NMR spectra were recorded at 376 MHz (Varian), $^{13}$C NMR spectra were recorded at 101, 126 or 151 MHz (Varian). $^{13}$C NMR chemical shifts for $^{13}$C NMR are reported relative to the central $CHCl_3$ (δ = 77.16 ppm), DMSO (δ = 39.52 ppm) or MeOH (δ = 49.00 ppm) and chemical shifts are reported in parts per million (ppm). All final compounds were purified by reverse phase high-performance liquid chromatography (HPLC) or supercritical fluid chromatography (SFC) or silica gel chromatography (100-200 mesh). HPLC was done with an Agilent 1260 HPLC instrument (Agilent Technologies, Germany) equipped with a G7161A Preparative Binary Pump, a G7157A Prep Autosampler, a G7115A DAD WR and a G7159B Preparative Fraction Collector. The Open Lab CDS software (version C.01.10 was used for instrument control, data acquisition and data handling). SFC was done with a Waters 100q Prep SFC System. Chiral HPLC analytical analysis was done with an Agilent 1200 HPLC instrument (Agilent Technologies, Germany) equipped with a G1379B degasser, a G1312A Binary Pump, a G1329A ALS autosampler, a G1315A Diode Array Detector. Chiral SFC analytical analysis was done with an Agilent 1260 SFC instrument (Agilent Technologies, Germany) equipped with a G1379B degasser, a G1312B Binary Pump, a G1313A ALS autosampler, a G1316A thermostatted column compartment, a G1315D Diode Array Detector and an Aurora SFC systems. Melting points were taken using OptiMelt Automated Melting Point System Digital Image Processing Technology SRS Stanford Pesearch Systems, 2 $^o$C/min (5 $^o$C/min at high melting point). Optical rotation was measured with RudolphAutopol V/ Mettler Toledo XSR205DU. Standard conditions for analysis: solution concentration 0.5 g/100 mL (solvent: MeOH), wavelength 589 nm, temperature 21 $^o$C. All oxamides exist as rotamers in $^1$H NMR spectra. All compounds are > 95% pure by HPLC.

The synthesis of TNG462, Compounds 1, *tert*-butyl (*S*)-5-methyl-2-oxopiperidine-1-carboxylate, and both the SEM and THP protected 7-bromo-1*H*-pyrazolo[4,3-*c*]pyridin-4-amine have been previously.[1]
The synthesis of Compounds 2, 10 & 11, as well as 2-((5-carbamoylpyridin-3-yl)amino)-2-oxoacetic acid used to make Compound 8, and 2-((6-((*tert*-butoxycarbonyl)amino)-5-methylpyridin-3-yl)amino)-2-oxoacetic acid used to make Compound 9 have been previously described. [2]

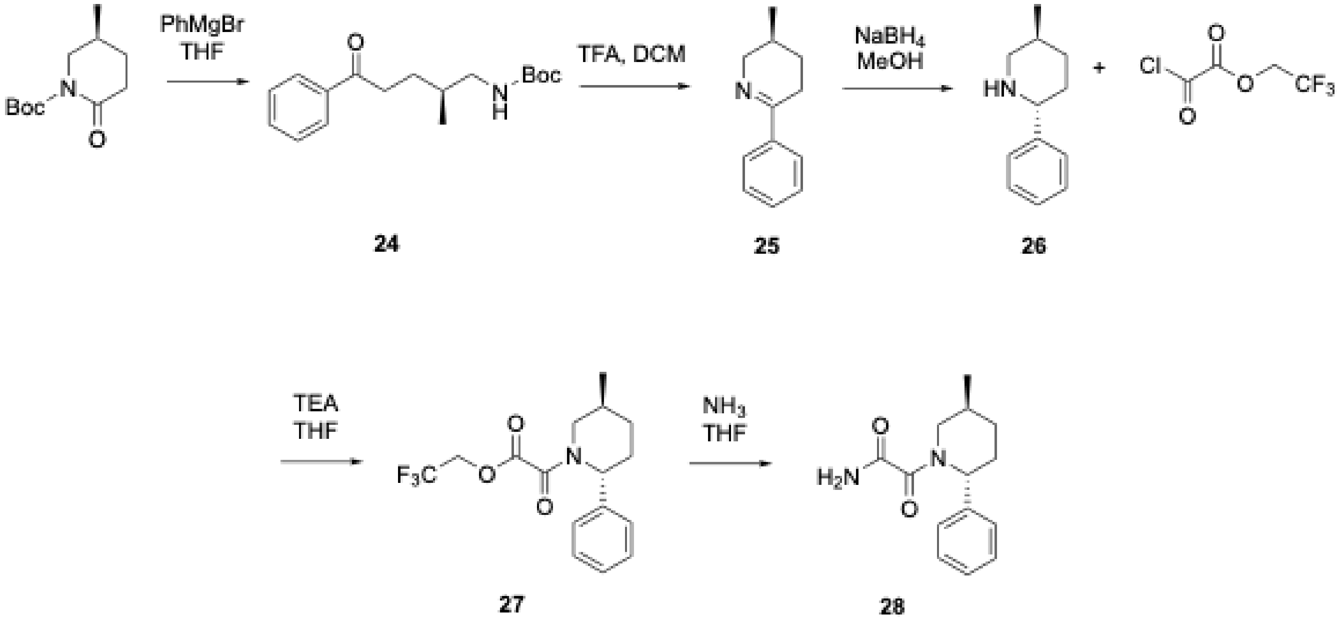


*tert*-butyl (*S*)-(2-methyl-5-oxo-5-phenylpentyl)carbamate (**24**).
To a dry 2 necked flask was added THF (150 mL) and *tert*-butyl (*5S*)-5-methyl-2-oxo-piperidine-1-carboxylate (8.00 g, 37.51 mmol) with stirring and the solution was cooled to -78 °C. Phenyl magnesium bromide (68 g, 56.27 mmol, 69 mL) reagent was added to the *Boc*-lactam over 1 hr, maintaining the internal temperature below -70 °C. The solution was warmed to room temperature and sat. $NH_4Cl$ was added. The aqueous layer was extracted 3 x 50 mL with DCM and the organic layers combined, dried over $Na_2SO_4$, filtered and concentrated in vacuum. *tert*-butyl (*S*)-(2-methyl-5-oxo-5-phenylpentyl)carbamate (**24**) (12 g, crude) was obtained as a light-yellow oil and was used in the next step without further purification. $^1$H NMR (400 MHz, $CDCl_3$) (ppm) 0.93 (d, 3H), 1.44 (s, 9H), 1.57 (m, 4H), 3.01 (m, 4H), 7.45 (m, 3H), 7.93 (d, 2H).

(*S*)-3-methyl-6-phenyl-2,3,4,5-tetrahydropyridine (**25**).
A solution of **24** (1.0 equiv) in TFA was stirred at 25 °C for 1 hr and then concentrated under vacuum. Crushed ice was added to the residue, and the resulting mixture was basified to pH 10 with 10% aqueous potassium carbonate solution and extracted with DCM (2x). The combined organic extracts were dried over sodium sulphate and concentrated under reduced pressure to afford (*S*)-3-methyl-6-phenyl-2,3,4,5-tetrahydropyridine (**25**), 84 % yield. $^1$H NMR (500 MHz, $CDCl_3$) (ppm) 0.99 (d, 3H), 1.39 (m, 1H), 1.73 (m, 1H), 1.92 (m, 1H), 2.58 (m, 1H), 2.77 (m, 1H), 3.26 (m, 1H), 3.99 (m, 1H), 7.37 (m, 3H), 7.78 (m, 2H).

(2*R*,5*S*)-5-methyl-2-phenylpiperidine (**26**).
**25** (1 eq) was dissolved in MeOH and the resulting solution was cooled to 0 °C in an ice bath. Sodium borohydride (2 eq) was added portion wise to the solution upon which the reaction mixture was allowed to warm to room temperature and stirred overnight. Water was added to the reaction mixture and the resulting mixture was concentrated under vacuum. The residue was diluted with water and extracted with DCM twice, dried over $Na_2SO_4$, filtered, and concentrated to obtain (2*R*,5*S*)-5-methyl-2-phenylpiperidine (**26**), 91 % yield. $^1$H NMR (500 MHz, $CDCl_3$) (ppm) 0.90 (d, 3H), 1.15 (m, 1H), 1.78 (m, 2H), 1.82 (m, 3H), 2.42 (m, 1H), 3.13 (m, 1H), 3.55 (m, 1H), 7.35 (m, 5H).

2,2,2-trifluoroethyl 2-((2*R*,5*S*)-5-methyl-2-phenylpiperidin-1-yl)-2-oxoacetate (**27**).
**26** (1 eq) and TEA (1.1 eq) were dissolved in THF and cooled to 0 °C followed by the dropwise addition of 2,2,2-trifluoroethyl 2-chloro-2-oxo-acetate (1.1 eq) under Ar. The reaction mixture was stirred for 12 hr at room temperature and evaporated under reduced pressure to give 2,2,2-trifluoroethyl 2-((2*R*,5*S*)-5-methyl-2-phenylpiperidin-1-yl)-2-oxoacetate (**27**) which was used in the next step without further purification, 61 % yield. LCMS (ESI): $[M]^+$ m/z: calcd 329.2; found 330.2; Rt = 2.657 min.

2-((2*R*,5*S*)-5-methyl-2-phenylpiperidin-1-yl)-2-oxoacetamide (**28**).
To a solution of **27** (1 eq) in THF, ammonia (1 eq) was bubbled through for 10 min at 0 °C. The reaction mixture was then stirred for 18 hr at room temperature. The reaction mixture was filtered and the filtrate was concentrated under vacuum to give 2-((2*R*,5*S*)-5-methyl-2-phenylpiperidin-1-yl)-2-oxoacetamide (**28**) which was used in the next step without further purification, 65 % yield. HPLC conditions: Column: YMC Triart C18 100 x 20 mm, 5 µM; 0-5 min 15-35 % water-MeCN + 0.1 % $NH_4OH$ 30 mL / min; (loading pump 4 mL / min MeCN). LCMS (ESI): $[M]^+$ m/z: calcd 246.2; found 247.2; Rt = 1.150 min.

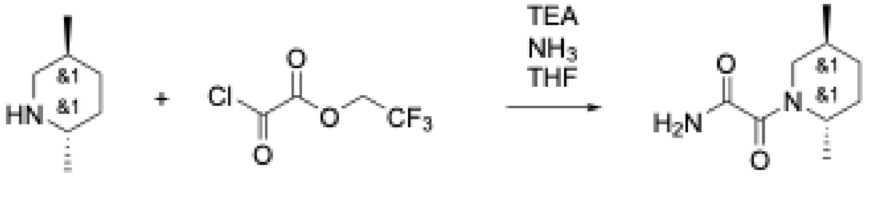


**Intermediate 7a**

*rac*-2-((2*R*,5*R*)-2,5-dimethylpiperidin-1-yl)-2-oxoacetamide, **intermediate 7a**
2,2,2-Trifluoroethyl 2-chloro-2-oxo-acetate (1.65 g, 8.69 mmol) was added slowly to a cooled to -10 °C mixture of *rac*-(*2R,5R*)-2,5-dimethylpiperidine (1 g, 6.68 mmol, HCl) and TEA (3.4 g, 33.41 mmol, 4.7 mL) in THF (40 mL) . The resulting mixture was allowed to warm to 25 °C and stirred for 2 h. Ammonia (g) was vigorously bubbled through the solution at 25 °C for 1 h. The resulting mixture was filtered and the filter cake was washed with THF (3 x 15 mL) and discarded. The combined filtrate was concentrated under vacuum to afford crude *rac*-2-((2*R*,5*R*)-2,5-dimethylpiperidin-1-yl)-2-oxoacetamide (1.1 g, 5.97 mmol, 89% yield) as a yellow solid, which was used directly in the next step. LCMS (ESI): $[M]^+$ m/z: calcd 184.2; found 185.2; Rt = 0.815 min.

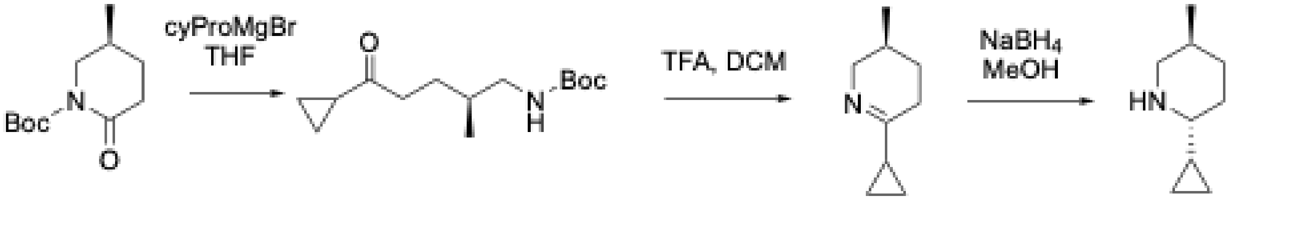


**Intermediate 5a'**

*tert*-butyl (*S*)-(5-cyclopropyl-2-methyl-5-oxopentyl)carbamate
Cyclopropylmagnesium bromide (2.4 g, 16.9 mmol, 1.4 mL) was transferred dropwise to a cold (-78 °C) suspension of *tert*-butyl (*S*)-5-methyl-2-oxopiperidine-1-carboxylate (3 g, 14.1 mmol) in THF (50 mL). The reaction mixture was stirred at -78 °C for 1 h before being warmed to room temperature and stirred for 12 h. The reaction was diluted with MTBE (50 mL) and slowly quenched with 50 mL of saturated ammonium chloride aqueous solution. The organic phase was washed with a saturated aqueous solution of sodium bicarbonate (2 x 50 mL). The combined aqueous fractions were extracted two times with MTBE. The combined organic fractions were dried over sodium sulfate and concentrated to obtain crude *tert*-butyl (*S*)-(5-cyclopropyl-2-methyl-5-oxopentyl)carbamate that was used in the next step without further purification, (3.6 g, crude).
$^1$H NMR (500 MHz, $CDCl_3$) (ppm) 0.88 (m, 5H), 0.99 (m, 2H), 1.43 (m, 11H), 1.60 (m, 1H), 1.90 (m, 1H), 2.57 (m, 2H), 2.99 (m, 2H), 4.67 (m, 1H).

(*S*)-6-cyclopropyl-3-methyl-2,3,4,5-tetrahydropyridine
*tert*-butyl (*S*)-(5-cyclopropyl-2-methyl-5-oxopentyl)carbamate (3.4 g, 13.31 mmol) was dissolved in TFA (39.94 mmol, 3.08 mL) and the resulting reaction mixture was stirred for 1 h, upon which 50% aq. NaOH solution was added to pH = 13 - 14. The resulting mixture was extracted with DCM (4 x 100 mL). The combined organic phase was dried over $MgSO_4$ and concentrated under reduced pressure. (*S*)-6-cyclopropyl-3-methyl-2,3,4,5-tetrahydropyridine (1.72 g, 12.53 mmol, 94.14% yield) was obtained as brown oil and used as is. $^1$H NMR (500 MHz, $CDCl_3$) (ppm) 0.67 (m, 3H), 0.89 (d, 3H), 1.19 (m, 1H), 1.48 (m, 1H), 1.71 (m, 2H), 2.14 (m, 2H), 2.93 (m, 1H), 3.65 (m, 1H).

(2*R*,5*S*)-2-cyclopropyl-5-methylpiperidine, **intermediate 5a'**
To an ice-cold solution of (*S*)-6-cyclopropyl-3-methyl-2,3,4,5-tetrahydropyridine (1.72 g, 12.53 mmol) in MeOH (49.56 mL) was added sodium borohydride (474.21 mg, 12.53 mmol) in one portion and the resulting mixture was stirred for 5 min. The reaction mixture was concentrated and then partitioned in a mixture of DCM/water. The water was further extracted with DCM (50 mL) and the combined organics were dried over $Na_2SO_4$ and evaporated to give (2*R*,5*S*)-2-cyclopropyl-5-methylpiperidine, **intermediate 5a'** (1.7 g, crude) as a brown liquid that was used in the next step without further purification (and contained about 10% cis material).

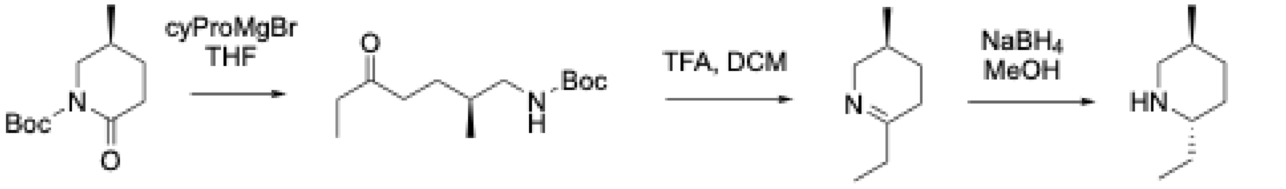


**Intermediate 6a'**

*tert*-butyl (*S*)-(2-methyl-5-oxoheptyl)carbamate

Ethylmagnesium bromide (16.88 mmol, 1.29 mL) was transferred dropwise to a cold (-78 °C ) suspension of *tert*-butyl (*S*)-5-methyl-2-oxopiperidine-1-carboxylate (3 g, 14.07 mmol) in THF (199 mL).The reaction mixture was stirred at -78 °C for 10 min before being warmed to room temperature and stirred for 12 h. The reaction was diluted with MTBE (300 mL) and quenched with 100 mL of saturated ammonium chloride aqueous solution. The organic phase was washed with a saturated aqueous solution of sodium bicarbonate (2 x 200 mL). The combined aqueous fractions were back-extracted two times with MTBE. The combined organic fractions were dried over sodium sulfate and concentrated by rotary evaporation to obtain *tert*-butyl (*S*)-(2-methyl-5-oxoheptyl)carbamate (2.8 g, 11.51 mmol, 82 % yield) as a pale-yellow gum. $^1$H NMR (400 MHz, $CDCl_3$) (ppm) 0.87 (m, 3H), 1.02 (m, 3H), 1.41 (m, 10H), 1.61 (m, 2H), 2.41 (m, 4H), 2.97 (m, 2H), 4.62 (m, 1H).

(*S*)-6-ethyl-3-methyl-2,3,4,5-tetrahydropyridine

*tert*-butyl (*S*)-(2-methyl-5-oxoheptyl)carbamate (2.80 g, 11.51 mmol) was dissolved in TFA (2.7 mL) and the resulting reaction mixture was stirred for 1 h. After which 50% aq. NaOH solution was added to pH = 13 - 14. The resulting mixture was extracted with DCM (4 x 100 mL) and the combined organic phase was dried over $MgSO_4$ and concentrated under reduced pressure to afford (*S*)-6-ethyl-3-methyl-2,3,4,5-tetrahydropyridine (1.5 g, crude) as light-yellow oil which was used as is.

(2*S*,5*S*)-2-ethyl-5-methylpiperidine, **intermediate 6a'**

To an ice cold solution of (*S*)-6-ethyl-3-methyl-2,3,4,5-tetrahydropyridine (1.5 g, 11.98 mmol) in MeOH (15 mL) was added sodium borohydride (453.23 mg, 11.98 mmol) in one portion and the resulting mixture was stirred overnight at room temperature. The reaction mixture was concentrated and partitioned in a mixture EtOAc/water. The water was further extracted with EtOAc (10 mL) and the combined organics were dried over $Na_2SO_4$ and evaporated to give (2*S*,5*S*)-2-ethyl-5-methylpiperidine, **intermediate 6a'** (1.5 g, 11.79 mmol, 98% yield) as a light-yellow oil.

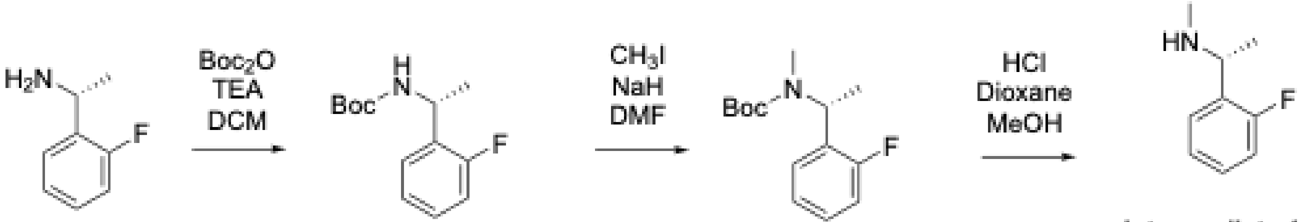


**intermediate 14a'**

*tert*-butyl (*R*)-(1-(2-fluorophenyl)ethyl)carbamate

To a solution of (*R*)-1-(2-fluorophenyl)ethanamine (0.7 g, 3.99 mmol, HCl) and triethylamine (605 mg, 5.98 mmol, 833 µL) in DCM (20 mL), di-*tert*-butyl dicarbonate (826 mg, 3.79 mmol) was added. The resulting mixture was stirred at 25 °C for 3 h, washed with water (3 × 20 mL), dried over anhydrous sodium sulfate and concentrated under vacuum to give *tert*-butyl (*R*)-(1-(2-fluorophenyl)ethyl)carbamate (0.92 g, 3.84 mmol, 96% yield) as a light-yellow oil. LCMS (ESI): $[M\text{-}t\text{-}Bu]^-$ m/z: calcd 184.1; found 184.1; Rt = 1.492 min.

*tert*-butyl (*R*)-(1-(2-fluorophenyl)ethyl)(methyl)carbamate

To a stirred at 0°C solution of *tert*-butyl (*R*)-(1-(2-fluorophenyl)ethyl)carbamate (920 mg, 3.84 mmol) in DMF (15 mL), sodium hydride (60 % dispersion in mineral oil, 308 mg, 7.69 mmol) was added. After 30 min, methyl iodide (7.69 mmol, 479 µL) was added. The resulting mixture was stirred at 25 °C for 6 h, poured into water (80 mL), extracted with MTBE (3 × 15 mL), dried over anhydrous sodium sulfate, and concentrated under vacuum to obtain *tert*-butyl (*R*)-(1-(2-fluorophenyl)ethyl)(methyl)carbamate (0.9 g, 3.55 mmol, 92% yield) as a light-yellow oil. LCMS (ESI): [M-t-Bu]- m/z: calcd 198.2; found 198.2; Rt = 1.579 min.

(*R*)-1-(2-fluorophenyl)-*N*-methylethan-1-amine, **intermediate 14a'**

A solution of *tert*-butyl (*R*)-(1-(2-fluorophenyl)ethyl)(methyl)carbamate (900 mg, 3.55 mmol) in MeOH (20 mL) and 4.0 M hydrogen chloride solution in dioxane (219.41 mmol, 10 mL) was stirred at 25 °C for 3 h. The solvent was evaporated to give (*R*)-1-(2-fluorophenyl)-*N*-methylethan-1-amine, **intermediate 14a'** (0.65 g, 3.43 mmol, 96% yield, HCl) as a light-yellow solid. LCMS (ESI): $[M+H]^+$ m/z: calcd 154.11; found 154.0; Rt = 0.501 min.

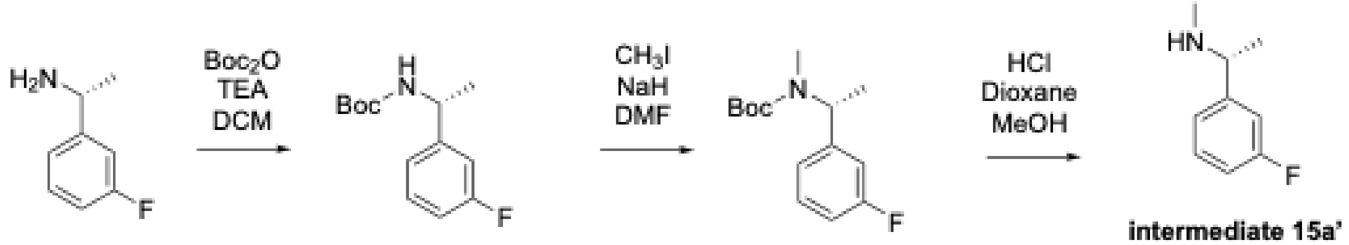


**intermediate 15a'**

*tert*-butyl (*R*)-(1-(3-fluorophenyl)ethyl)carbamate

To a solution of (*R*)-1-(3-fluorophenyl)ethanamine (0.8 g, 4.56 mmol, HCl) and triethylamine (6.83 mmol, 952 µL) in DCM (20 mL), di-tert-butyl dicarbonate (944.43 mg, 4.33 mmol) was added. The resulting mixture was stirred at 25 °C for 3 h, washed with water (3 × 20 mL), dried over anhydrous sodium sulfate, and concentrated under vacuum to give *tert*-butyl (*R*)-(1-(3-fluorophenyl)ethyl)carbamate (1.05 g, 4.39 mmol, 96% yield) as a light-yellow oil. LCMS (ESI): $[M-H]^-$ m/z: calcd 238.12; found 238.1; Rt = 1.402 min.

*tert*-butyl (*R*)-(1-(3-fluorophenyl)ethyl)(methyl)carbamate

To a stirred at 0°C solution of *tert*-butyl (*R*)-(1-(3-fluorophenyl)ethyl)carbamate (1.05g, 4.39 mmol) in DMF (15 mL), sodium hydride (60 % dispersion in mineral oil, 351 mg, 8.78 mmol) was added. After 30 min, methyl iodide (8.78 mmol, 546 µL) was added. The resulting mixture was stirred at 25 °C for 6 h, poured into water (80 mL), extracted with MTBE (3 × 15 mL), dried over anhydrous sodium sulfate, and concentrated under vacuum to obtain *tert*-butyl (*R*)-(1-(3-fluorophenyl)ethyl)(methyl)carbamate (1 g, 3.95 mmol, 90% yield) as a light-yellow oil. LCMS (ESI): [M-t-Bu]- m/z: calcd 198.0; found 198.0; Rt = 1.594 min.

(*R*)-1-(3-fluorophenyl)-*N*-methylethan-1-amine, **intermediate 15a'**

A solution of *tert*-butyl (*R*)-(1-(3-fluorophenyl)ethyl)(methyl)carbamate (1 g, 3.95 mmol) in MeOH (20 mL) and 4.0 M hydrogen chloride solution in dioxane (219.41 mmol, 10 mL) was stirred at 25 °C for 3 h. The solvent was evaporated to give (*R*)-1-(3-fluorophenyl)-*N*-methylethan-1-amine, **intermediate 15a'** (0.74 g, 3.90 mmol, 99% yield, HCl) as a light-yellow solid. LCMS (ESI): $[M+H]^+$ m/z: calcd 154.11; found 154.0; Rt = 0.518 min.

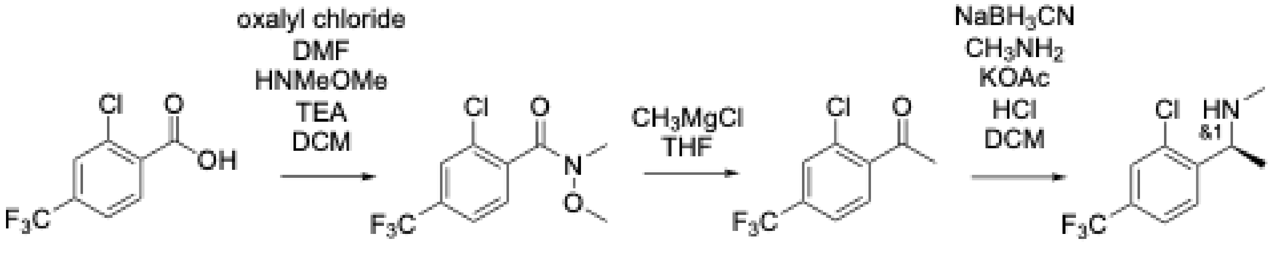


**intermediate 19a'**

2-chloro-*N*-methoxy-*N*-methyl-4-(trifluoromethyl)benzamide

Oxalyl chloride (13.36 mmol, 1.17 mL) was added to the solution of 2-chloro-4-(trifluoromethyl)benzoic acid (2 g, 8.91 mmol) in dichloromethane (30 mL) followed by dimethylformamide (445.31 µmol, 34.48 µL). The mixture was stirred at 25 °C for 2.5 h. After gas evolution ceased and the solution became clear, the volatiles were removed under reduced pressure and residue was dissolved in dichloromethane (10 mL). The solution was added dropwise to a suspension of *N,O*-dimethylhydroxylamine hydrochloride (1.13 g, 11.58 mmol) and triethylamine (26.72 mmol, 3.72 mL) in dichloromethane (40 mL). The reaction mixture was stirred at 25 °C for 4 h. 20 % aqueaous $K_2CO_3$ solution (30 mL) was added and the mixture was stirred for 10 minutes. The organic layer was separated, dried over solid $K_2CO_3$, and concentrated under reduced pressure, leaving 2-chloro-*N*-methoxy-*N*-methyl-4-(trifluoromethyl)benzamide (2.28 g, 8.52 mmol, 96% yield). LCMS (ESI): $[M+H]^+$ m/z: calcd 268.2; found 268.0; Rt = 1.176 min.

1-(2-chloro-4-(trifluoromethyl)phenyl)ethan-1-one

3 M methylmagnesium chloride in THF (12.78 mmol, 4.30 mL) was added dropwise to the solution of 2-chloro-*N*-methoxy-*N*-methyl-4-(trifluoromethyl)benzamide (2.28 g, 8.52 mmol) in tetrahydrofuran (40 mL). The resulting reaction mixture was stirred at 20 °C for 5 h, then it was quenched with $NH_4Cl$ (30 mL, saturated aqueous solution) and stirred for 10 minutes. The organic layer was separated, dried over solid $K_2CO_3$, and concentrated under reduced pressure to afford 1-(2-chloro-4-(trifluoromethyl)phenyl)ethan-1-one (1.8 g, 8.09 mmol, 95% yield). $^1H$ NMR (500 MHz, $CDCl_3$) (ppm) 2.65 (s, 3H), 7.58 – 7.60 (m, 2H), 7.68 (s, 1H).

*rac*-(*R*)-1-(2-chloro-4-(trifluoromethyl)phenyl)-*N*-methylethan-1-amine, **intermediate 19a'**

To the stirred suspension of methylamine hydrochloride (5.46 g, 80.87 mmol) and potassium acetate (7.94 g, 80.87 mmol) in methanol (34.16 mL) was added 1-(2-chloro-4-(trifluoromethyl)phenyl)ethan-1-one (1.8 g, 8.09 mmol) followed by sodium cyanoborohydride (762 mg, 12.13 mmol). The resulting reaction mixture was stirred at 25 °C for 20 h, then the solvent was removed under reduced pressure and the residue was partitioned between MTBE (40 mL) and $K_2CO_3$ (20% aqueous solution, 50 mL). The organic layer was separated, dried over solid $K_2CO_3$, and filtered. The filtrate was acidified with 4 M hydrogen chloride solution in dioxane (16.17 mmol, 5.84 mL) and the resulting solution was left for 2 h to allow crystallization. The resulting crystalline precipitate was filtered and dried, affording *rac*-(*R*)-1-(2-chloro-4-(trifluoromethyl)phenyl)-*N*-

methylethan-1-amine, **intermediate 19a'** (1.07 g, 3.90 mmol, 48% yield, HCl). $^1$H NMR (500 MHz, $CDCl_3$) (ppm) 1.59 (d, 3H), 4.71 (q, 1H), 7.90 (d, 1H), 8.00 (s, 1H), 8.16 (d, 1H), 9.75 (br s, 1H), 10.17 (br s, 1H).

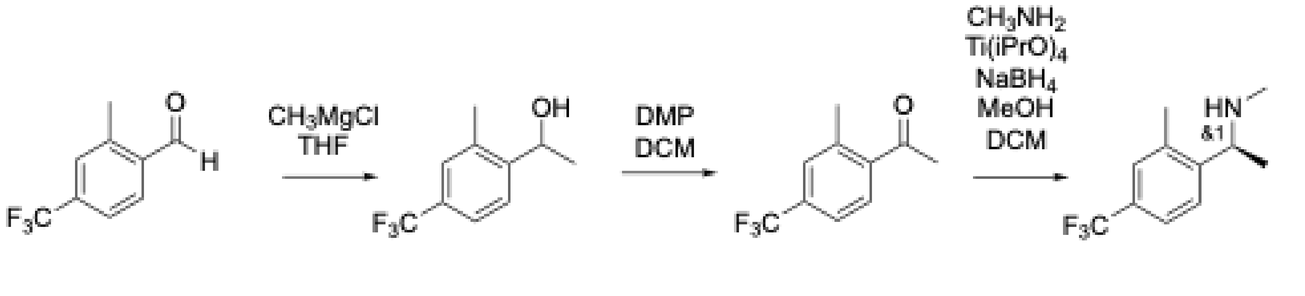


**intermediate 20a'**

1-(2-methyl-4-(trifluoromethyl)phenyl)ethan-1-ol

Methyl magnesium chloride (1.94 g, 25.91 mmol) was added dropwise to a stirred solution of 2-methyl-4-(trifluoromethyl)benzaldehyde (3.25 g, 17.27 mmol) in THF (60 mL) at 0 °C, then stirred and slowly allowed to warm to 20 °C for 2 hr. Water (5 mL) was added and the mixture was stirred for 10 min and filtered. The filtrate was concentrated under vacuum to give 1-(2-methyl-4-(trifluoromethyl)phenyl)ethan-1-ol (3.3 g, 16.16 mmol, 94% yield) as a white solid.

1-(2-methyl-4-(trifluoromethyl)phenyl)ethan-1-one

To a solution of 1-(2-methyl-4-(trifluoromethyl)phenyl)ethan-1-ol (3.3 g, 16.16 mmol) in DCM (75 mL) was added Dess-Martin periodinane (8.23 g, 19.39 mmol) in one portion. The resulting mixture was stirred 2 h at room temperature after which it was concentrated and the residue was triturated with MTBE (200 mL) and filtered. The filtrate was washed with saturated $NaHCO_3$ solution (20 mL), dried over anhydrous sodium sulfate, and concentrated under vacuum to give 1-(2-methyl-4-(trifluoromethyl)phenyl)ethan-1-one (1.5 g, 7.42 mmol, 46% yield) as a light-yellow solid.

*rac*-(*R*)-1-(2-chloro-4-(trifluoromethyl)phenyl)-*N*-methylethan-1-amine, **intermediate 20a'**

A mixture of 1-(2-methyl-4-(trifluoromethyl)phenyl)ethan-1-one (0.5 g, 2.47 mmol), titanium isopropoxide (2.97 mmol, 883.21 µL), methanamine (8.06 mmol, 2.79 mL) in DCM (14.72 mL) was stirred under Ar at ambient temperature for 8 h. Sodium borohydride (93.56 mg, 2.47 mmol) was added and the resulting mixture was stirred for an additional 1 h at ambient temperature. Sodium hydroxide (5 mL, 20%) was added and the reaction mixture was stirred for 10 min and filtered. The DCM layer was separated and dried over anhydrous sodium sulfate, then concentrated under vacuum to give crude *rac*-(*R*)-1-(2-chloro-4-(trifluoromethyl)phenyl)-*N*-methylethan-1-amine, **intermediate 20a'** (0.4 g, 1.84 mmol, 74% yield) as a light-yellow oil. LCMS (ESI): $[M+H]^+$ m/z: calcd 218.12; found 218.0; Rt = 0.956 min.

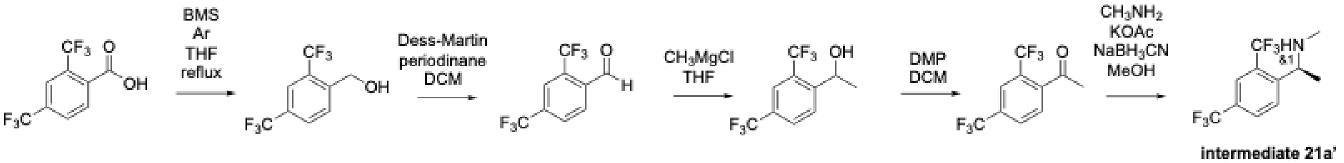


**intermediate 21a'**

(2,4-bis(trifluoromethyl)phenyl)methanol

To a solution of 2,4-bis(trifluoromethyl)benzoic acid (10 g, 38.74 mmol) in THF (250 mL) was added borane dimethyl sulfide complex (12.42 g, 163.43 mmol, 15.5 mL) under Ar atmosphere. After stirring at reflux for 18 h, the resulting mixture was quenched with MeOH, refluxed for 1h, evaporated to dryness. MeOH was added and the mixture was concentrated to afford (2,4-bis(trifluoromethyl)phenyl)methanol (9.95 g, crude) as a light-yellow gum. $^1$H NMR (500 MHz, $CDCl_3$) (ppm) 2.03 (s, 1H), 4.96 (s, 2H), 7.83 – 7.94 (m, 3H).

2,4-bis(trifluoromethyl)benzaldehyde

To a solution of (2,4-bis(trifluoromethyl)phenyl)methanol (9.95 g, 36.68 mmol) in DCM (250 mL) was added Dess-Martin periodinane (18.67 g, 44.02 mmol) in one portion. The resulting mixture was stirred for 1 h at room temperature. The reaction mixture was poured into a solution containing $Na_2S_2O_3$ and $Na_2CO_3$ (2:1 by weight), stirred for 30 min, and concentrated to afford 2,4-bis(trifluoromethyl)benzaldehyde (8.5 g, 35.11 mmol, 96% yield) as a yellow oil. $^1$H NMR (500 MHz, $CDCl_3$) (ppm) 7.99 (d, 1H), 8.04 (s, 1H), 8.26 (d, 1H), 10.44 (s, 1H).

1-(2,4-bis(trifluoromethyl)phenyl)ethan-1-ol

Methyl magnesium bromide (6.28 g, 52.66 mmol, 2.1 mL) was added to a solution of 2,4-bis(trifluoromethyl)benzaldehyde (35.11 mmol, 5.74 mL) in THF (250 mL) maintaining the internal temperature below 25 °C. The resulting reaction mixture was allowed to stir at room temperature for 12 h, then quenched with saturated $NH_4Cl$ solution and extracted with EtOAc (2 x 125 mL). The combined organic layer was dried over $Na_2SO_4$, filtered, and concentrated under reduced pressure to obtain product 1-(2,4-bis(trifluoromethyl)phenyl)ethan-1-ol (8.5 g, 32.93 mmol, 94% yield) as a light-yellow oil, which was used in the next step reaction without any further purification. $^1$H NMR (500 MHz, $CDCl_3$) (ppm) 1.48 (d, 1H), 2.14 (br s, 1H), 5.35 (q, 1H), 7.81 (d, 1H), 7.84 (s, 1H), 7.99 (d, 1H).

1-(2,4-bis(trifluoromethyl)phenyl)ethan-1-one

To a solution of 1-(2,4-bis(trifluoromethyl)phenyl)ethan-1-ol (8.5 g, 32.93 mmol) in DCM (125 mL) was added Dess-Martin periodinane (16.76 g, 39.51 mmol) in one portion. The resulting mixture was stirred for 1 h at room temperature. The reaction mixture was poured into a solution containing $Na_2S_2O_3$ and $Na_2CO_3$ (2:1 by weight), stirred for 30min, and concentrated to afford 1-(2,4-bis(trifluoromethyl)phenyl)ethan-1-one (8 g, 31.23 mmol, 95% yield) as a light-yellow oil. $^1H$ NMR (400 MHz, $CDCl_3$) (ppm) 2.57 (s, 3H), 7.55 (d, 1H), 7.86 (d, 1H), 7.93 (s, 1H).

*rac*-(*R*)-1-(2,4-bis(trifluoromethyl)phenyl)-*N*-methylethan-1-amine, **intermediate 21a'**
To a solution of 1-(2,4-bis(trifluoromethyl)phenyl)ethan-1-one (1 g, 3.90 mmol) in MeOH (50 mL) was added sodium cyanoborohydride (368.00 mg, 5.86 mmol), potassium acetate (3.83 g, 39.04 mmol) and N-methylamine (2.64 g, 39.04 mmol, HCl). The mixture was refluxed for 96 h. The reaction was then quenched with water (100 mL) and extracted with EtOAc (100 mL x 2). The combined organic layers were washed with brine (2 x 50 mL), dried over anhydrous $Na_2SO_4$ and concentrated under reduced pressure to afford *rac*-(*R*)-1-(2,4-bis(trifluoromethyl)phenyl)-*N*-methylethan-1-amine, **intermediate 21a'** (0.2 g, 737.46 µmol, 19% yield) as a yellow oil. LCMS (ESI): $[M+H]^+$ m/z: calcd 272.2; found 272.2; Rt = 1.996 min.

# General Procedure A

Intermediates 4a, 5a, 6a, 12a, 13a, 14a, 15a, 16a, 17a, 18a, 19a, 20a, 21a, 22a

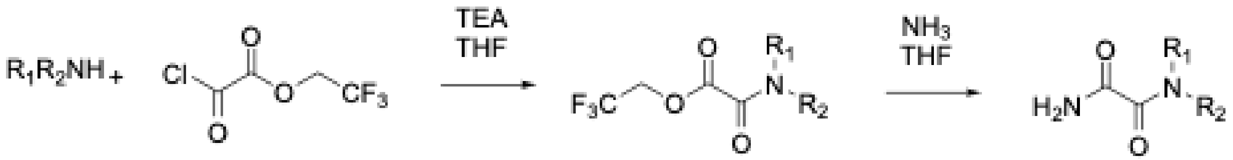


Amine (1eq) was dissolved in DCM (0.2 M) and TEA (2.5 eq or 3.5 eq if amine is a salt) was added. Then reaction mixture was cooled and 2,2,2-trifluoroethyl 2-chloro-2-oxo-acetate (1 eq) was added dropwise. The reaction was stirred at room temperature overnight. Water was added and the organic layer was washed with brine, dried over $Na_2SO_4$, and concentrated under vacuum to afford oxamic ester which was dissolved in MeOH / $NH_3$ (0.2 M) and stirred overnight at room temperature. The solution was concentrated under vacuum to afford oxamide.

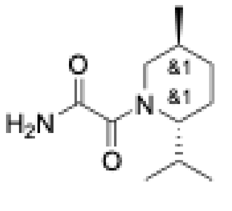

**Intermediate 4a**

*rac*-2-((2*R*,5*S*)-2-isopropyl-5-methylpiperidin-1-yl)-2-oxoacetamide, **intermediate 4a**
amine: *rac*-(*2S,5R*)-2-*iso*propyl-5-methyl-piperidine
ester: 50% yield. LCMS (ESI): $[M+1]^+$ m/z: calcd 295.2; found 296.2; Rt = 1.207 min.
oxamide: *rac*-2-((2*R*,5*S*)-2-isopropyl-5-methylpiperidin-1-yl)-2-oxoacetamide, **intermediate 4a**, 84 % yield. LCMS (ESI): $[M+1]^+$ m/z: calcd 212.2; found 213.2; Rt = 1.086 min.

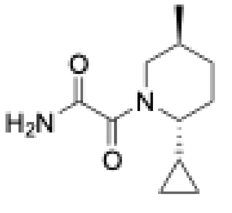

**Intermediate 5a**

2-((2*R*,5*S*)-2-cyclopropyl-5-methylpiperidin-1-yl)-2-oxoacetamide, **intermediate 5a**
amine: intermediate 5a'
ester: used as is in the next step

oxamide: 2-((2*R*,5*S*)-2-cyclopropyl-5-methylpiperidin-1-yl)-2-oxoacetamide, **intermediate 5a**, LCMS (ESI): $[M+H]^+$ m/z: calcd 211.4; found 211.4; Rt = 2.444 min. Used as is in the next step.

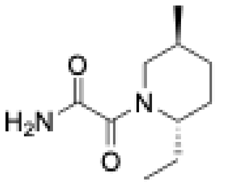

**Intermediate 6a**

2-((2*S*,5*S*)-2-ethyl-5-methylpiperidin-1-yl)-2-oxoacetamide, **intermediate 6a**
amine: intermediate 6a'
ester: 91%, LCMS (ESI): [M+H]+ m/z: calcd 282.2; found 282.2; Rt = 3.963 min., used as is in the next step
oxamide: 2-((2*S*,5*S*)-2-ethyl-5-methylpiperidin-1-yl)-2-oxoacetamide, **intermediate 6a**, LCMS (ESI): [M+H]+ m/z: calcd 199.4; found 199.4; Rt = 2.213 min. Used as is in the next step.

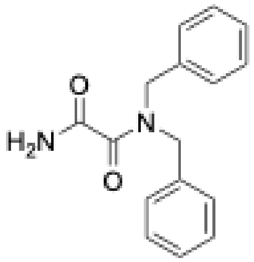

**Intermediate 12a**

$N^1$,$N^1$-dibenzyloxalamide, **intermediate 12a**
Amine: *N*-benzyl-1-phenyl-methanamine
Ester: 2,2,2-trifluoroethyl 2-(dibenzylamino)-2-oxoacetate. 60% yield. LCMS (ESI): $[M +H]^+$ m/z: calcd 352.3; found 352.2; Rt = 1.563 min.
Oxamide: $N^1$,$N^1$-dibenzyloxalamide, **intermediate 12a.** Used crude in next step. LCMS (ESI): $[M+H]^+$ m/z: calcd 270.1; found 270.0; Rt = 0.747 min

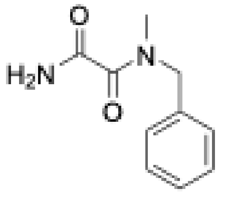

**intermediate 13a**

$N^1$-benzyl-$N^1$-methyloxalamide, **intermediate 13a**
Amine: *N*-methyl-1-phenylmethanamine
Ester: 2,2,2-trifluoroethyl 2-(benzyl(methyl)amino)-2-oxoacetate. Used crude in next step. LCMS (ESI): $[M+H]^+$ m/z: calcd 276.09; found 276.0; Rt = 1.282 min.
Oxamide: $N^1$-benzyl-$N^1$-methyloxalamide, **intermediate 13a.** 79% yield, 2 steps. LCMS (ESI): $[M+H]^+$ m/z: calcd 193.1; found 192.0; Rt = 0.742 min.

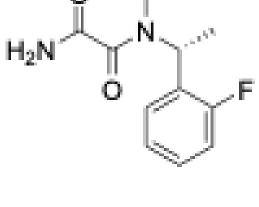

**Intermediate 14a**

(*R*)-$N^1$-(1-(2-fluorophenyl)ethyl)-$N^1$-methyloxalamide, **intermediate 14a**

Amine: (*R*)-1-(2-fluorophenyl)-*N*-methylethan-1-amine, **intermediate 14a'**
Ester: 2,2,2-trifluoroethyl (*R*)-2-((1-(2-fluorophenyl)ethyl)(methyl)amino)-2-oxoacetate. Used crude in the next step. LCMS (ESI): $[M+H]^+$ m/z: calcd 308.09; found 308.0; Rt = 1.281 min.
Oxamide: (*R*)-$N^1$-(1-(2-fluorophenyl)ethyl)-$N^1$-methyloxalamide, **intermediate 14a**. 52% yield as a light yellow solid. LCMS (ESI): $[M-H]^-$ m/z: calcd 223.09; found 223.8; Rt = 1.142 min.

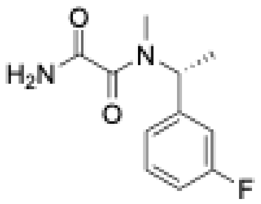

**Intermediate 15a**

(*R*)-$N^1$-(1-(3-fluorophenyl)ethyl)-$N^1$-methyloxalamide, **intermediate 15a**
Amine: (*R*)-1-(3-fluorophenyl)-*N*-methylethan-1-amine, **intermediate 15a'**
Ester: 2,2,2-trifluoroethyl (*R*)-2-((1-(3-fluorophenyl)ethyl)(methyl)amino)-2-oxoacetate. Used crude in the next step. LCMS (ESI): $[M+H]^+$ m/z: calcd 308.09; found 308.0; Rt = 1.303 min.
Oxamide: (*R*)-$N^1$-(1-(3-fluorophenyl)ethyl)-$N^1$-methyloxalamide, **intermediate 15a**. 69 % yield as a light yellow solid. LCMS (ESI): $[M+H]^+$ m/z: calcd 225.11; found 225.4; Rt = 1.301 min.

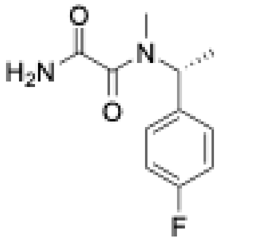

**Intermediate 16a**

(*R*)-$N^1$-(1-(4-fluorophenyl)ethyl)-$N^1$-methyloxalamide, **intermediate 16a**
Amine: (*R*)-1-(4-fluorophenyl)-*N*-methylethan-1-amine
Ester: 2,2,2-trifluoroethyl (*R*)-2-((1-(4-fluorophenyl)ethyl)(methyl)amino)-2-oxoacetate. 65 % yield as a light yellow solid.
Oxamide: (*R*)-$N^1$-(1-(4-fluorophenyl)ethyl)-$N^1$-methyloxalamide, **intermediate 16a**. 42% yield as a light yellow solid

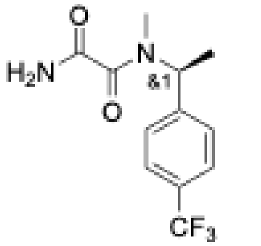

**Intermediate 17a**

*rac*-(*R*)-$N^1$-methyl-$N^1$-(1-(4-(trifluoromethyl)phenyl)ethyl)oxalamide, **intermediate 17a**
Amine: *rac*-(*R*)-*N*-methyl-1-(4-(trifluoromethyl)phenyl)ethan-1-amine
Ester: *rac*-(*R*)-$N^1$-(1-(2-fluoro-4-(trifluoromethyl)phenyl)ethyl)-$N^1$-methyloxalamide. 92 % yield as a light yellow solid. LCMS (ESI): $[M+H]^+$ m/z: calcd 358.09; found 358.2; Rt = 1.344 min.
Oxamide: *rac*-(*R*)-$N^1$-methyl-$N^1$-(1-(4-(trifluoromethyl)phenyl)ethyl)oxalamide, **intermediate 17a.** Used crude as is in the next step. LCMS (ESI): $[M-H]^-$ m/z: calcd 273.08; found 273.0; Rt = 1.224 min.

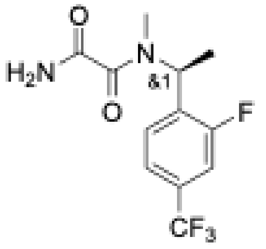

**Intermediate 18a**

*rac*-(*R*)-*N*[1]-(1-(2-fluoro-4-(trifluoromethyl)phenyl)ethyl)-*N*[1]-methyloxalamide, **intermediates 18a**
Amine: *rac*-(*R*)-1-(2-fluoro-4-(trifluoromethyl)phenyl)-*N*-methylethan-1-amine
Ester: *rac*-2,2,2-trifluoroethyl (*R*)-2-((1-(2-fluoro-4-(trifluoromethyl)phenyl)ethyl)(methyl)amino)-2-oxoacetate.
Oxamide: *rac*-(*R*)-*N*[1]-(1-(2-fluoro-4-(trifluoromethyl)phenyl)ethyl)-*N*[1]-methyloxalamide, **intermediates 18a**. Used crude as is in the next step. 68% yield.

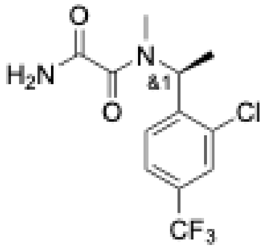

**intermediate 19a**

*rac*-(*R*)-*N*[1]-(1-(2-chloro-4-(trifluoromethyl)phenyl)ethyl)-*N*[1]-methyloxalamide, **intermediate 19a**
Amine: *rac*-(*R*)-1-(2-chloro-4-(trifluoromethyl)phenyl)-*N*-methylethan-1-amine, **intermediate 19a'**
Ester: *rac*-2,2,2-trifluoroethyl (*R*)-2-((1-(2-chloro-4-(trifluoromethyl)phenyl)ethyl)(methyl)amino)-2-oxoacetate. Crude used as is in the next step. LCMS (ESI): $[M+H]^+$ m/z: calcd 392.2; found 392.0; Rt = 1.284 min.
Oxamide: *rac*-(*R*)-*N*[1]-(1-(2-chloro-4-(trifluoromethyl)phenyl)ethyl)-*N*[1]-methyloxalamide, **intermediate 19a.** Crude used as is in the next step.

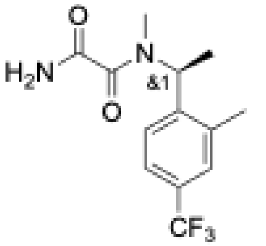

**intermediate 20a**

*rac*-(*R*)-*N*[1]-methyl-*N*[1]-(1-(2-methyl-4-(trifluoromethyl)phenyl)ethyl)oxalamide, **intermediate 20a**
Amine: *rac*-(*R*)-1-(2-chloro-4-(trifluoromethyl)phenyl)-*N*-methylethan-1-amine, **intermediate 20a'**
Ester: *rac*-2,2,2-trifluoroethyl (*R*)-2-(methyl(1-(2-methyl-4-(trifluoromethyl)phenyl)ethyl)amino)-2-oxoacetate. Crude used as is in the next step.
Oxamide: *rac*-(*R*)-*N*[1]-methyl-*N*[1]-(1-(2-methyl-4-(trifluoromethyl)phenyl)ethyl)oxalamide, **intermediate 20a**. Crude used as is in the next step.

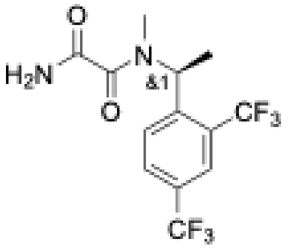


*rac*-(*R*)-*N*[1]-(1-(2,4-bis(trifluoromethyl)phenyl)ethyl)-*N*[1]-methyloxalamide, **intermediate 21a.**
Amine: *rac*-(*R*)-1-(2,4-bis(trifluoromethyl)phenyl)-*N*-methylethan-1-amine, **intermediate 21a'**
Ester: *rac*-methyl (*R*)-2-((1-(2,4-bis(trifluoromethyl)phenyl)ethyl)(methyl)amino)-2-oxoacetate. 76 % yield. Crude used as is in the next step. LCMS (ESI): $[M+H]^+$ m/z: calcd 358.2; found 358.2; Rt = 3.422 min.
Oxamide: *rac*-(*R*)-*N*[1]-(1-(2,4-bis(trifluoromethyl)phenyl)ethyl)-*N*[1]-methyloxalamide, **intermediate 21a.** 25 % yield. Yellow solid. LCMS (ESI): $[M-H]^+$ m/z: calcd 341.2; found 341.2; Rt = 2.936 min.

# General Procedure B

Compounds 3-7, 12-23, and **TNG456**

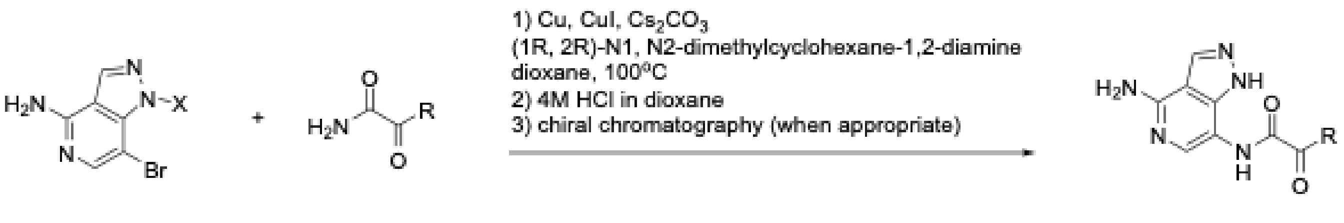


Oxamide starting material (1 eq), protected 7-bromo-1*H*-pyrazolo[4,3-*c*]pyridin-4-amine (1.5 eq), Cu (0.1 eq), CuI (0.3 eq), cesium carbonate (1.5 eq) and (1*R*,2*R*)-*N1*,*N2*-dimethylcyclohexane-1,2-diamine (0.3 eq) were mixed in dioxane (0.2 M), purged with Ar for 2 minutes and then heated in a sealed tube at 100 °C for 18 h. The mixture was filtered and the filtrate was evaporated in vacuo to afford desired product.

*copper was prepared by the following procedure: Zn powder 2 eq was added slowly under intensive stirring to 10 % solution of $Cu(NO_3)_2$ (10 eq) in water. The mixture was stirred for 90 min, then the resulting solid was filtered, washed with 10 % solution of $Cu(NO_3)_2$, and distilled water. The solids were dried for 5 h at 80 °C to give black powder of copper
**1,4-dioxane was dried over molecular sieves
***the insoluble materials must be removed with centrifuge before HPLC

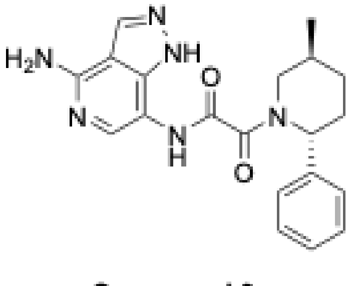


*N*-(4-amino-1*H*-pyrazolo[4,3-*c*]pyridin-7-yl)-2-((2*R*,5*S*)-5-methyl-2-phenylpiperidin-1-yl)-2-oxoacetamide, **Compound 3**
Oxamide starting material: **28**
Protection: SEM

Separation: Step 1: reverse phase HPLC (column: Chromatorex 18 SMB100-5T 100 x 19mm 5µm; mobile phase: 10-25 % 0-5 min $H_2O$/ACN/FA, flow: 30 mL / min (loading pump 4 mL / min acetonitrile)); Step 2: reverse phase HPLC (column: Chromatorex 18 SMB100-5T 100 x 19 mm 5um; mobile phase: 10-25% 0-5 min $H_2O$/ACN/FA, flow: 30 mL / min (loading pump 4 mL / min acetonitrile))
Yield: Step 1: 67%, Step 2: 67%
LCMS (ESI): $[M+H]^+$ m/z: calcd 378.2; found 379.4; Rt = 2.471 min. 1H NMR (600 MHz, DMSO-$d_6$) δ 0.88 – 1.11 (m, 3H), 1.22 – 1.43 (m, 1H), 1.60 – 1.75 (m, 1H), 1.80 – 1.96 (m, 1H), 2.04 – 2.26 (m, 2H), 2.70 – 3.11 (m, 1H), 3.68 – 4.13 (m, 1H), 4.97 – 5.66 (m, 1H), 6.22 – 8.02 (m, 8H), 8.22 – 8.56 (m, 1H), 9.59 – 10.78 (m, 1H), 11.36 – 14.13 (m, 2H).

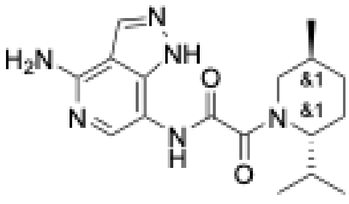


**Compound 4**

*rac*-*N*-(4-amino-1*H*-pyrazolo[4,3-*c*]pyridin-7-yl)-2-((2*R*,5*S*)-2-isopropyl-5-methylpiperidin-1-yl)-2-oxoacetamide, **Compound 4**
Oxamide starting material: **intermediate 4a**
Protection: SEM
Separation: Step 1: reverse phase HPLC (column: SunFire C18 100 x 19 mm, 5 um; mobile phase: 30-70 % 2-10 min MeCN, flow rate: 30 mL / min); Step 2: reverse phase HPLC (column: SunFire C18 100 x 19mm, 5um; mobile phase:0-50% 2-10 min MeCN + FA, flow rate: 30 mL / min)
Yield: Step 1: 11%; Step 2: 42%
$^1$H NMR (600 MHz, DMSO-$d_6$) (ppm) 0.23 – 0.82 (m, 3H), 0.82 – 1.02 (m, 6H), 1.17 – 1.47 (m, 1H), 1.51 – 1.91 (m, 3H), 1.94 – 2.26 (m, 1H), 2.89 – 3.09 (m, 1H), 3.30 – 3.31 (m, 1H), 3.59 – 3.67 (m, 1H), 3.90 – 4.13 (m, 1H), 6.61 – 7.51 (m, 2H), 7.72 – 8.18 (m, 2H), 9.27 – 10.49 (m, 1H), 12.40 – 13.45 (m, 1H). LCMS (ESI): $[M+1]^+$ m/z: calcd 344.2; found 345.2; Rt = 2.566 min.

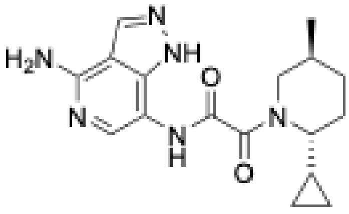


**Compound 5**

*N*-(4-amino-1*H*-pyrazolo[4,3-*c*]pyridin-7-yl)-2-((2*R*,5*S*)-2-cyclopropyl-5-methylpiperidin-1-yl)-2-oxoacetamide, **Compound 5**
Oxamide starting material: **intermediate 5a**
Protection: SEM
Separation: Step 1: reverse phase HPLC (40-65 % 0.5-6.5 min; 30 mL / min water-MeCN+$NH_3$ (loading pump 4 mL / min MeCN); column XBridge 19 x 100 mm (L)). Step 2: reverse phase HPLC (10-30 % 2-7 min; 30 mL / min water-acetonitrile+$NH_3$ (loading pump 4 mL / min acetonitrile); column xbridge C18 19 x 100 mm (L)).
Yield: Step 1: 8%; Step 2: 53 %
$^1$H NMR (600 MHz, DMSO-$d_6$) δ 0.18 – 0.40 (m, 2H), 0.40 – 0.60 (m, 2H), 0.81 – 1.01 (m, 3H), 1.22 – 1.83 (m, 4H), 1.83 – 1.98 (m, 1H), 1.99 – 2.10 (m, 1H), 2.81 – 3.21 (m, 1H), 3.36 – 4.12 (m, 2H), 6.79 (s, 2H), 7.39 – 7.76 (m, 1H), 8.17 (d, 1H), 9.34 – 10.43 (m, 1H), 12.49 – 13.37 (m, 1H). LCMS (ESI): $[M+H]^+$ m/z: calcd 343.2; found 343.2; Rt = 1.892 min.

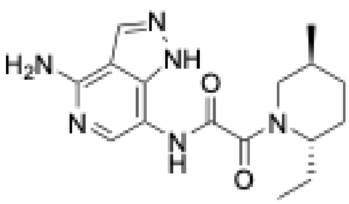


**Compound 6**

*N*-(4-amino-1*H*-pyrazolo[4,3-*c*]pyridin-7-yl)-2-((2*S*,5*S*)-2-ethyl-5-methylpiperidin-1-yl)-2-oxoacetamide, **Compound 6**
Oxamide starting material: **intermediate 6a**

Protection: SEM
Separation: Step 1: reverse phase HPLC (40-80% 0.5-6.5 min; 30 mL / min $H_2O/CH_3CN$ +$NH_3$ (loading pump 4 mL / min $CH_3CN$); column XBridge 19 x 100mm (L)) Step 2: reverse phase HPLC (20-40% 2-7min; 30 mL / min $H_2O/CH_3CN$ +$NH_3$ (loading pump 4mL / min $CH_3CN$); column xbridgeC18 19 x 100mm (L)).
Yield: Step 1: 50 %; Step 2: 51 %
Step 1: LCMS (ESI): [M+H]+ m/z: calcd 463.2; found 463.2; Rt = 3.664 min.
**Compound 6**: $^1$H NMR (600 MHz, DMSO-$d_6$) δ 0.73 – 0.86 (m, 3H), 0.91 – 1.06 (m, 3H), 1.25 – 1.34 (m, 1H), 1.37 – 1.47 (m, 1H), 1.51 – 1.61 (m, 1H), 1.67 – 2.02 (m, 4H), 2.92 – 4.43 (m, 3H), 7.38 – 9.14 (m, 4H), 10.69 (s, 1H), 12.49 – 13.75 (m, 1H). LCMS (ESI): [M+H]+ m/z: calcd 331.2; found 331.2; Rt = 2.155 min.

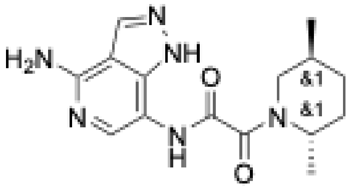


**Compound 7**

*rac*-*N*-(4-amino-1*H*-pyrazolo[4,3-*c*]pyridin-7-yl)-2-((2*R*,5*R*)-2,5-dimethylpiperidin-1-yl)-2-oxoacetamide, **Compound 7**
Oxamide starting material: **intermediate 7a**
Protection: SEM
Separation: Step 1: reverse phase HPLC ( column: XBridge BEH C18 100 x 19mm, 5um; mobile phase: 50-80% 0-1-6 min $H_2O$/MeOH/0.1% $NH_4OH$, flow rate: 30 mL / min (loading pump 4 mL / min MeOH) Step 2: reverse phase HPLC (column:XBridge BEH C18 100 x 19 mm, 5 µm; mobile phase: 10-10-65 % 0-2-5 min $H_2O$/MeOH/0.1% $NH_4OH$; flow rate: 30 mL / min (loading pump 4 mL / min MeOH))
Yield: Step 1: 27 %; Step 2: 6 %
Step 1: LCMS (ESI): $[M]^+$ m/z: calcd 446.2; found 447.2; Rt = 3.381 min.
**Compound 7:** $^1$H NMR (600 MHz, DMSO-$d_6$) δ 0.17 – 0.96 (m, 3H), 1.04 – 1.37 (m, 5H), 1.50 – 1.85 (m, 1H), 1.87 – 2.03 (m, 2H), 2.98 – 3.06 (m, 0.5H), 3.39 – 3.44 (m, 0.5H), 3.55 – 4.03 (m, 1H), 4.22 – 4.69 (m, 1H), 6.60 – 6.81 (m, 2H), 7.34 – 7.76 (m, 1H), 8.08 – 8.24 (m, 1H), 9.45 – 10.35 (m, 1H), 12.53 – 13.44 (m, 1H). LCMS (ESI): $[M]^+$ m/z: calcd 316.2; found 317.2; Rt = 2.018 min.

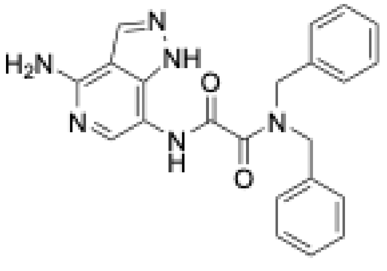


**Compound 12**

*N*[1]-(4-amino-1*H*-pyrazolo[4,3-*c*]pyridin-7-yl)-*N*[2],*N*[2]-dibenzyloxalamide, **Compound 12**
Oxamide starting material: **intermediate 12a**
Protection: SEM
Separation: Step 1: reverse phase HPLC (column: XBridge BEH C18 5um 130A; 65-65-80% 0-1-6 min $H_2O/CH_3OH$/0.1% $NH_4OH$, flow: 30 mL / min) Step 2: reverse phase HPLC (column: Chromatorex 18 SMB100-BT 100*19mm; 10-60% 0-5 min $H_2O/CH_3CN$/0.1%FA, flow: 30 mL / min)
Yield: Step 1: 13 %; Step 2: 74 %
Step 1: LCMS (ESI): $[M+H]^+$ m/z: calcd 531.3; found 531.0; Rt = 1.416 min; **Compound 12**: $^1$H NMR (600 MHz, DMSO-$d_6$) δ 3.96 – 4.72 (m, 4H), 6.27 – 7.22 (m, 3H), 7.26 – 7.31 (m, 3H), 7.31 – 8.24 (m, 8H), 9.79 – 10.69 (m, 1H), 12.66 – 13.43 (m, 1H). LCMS (ESI): $[M+H]^+$ m/z: calcd 401.2; found 401.2; Rt = 2.468 min.

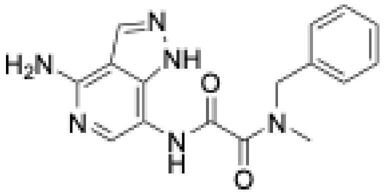

**Compound 13**

$N^1$-(4-amino-1*H*-pyrazolo[4,3-*c*]pyridin-7-yl)-$N^2$-benzyl-$N^2$-methyloxalamide, **Compound 13**
Oxamide starting material: **intermediate 13a**
Protection: THP
Separation: Step 2: HPLC (column: Chromatorex 18 SMB100-5T 100 x 19 mm 5µm; 5-5-35% 0-1.5-5min $H_2O$/ACN/0.1%FA, flow rate: 30mL / min)
Yield: Step 1: 97 %; Step 2: 47 %
Step 1: LCMS (ESI): $[M+H]^+$ m/z: calcd 409.2; found 409.2; Rt = 1.014 min; **Compound 13**: $^1$H NMR (500 MHz, DMSO-$d_6$) δ 2.80 – 3.08 (m, 3H), 4.46 – 4.85 (m, 2H), 7.11 – 7.50 (m, 7H), 7.78 – 8.52 (m, 2H), 10.45 – 10.72 (m, 1H), 12.22 – 14.19 (m, 2H).
LCMS (ESI): $[M+H]^+$ m/z: calcd 325.15; found 325.2; Rt = 1.269 min.

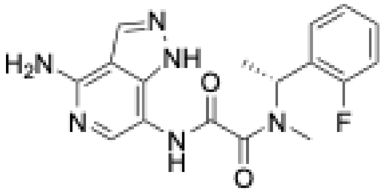

**Compound 14**

(*R*)-$N^1$-(4-amino-1*H*-pyrazolo[4,3-*c*]pyridin-7-yl)-$N^2$-(1-(2-fluorophenyl)ethyl)-$N^2$-methyloxalamide, **Compound 14**
Oxamide starting material: **intermediate 14a**
Protection: THP
Separation: Step 2: HPLC ( Device (Mobile Phase, Column): SYSTEM 5-30 % 0-5 min $H_2O$/ACN/0.1% FA, flow: 30 mL / min (loading pump 4 mL / min $H_2O$) target mass 356.36 column: Chromatorex 18 SMB100-5T 100 x 19 mm 5µm)
Yield: Step 1: Used as is; Step 2: 73 %
Step 1: LCMS (ESI): $[M+H]^+$ m/z: calcd 441.21; found 441.2; Rt = 1.066 min; **Compound 14**: $^1$H NMR (600 MHz, DMSO-$d_6$) δ 1.08 – 1.67 (m, 3H), 2.58 – 2.91 (m, 3H), 5.42 – 5.93 (m, 1H), 6.61 – 6.84 (m, 2H), 7.14 – 7.29 (m, 2H), 7.33 – 7.43 (m, 1H), 7.52 (td, 1H), 7.64 – 7.82 (m, 1H), 8.19 (d, 1H), 9.46 – 10.59 (m, 1H), 12.17 – 13.54 (m, 1H). LCMS (ESI): $[M+H]^+$ m/z: calcd 357.16; found 357.2; Rt = 1.739 min.

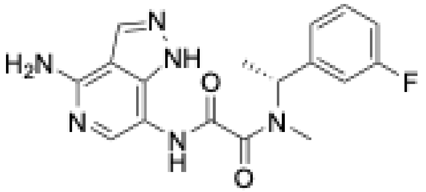

**Compound 15**

(*R*)-$N^1$-(4-amino-1*H*-pyrazolo[4,3-*c*]pyridin-7-yl)-$N^2$-(1-(3-fluorophenyl)ethyl)-$N^2$-methyloxalamide, **Compound 15**
Oxamide starting material: **intermediate 15a**
Protection: THP
Separation: Step 2: HPLC (Device (Mobile Phase, Column): SYSTEM 10-35% 0-5min $H_2O$/ACN/0.1% FA, flow: 30 mL / min (loading pump 4 mL / min $H_2O$) target mass 356.36 column: Chromatorex 18 SMB100-5T 100 x 19 mm 5µm) and repurified (Device (Mobile Phase, Column): SYSTEM 10-60% 0-5min $H_2O$/MeOH/0.1% $NH_4OH$, flow: 30mL / min (loading pump 4mL / min MeOH) target mass 356.35 column: XBridge C18 100 x 19 mm, 5µm)
Yield: Step 1: Used as is; Step 2: 15 %
Step 1: LCMS (ESI): $[M+H]^+$ m/z: calcd 441.21; found 441.2; Rt = 1.080 min; **Compound 15**: $^1$H NMR (600 MHz, DMSO-$d_6$) δ 1.17 – 1.65 (m, 3H), 2.59 – 2.87 (m, 3H), 4.97 – 5.79 (m, 1H), 6.58 – 6.89 (m, 2H), 6.95 – 7.21 (m, 2H), 7.23 – 7.47 (m, 2H), 7.48 – 7.74 (m, 1H), 8.14 – 8.25 (m, 1H), 9.54 – 10.68 (m, 1H), 12.42 – 13.51 (m, 1H). LCMS (ESI): $[M+H]^+$ m/z: calcd 357.16; found 357.0; Rt = 1.935 min.

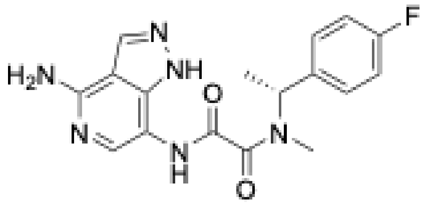

**Compound 16**

($R$)-$N^1$-(4-amino-1$H$-pyrazolo[4,3-$c$]pyridin-7-yl)-$N^2$-(1-(4-fluorophenyl)ethyl)-$N^2$-methyloxalamide, **Compound 16**
Oxamide starting material: **intermediate 16a**
Protection: THP
Separation: Step 1: HPLC (column: XBridge C18 5µm 130A; 40-40-60% 0-1.5-5 min $H_2O/CH_3OH$/0.1% $NH_4OH$, flow: 30 mL / min) Step 2: HPLC (column: XBridge BEH18 SMB100-BT 100 x 19 mm; 10-45% 0-1-5 min $H_2O/CH_3CN$/0.1%NH4OH, flow: 30 mL / min)
Yield: Step 1: 46 %; Step 2: 43 %
Step 1: LCMS (ESI): $[M+H]^+$ m/z: calcd 441.23; found 441.2; Rt = 2.743 min; **Compound 16**: $^1$H NMR (500 MHz, DMSO-$d_6$) δ 1.23 – 1.62 (m, 3H), 2.52 – 2.86 (m, 3H), 4.94 – 5.82 (m, 1H), 6.61 – 6.92 (m, 2H), 6.96 – 7.24 (m, 2H), 7.37 – 7.44 (m, 1H), 7.44 – 7.51 (m, 1H), 7.64 – 7.76 (m, 1H), 8.11 – 8.25 (m, 1H), 9.46 – 10.88 (m, 1H), 12.47 – 13.32 (m, 1H). LCMS (ESI): $[M+H]^+$ m/z: calcd 357.16; found 357.2; Rt = 1.111 min.

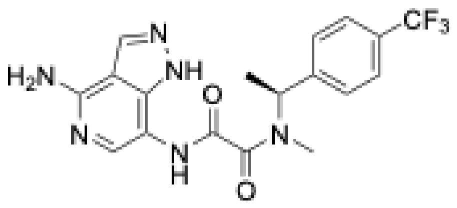

**Compound 17**

($S$)-$N^1$-(4-amino-1$H$-pyrazolo[4,3-$c$]pyridin-7-yl)-$N^2$-methyl-$N^2$-(1-(4-(trifluoromethyl)phenyl)ethyl)oxalamide, **Compound 17**
Oxamide starting material: **intermediate 17a**
Protection: THP
Separation: Step 2: HPLC (0-2-10 min, 8-15-55 $H_2O$ / MeOH / 0.1 $NH_4OH$, flow 30 mL / min ((loading pump 4 mL MeOH), target mass 407, column: XBridge BEH C18 100 x 19 mm,5 µM); Chiral separation: (Column: Chiralcel OJ-H (250 x 20 mm, 5 mkm); Mobile phase: Hexane(0.1%DEA)-IPA-MeOH, 80-10-10. Flow Rate: 12 mL / min; Column Temperature: 24$^o$C; Wavelength: 205 nm; RetTime = 39.73 min)
Yield: Step 1: Used as is in the next step; Step 2: 5 %; Chiral separation: 86 %
Step 1: LCMS (ESI): $[M+H]^+$ m/z: calcd 491.2; found 491.2; Rt = 1.167 min; Step 2: $^1$H NMR (600 MHz, DMSO-$d_6$) δ 1.29 – 1.67 (m, 3H), 2.57 – 2.92 (m, 3H), 5.36 – 5.87 (m, 1H), 6.61 – 6.91 (m, 2H), 7.49 – 7.59 (m, 1H), 7.61 – 7.65 (m, 1H), 7.65 – 7.71 (m, 1H), 7.72 – 7.78 (m, 2H), 8.13 – 8.21 (m, 1H), 9.65 – 10.73 (m, 1H), 12.47 – 13.25 (m, 1H). LCMS (ESI): $[M+H]^+$ m/z: calcd 407.16; found 407.2; Rt = 1.010 min. **Compound 17**: $^1$H NMR (600 MHz, DMSO-$d_6$) δ 1.25 – 1.71 (m, 3H), 2.74 (d, 3H), 5.36 – 5.84 (m, 1H), 6.60 – 6.85 (m, 2H), 6.89 – 7.68 (m, 3H), 7.69 – 7.78 (m, 2H), 8.12 – 8.22 (m, 1H), 9.65 – 10.67 (m, 1H), 12.62 – 13.36 (m, 1H). LCMS (ESI): $[M+H]^+$ m/z: calcd 407.16; found 407.0; Rt = 2.269 min.

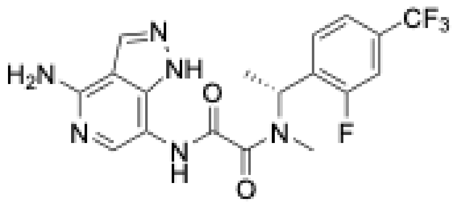

**Compound 18**

($R$)-$N^1$-(4-amino-1$H$-pyrazolo[4,3-$c$]pyridin-7-yl)-$N^2$-(1-(2-fluoro-4-(trifluoromethyl)phenyl)ethyl)-$N^2$-methyloxalamide, **Compound 18**
Oxamide starting material: **intermediate 18a**
Protection: THP
Separation: Step 2: HPLC (column: Chromatorex 18 SMB100-5T 100 x 19 mm 5 um; 10-10-30% 0-1.3-5.3 min $H_2O$/ACN/0.1%FA, flow rate: 30 mL / min) Chiral separation: chiral HPLC (Column: CHIRALPAK IC (250 x 30 mm, 10 mkm); Mobile Phase: Hexane:IPA:MeOH:DEA, 70:15:15:0.2; Injection Volume: 900 µL)
Yield: Step 1: Used as is in the next step; Step 2: 5 %.; Chiral separation: 4 %
Step 1: LCMS (ESI): $[M+H]^+$ m/z: calcd 509.2; found 509.2; Rt = 1.046 min **Compound 18**: $[\alpha]^{21}$D = +98.24/+98.20 deg (c = 0.25g/100mL MeOH); $^1$H NMR (600 MHz, DMSO-$d_6$) δ 1.55 – 1.68 (m, 3H), 2.66 – 2.96 (m, 3H), 5.76 – 5.96 (m, 1H), 6.63 – 6.86 (m, 2H), 7.59 – 7.70 (m,

2H), 7.70 – 7.80 (m, 2H), 8.12 – 8.23 (m, 1H), 10.17 – 10.57 (m, 1H), 12.51 – 12.86 (m, 1H). LCMS (ESI): $[M+H]^+$ m/z: calcd 425.2; found 425.2; Rt = 2.641 min.

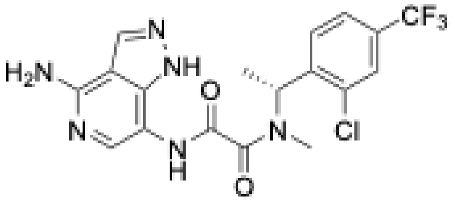

**Compound 19**

(*R*)-*N*[1]-(4-amino-1*H*-pyrazolo[4,3-*c*]pyridin-7-yl)-*N*[2]-(1-(2-chloro-4-(trifluoromethyl)phenyl)ethyl)-*N*[2]-methyloxalamide, **Compound 19**
Oxamide starting material: **intermediate 19a**
Protection: THP
Separation: Step 2: HPLC (5-30% 0-5 min H2O/MeCN/0.1% FA, flow: 30 mL / min, column: Chromatorex 18 SMB100-5T 100 x 19 mm 5 um), Chiral separation: RT (Chiralpak AD-H (250*4.6 mm, 5 mkm); Hexane(0.1% EDTA):IPA:MeOH, 50:25:25, Flow Rate: 0.6 mL / min) = 14.493 min
Yield: Step 1: Used as is in the next step; Step 2: 41 %; Chiral separation: 71 %
Step 1: LCMS (ESI): $[M+H]^+$ m/z: calcd 525.2; found 525.2; Rt = 1.064 min.; Step 2: LCMS (ESI): $[M+H]^+$ m/z: calcd 441.2; found 441.0; Rt = 1.083 min; Compound 19: $[\alpha]^{21}D$ = +91.10 deg (c = 0.2g/100mL, MeOH). $^1H$ NMR (600 MHz, DMSO-$d_6$) δ 1.16 – 1.72 (m, 3H), 2.58 – 2.92 (m, 3H), 5.29 – 5.89 (m, 1H), 6.50 – 6.97 (m, 2H), 7.18 – 7.93 (m, 4H), 8.10 – 8.29 (m, 1H), 9.60 – 10.54 (m, 1H), 12.46 – 13.36 (m, 1H). LCMS (ESI): $[M+H]^+$ m/z: calcd 441.2; found 441.2; Rt = 2.313 min.

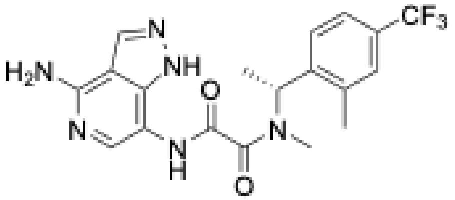

**Compound 20**

(*R*)-*N*[1]-(4-amino-1*H*-pyrazolo[4,3-*c*]pyridin-7-yl)-*N*[2]-methyl-*N*[2]-(1-(2-methyl-4-(trifluoromethyl)phenyl)ethyl)oxalamide, **Compound 20**
Oxamide starting material: **intermediate 20a**
Protection: THP
Separation: Step 2: HPLC (column: XBridge C18 100 x 19 mm, 5µm; 30-80% 0-5min $H_2O$/MeOH/0.1%$NH_4OH$, flow: 30 mL / min, flow rate: 30 mL / min), Chiral separation: chiral HPLC (Column: CHIRALPAK AD-H (250 × 20 mm, 5 µm)-V; Mobile Phase: Hexane(0.1% DEA):MeOH:IPA, 60:20:20; Injection Volume: 900 µl; flow rate: 12 mL / min)
Yield: Step 1: Used as is in the next step; Step 2: Used as is in the chiral separation; Chiral separation: 6 %
Step 1: LCMS (ESI): $[M+H]^+$ m/z: calcd 505.21; found 505.2; Rt = 1.063 min; **Compound 20**: LCMS (ESI): $[M+H]^+$ m/z: calcd 420.18; found 421.2; Rt = 2.599 min; $^1H$ NMR (500 MHz, DMSO-$d_6$) δ 1.48 – 1.73 (m, 3H), 2.28 – 2.37 (m, 3H), 2.53 – 2.75 (m, 3H), 5.29 – 5.93 (m, 1H), 6.56 – 6.92 (m, 2H), 7.33 – 7.73 (m, 4H), 8.10 – 8.24 (m, 1H), 9.54 – 10.61 (m, 1H), 12.57 – 13.45 (m, 1H).

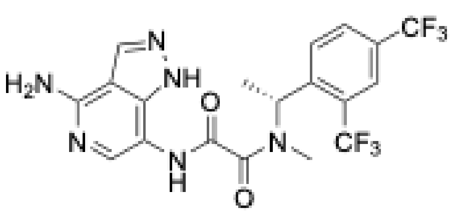

**Compound 21**

(*R*)-*N*[1]-(4-amino-1*H*-pyrazolo[4,3-*c*]pyridin-7-yl)-*N*[2]-(1-(2,4-bis(trifluoromethyl)phenyl)ethyl)-*N*[2]-methyloxalamide, **Compound 21**
Oxamide starting material: **intermediate 21a**
Protection: THP

Separation: Step 2: HPLC (1st run: 33-40-80% 0-2-7-7.1 min; 30 mL / min water-MeOH+NH3 (loading pump 4 mL / min MeOH+NH3); column Xbridge C18 5 uM 19 x 100 mm; 2nd run: 53-60-90-100% 0-2-7-7.1 min; 30 mL / min water-MeOH (loading pump 4 mL / min MeOH); column Kinetex PFP 5 uM 21.2 x 100 mm (R)), Chiral separation: chiral chromatography (Column: CHIRALPAK AD (250 x 30 mm, 10 mkm)-II; Mobile Phase: Hexane(0.1% DEA):IPA:MeOH, 50:25:25 Flow Rate: 30 mL / min) Yield: Step 1: Used as is in the next step; Step 2: 12 %; Chiral separation: 51 %
Step 1: LCMS (ESI): [M+H]+ m/z: calcd 475.2; found 475.2; Rt = 1.599 min. **Compound 21**: LCMS (ESI): [M+H]+ m/z: calcd 475.0; found 475.0; Rt = 4.068 min.

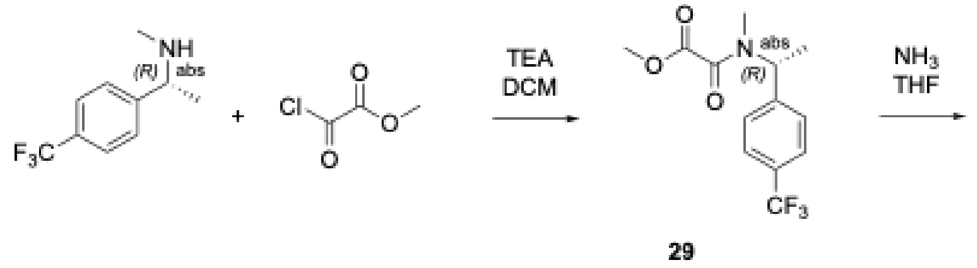


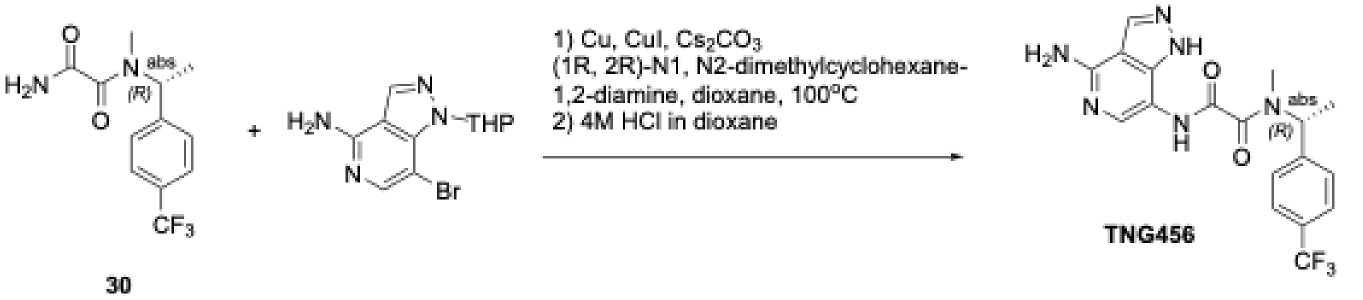


Methyl (*R*)-2-(methyl(1-(4-(trifluoromethyl)phenyl)ethyl)amino)-2-oxoacetate (**29**).
To a solution of (1*R*)-1-[4-(trifluoromethyl)phenyl]ethanamine (4 g, 17.73 mmol, HCl) and TEA (39.00 mmol, 5.44 mL) in THF (50 mL) was added methyl 2-chloro-2-oxo-acetate (2.39 g, 19.50 mmol) at room temperature under Ar atmosphere. After stirring at room temperature for 5 min the mixture was filtered through a pad of $Na_2SO_4$, evaporated to dryness, added THF (50mL), and concentrated again. The residue was dissolved in THF and washed with saturated $NaHCO_3$ (aq), brine, dried, and evaporated to give methyl 2-[methyl-[(1*R*)-1-[4-(trifluoromethyl)phenyl]ethyl]amino]-2-oxo-acetate, **29** (3.9 g, 13.48 mmol, 76% yield) as a colorless gum.
LCMS( ESI): [M+H]+ m/z: calcd 289.09; found 290.0; Rt = 1.249 min.

(*R*)-*N*[1]-methyl-*N*[1]-(1-(4-(trifluoromethyl)phenyl)ethyl)oxalamide (**30**).
Ammonia (229.62 mg, 13.48 mmol) was bubbled through a solution of **29** (3.9 g, 13.48 mmol) in MeOH (50 mL) at room temperature. After stirring for 18 h, the reaction mixture was evaporated to dryness, dissolved in DCM, and washed with saturate $NaHCO_3$ (aq), dried and evaporated to give *N'*-methyl-*N'*-[(1*R*)-1-[4-(trifluoromethyl)phenyl]ethyl]oxamide, **30** (3.4 g, 12.40 mmol, 92% yield) as an orange solid.
LCMS (ESI): [M- C3H5N2O2]+ m/z: calcd 274.09; found 173.2; Rt = 2.770 min.

(*R*)-*N*[1]-(4-amino-1*H*-pyrazolo[4,3-*c*]pyridin-7-yl)-*N*[2]-methyl-*N*[2]-(1-(4-(trifluoromethyl)phenyl)ethyl)oxalamide, **TNG456**.
**30** (1.5 g, 4.38 mmol), 7-bromo-2-tetrahydropyran-2-yl-pyrazolo[4,3-c]pyridin-4-amine (1.30 g, 4.38 mmol), copper (13.90 mg, 218.79 µmol), copper (I) iodide (416.68 mg, 2.19 mmol) , (1*R*,2*R*)-*N*[1],*N*[2]-dimethylcyclohexane-1,2-diamine (466.81 mg, 3.28 mmol) and cesium carbonate (2.85 g, 8.75 mmol) were mixed in dioxane (40 mL). The resulting mixture was purged with Ar for 30 seconds. The vials were sealed and heated at 100 °C for 48 h. The reaction mixture was cooled and filtered. The filter cake was washed with MeOH (100 mL) and the filtrate was concentrated in vacuo and redissolved in MeOH (25 mL) and hydrogen chloride solution 4.0 M in dioxane (4.38 mmol, 25 mL) was added. After 1 h the reaction mixture was filtered, rinsed with MeOH, and evaporated. The residue was purified by HPLC (23-30-80-100 % 0-2-7-7.1 min; 30 mL/min water(+ 15mM $NH_4HCO_3$)-MeOH (loading pump 4 mL / min MeOH); target mass 407 column PFP C18 5 µM 19 x 100 mm to afford (*R*)-*N*[1]-(4-amino-1*H*-pyrazolo[4,3-*c*]pyridin-7-yl)-*N*[2]-methyl-*N*[2]-(1-(4-(trifluoromethyl)phenyl)ethyl)oxalamide, **TNG456** (407 mg, 1.00 mmol, 23% yield). Optical rotatory power (c = 0.5g / 100mL, MeOH, 21°C) => +46.34. $^1$H NMR (600 MHz, DMSO-$d_6$) $\delta$ 1.52 – 1.69 (m, 3H), 2.58 – 2.88 (m, 3H), 3.40 – 3.44 (m, 1H), 5.52 – 5.87 (m, 1H), 6.65 (s, 2H), 7.49 – 7.59 (m, 1H), 7.62 – 7.66 (m, 1H), 7.66 – 7.71 (m, 1H), 7.73 – 7.78 (m, 2H), 8.15 – 8.19 (m, 1H), 9.85 – 12.23 (m, 1H). LCMS(ESI): [M+H]+ m/z: calcd 406.15; found 407.0; Rt = 2.178 min.

**NMR SPECTRA AND HPLC/LCMS TRACES OF FINAL COMPOUNDS**

**COMPOUND 3**

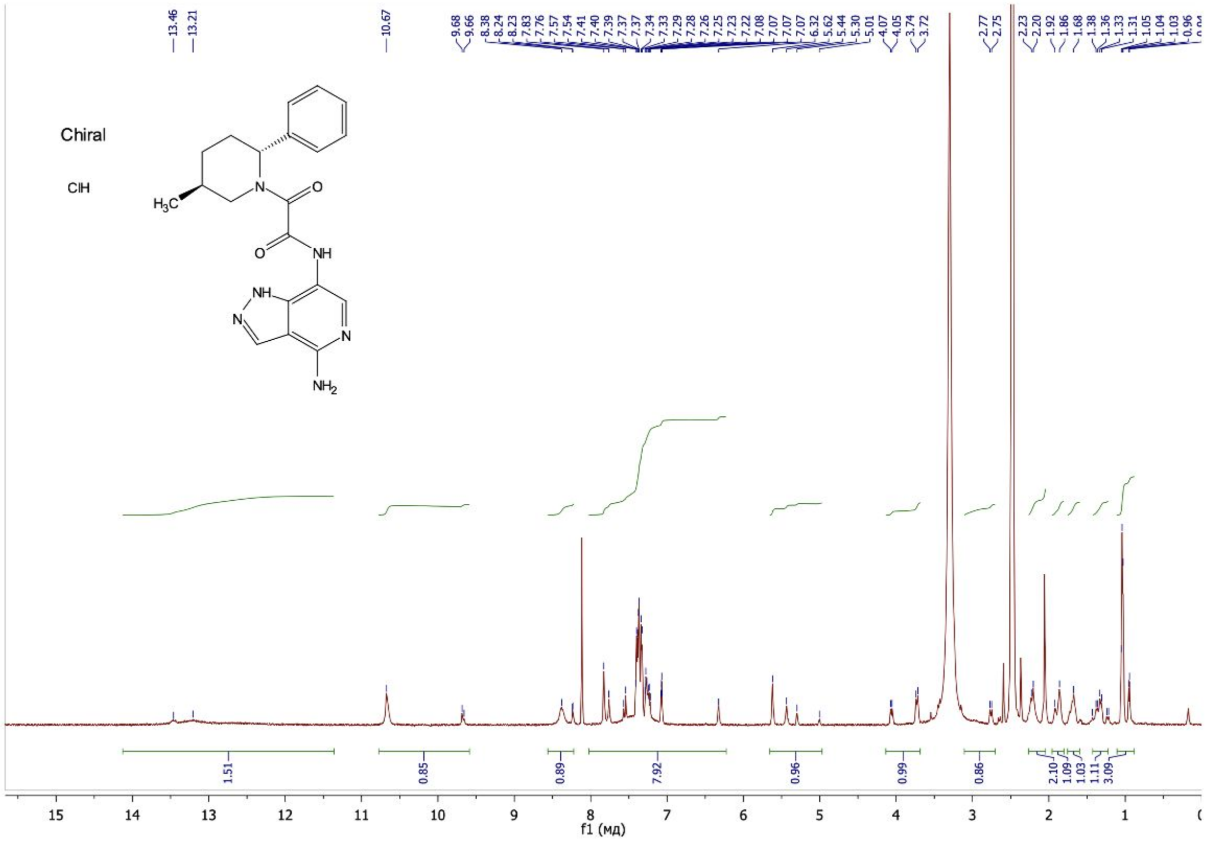
Chiral
ClH
H3C
N
O
O
NH
NH
N
N
NH2
13.46
13.21
10.67
1.51
0.85
0.89
7.92
0.96
0.99
0.86
2.10
1.09
1.03
1.11
3.09
f1 (мд)

MaxPeak: 98.41%
Ret_Time: 2.471 min

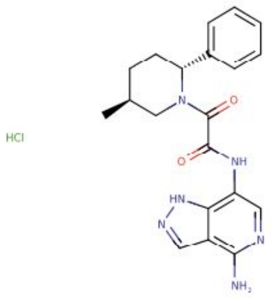


**Mol Wt** 414.89

**Exact Mass** 378.2

| # | Time | Area% |
|---|---|---|
| 1 | 2.471 | 98.41 |
| 2 | 2.581 | 1.59 |

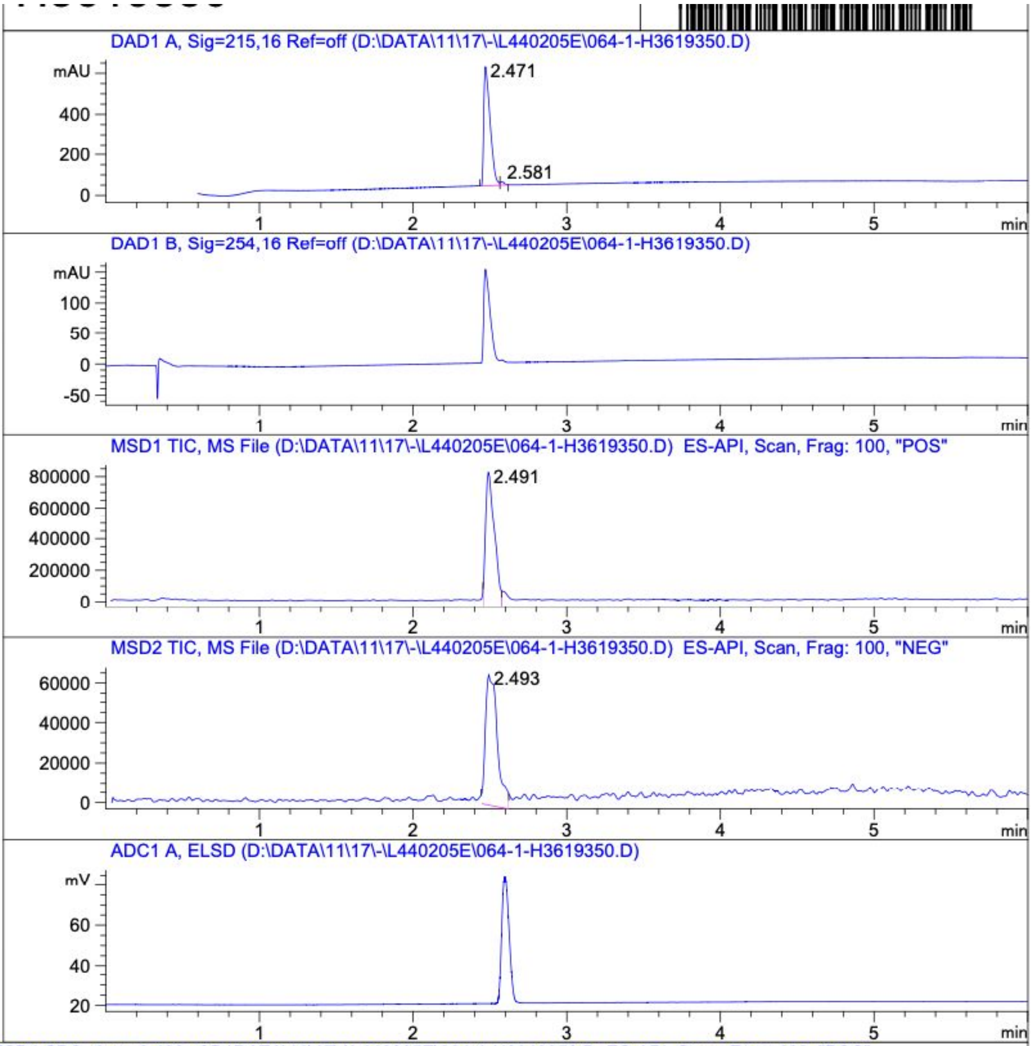


RT 2.491

RT 2.493

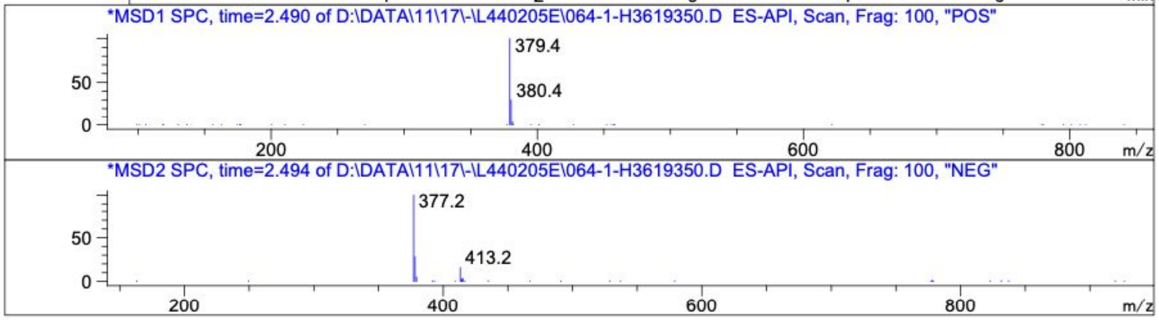


**COMPOUND 4**

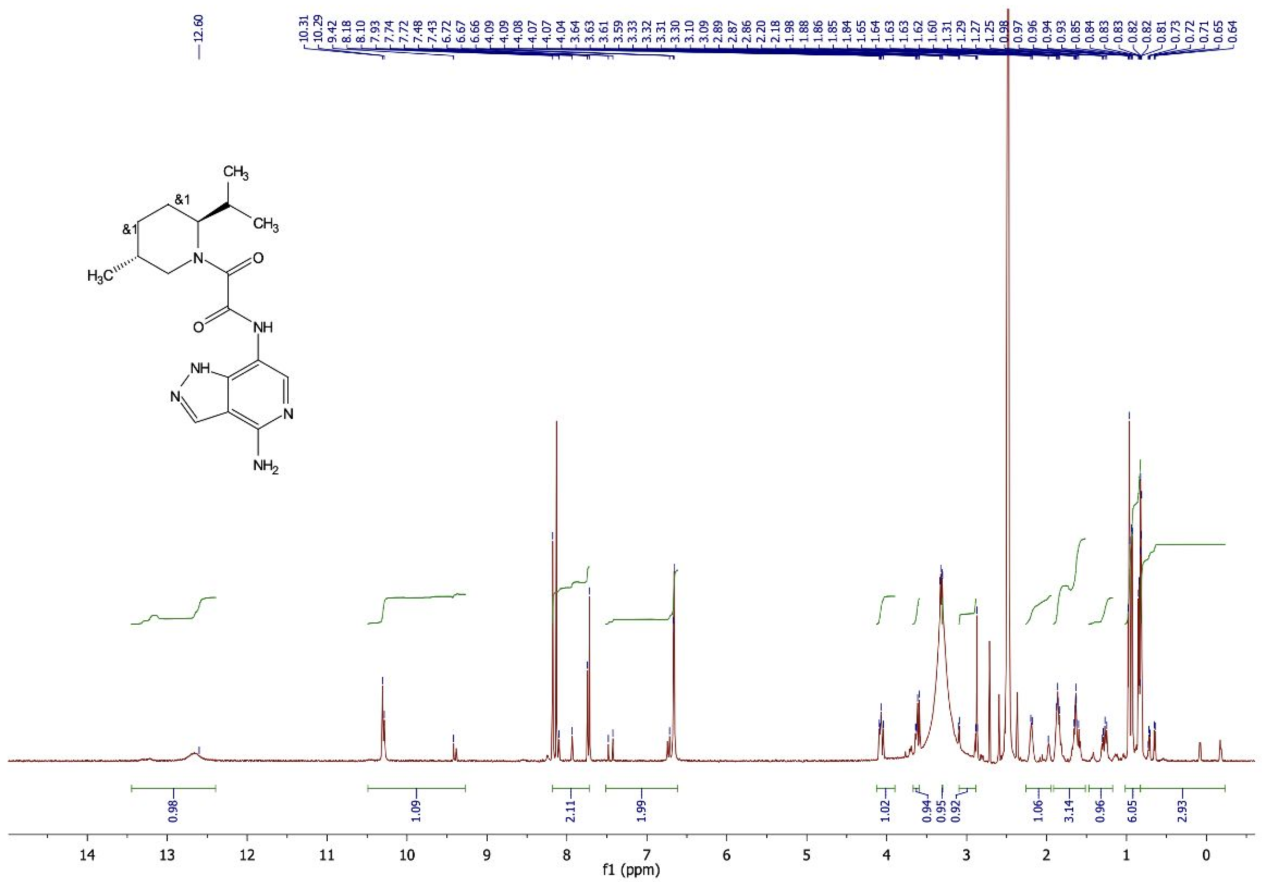

12.60
10.31
10.29
9.42
8.18
8.10
7.93
7.74
7.72
7.48
7.43
6.72
6.67
6.66
4.09
4.09
4.08
4.07
4.04
3.64
3.63
3.61
3.59
3.33
3.32
3.31
3.30
3.10
3.09
2.89
2.87
2.86
2.20
2.18
1.98
1.88
1.86
1.85
1.84
1.65
1.64
1.63
1.63
1.62
1.60
1.31
1.29
1.27
1.25
0.98
0.97
0.96
0.94
0.93
0.85
0.84
0.83
0.83
0.82
0.82
0.81
0.73
0.72
0.71
0.65
0.64
CH3
&1
CH3
&1
H3C
N
O
O
NH
NH
N
N
NH2
0.98
1.09
2.11
1.99
1.02
0.94
0.95
0.92
1.06
3.14
0.96
6.05
2.93
14
13
12
11
10
9
8
7
6
5
4
3
2
1
0
f1 (ppm)

MaxPeak: 97.31%
Ret_Time: 2.537 min

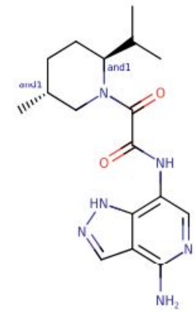


**Mol Wt 344.41**

**Exact Mass 344.22**

| # | Time | Area% |
|---|---|---|
| 1 | 2.470 | 2.69 |
| 2 | 2.537 | 97.31 |

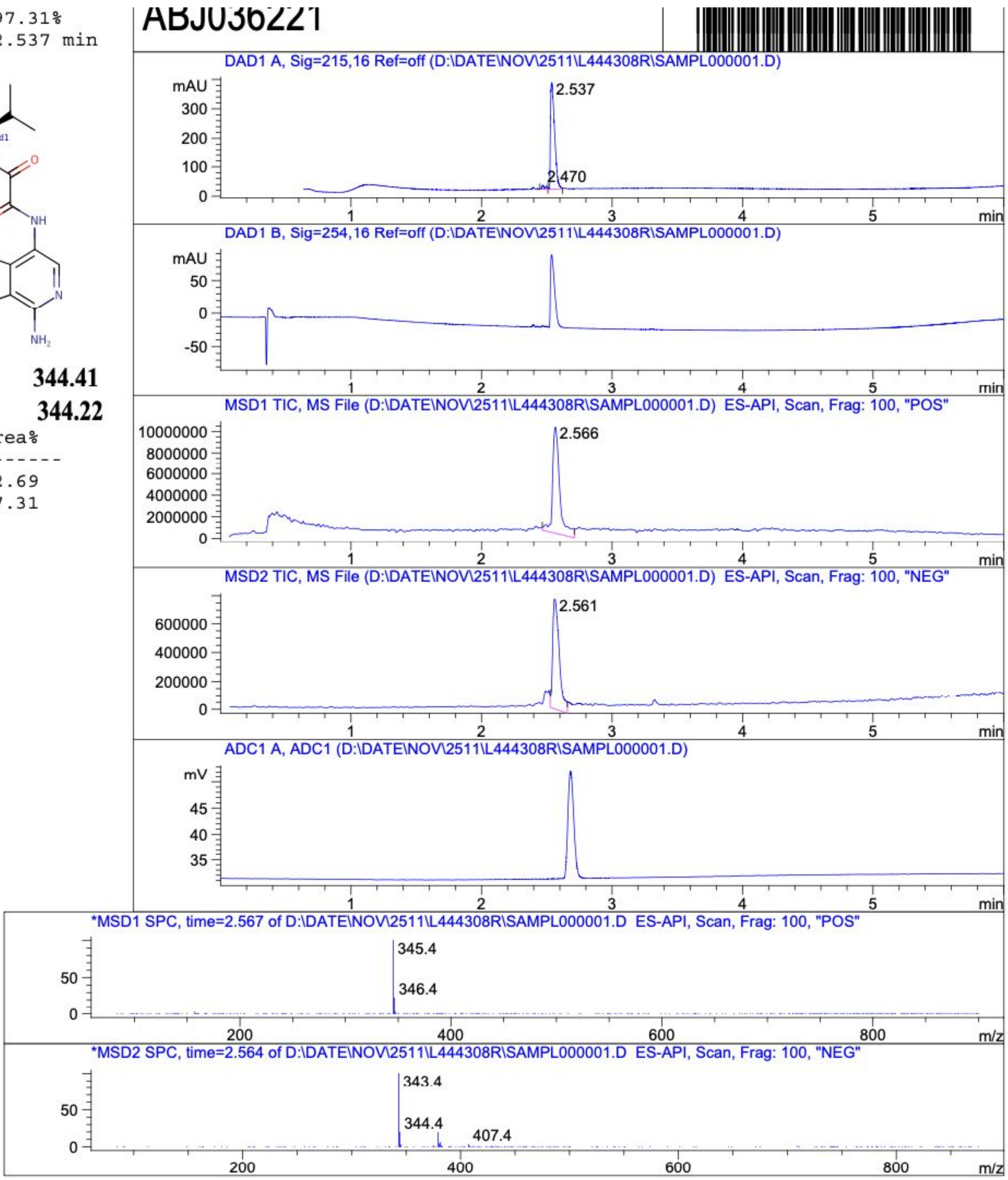


RT 2.566

RT 2.561

**COMPOUND 5**

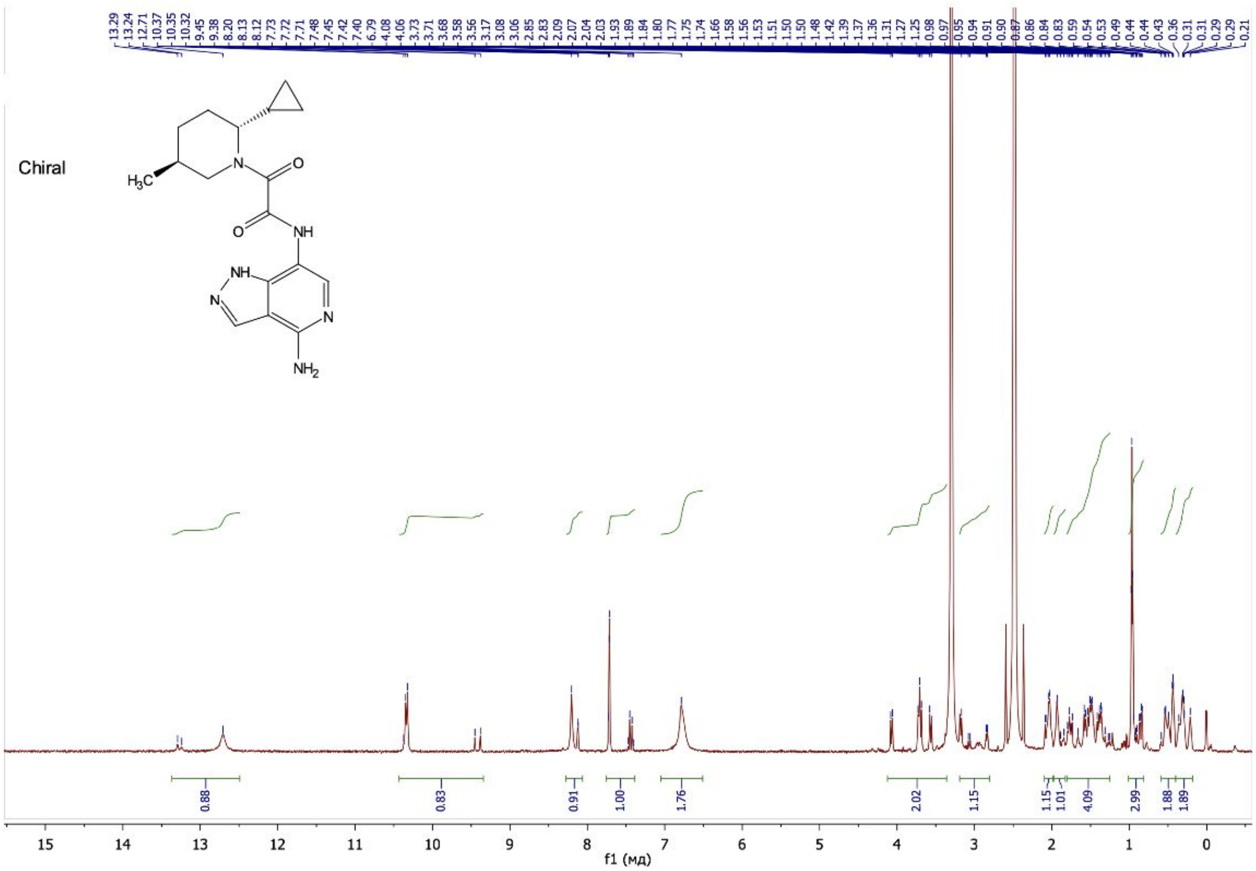

**COMPOUND 6**

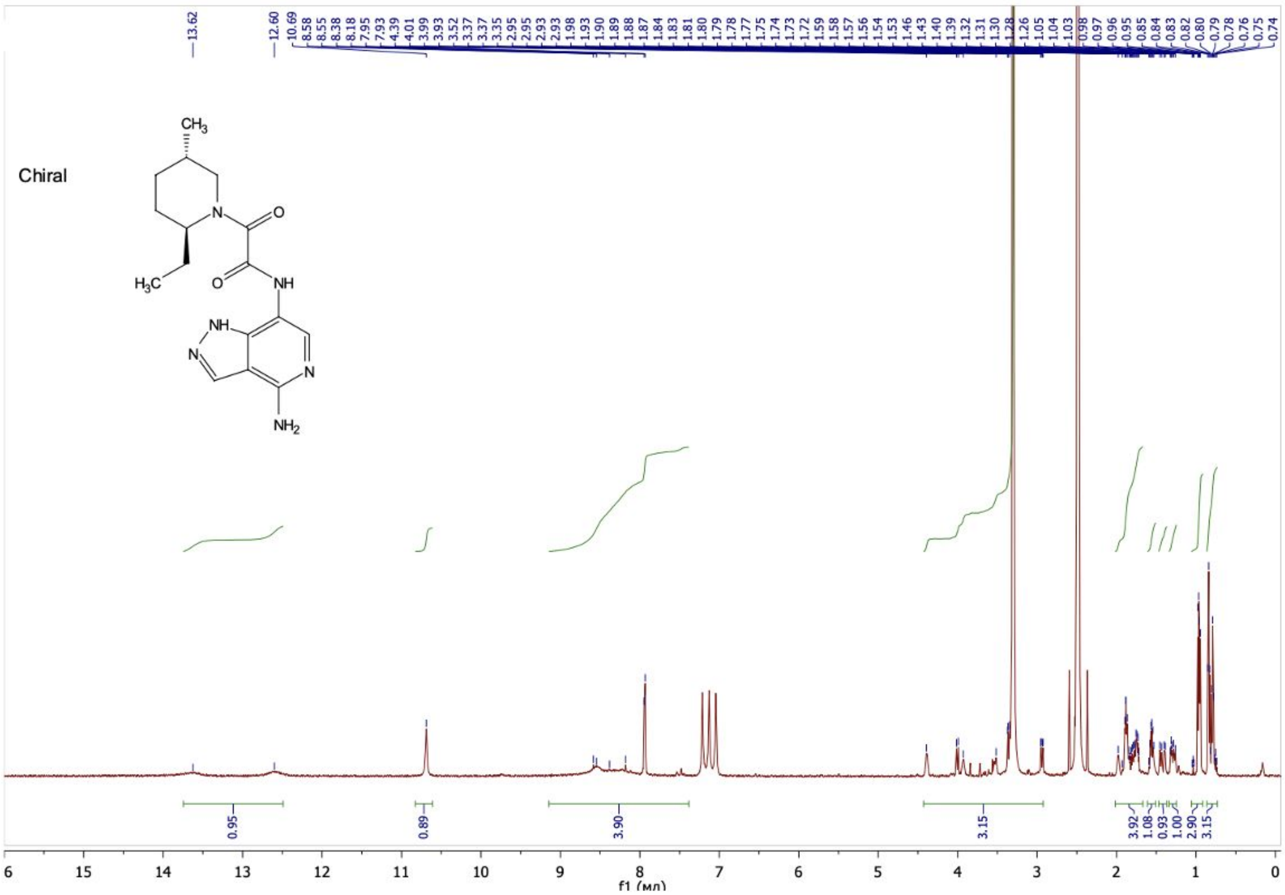
Chiral
f1 (ppm)

MaxPeak: 98.59%
Ret_Time: 1.812 min

ABO038747

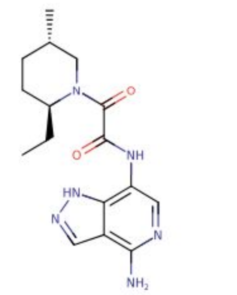

**Mol Wt** 330.38
**Exact Mass** 330.2

| # | Time | Area% |
|---|---|---|
| 1 | 1.812 | 98.59 |
| 2 | 1.901 | 1.41 |

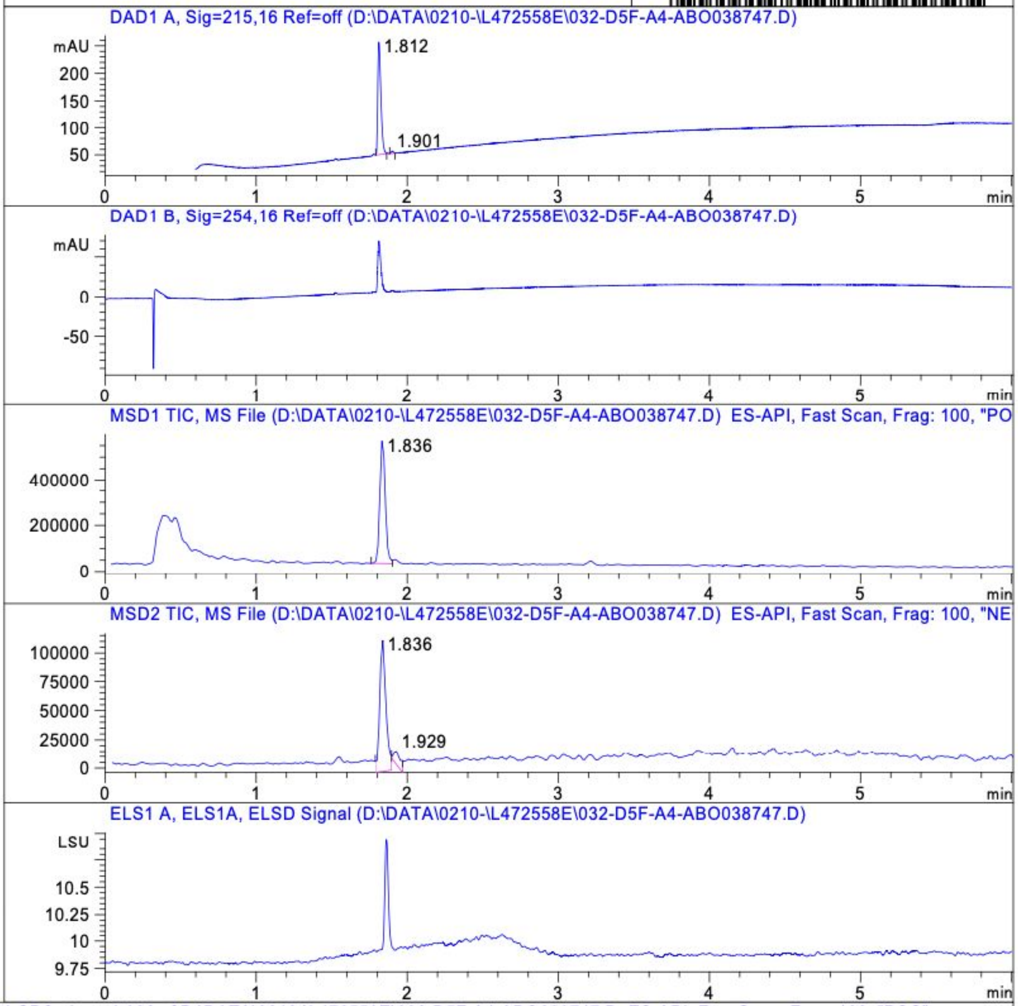


RT 1.836

RT 1.836

RT 1.929

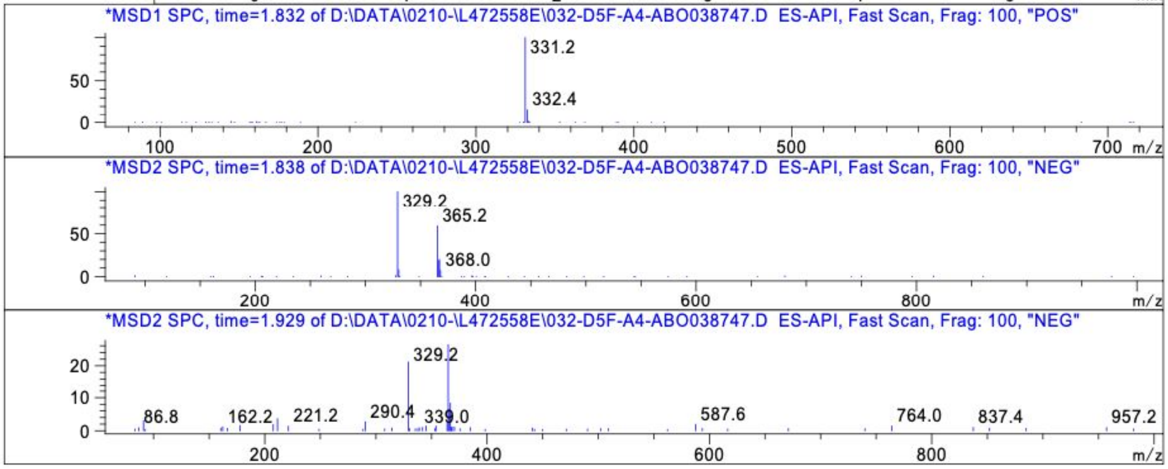


**COMPOUND 7**

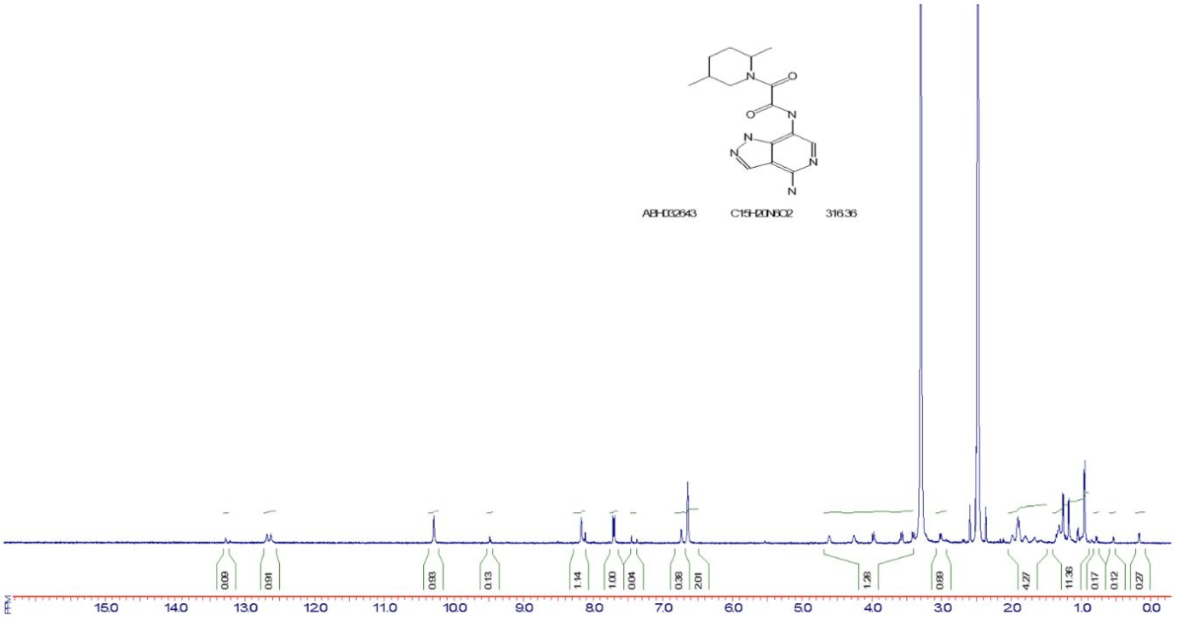

**COMPOUND 8**

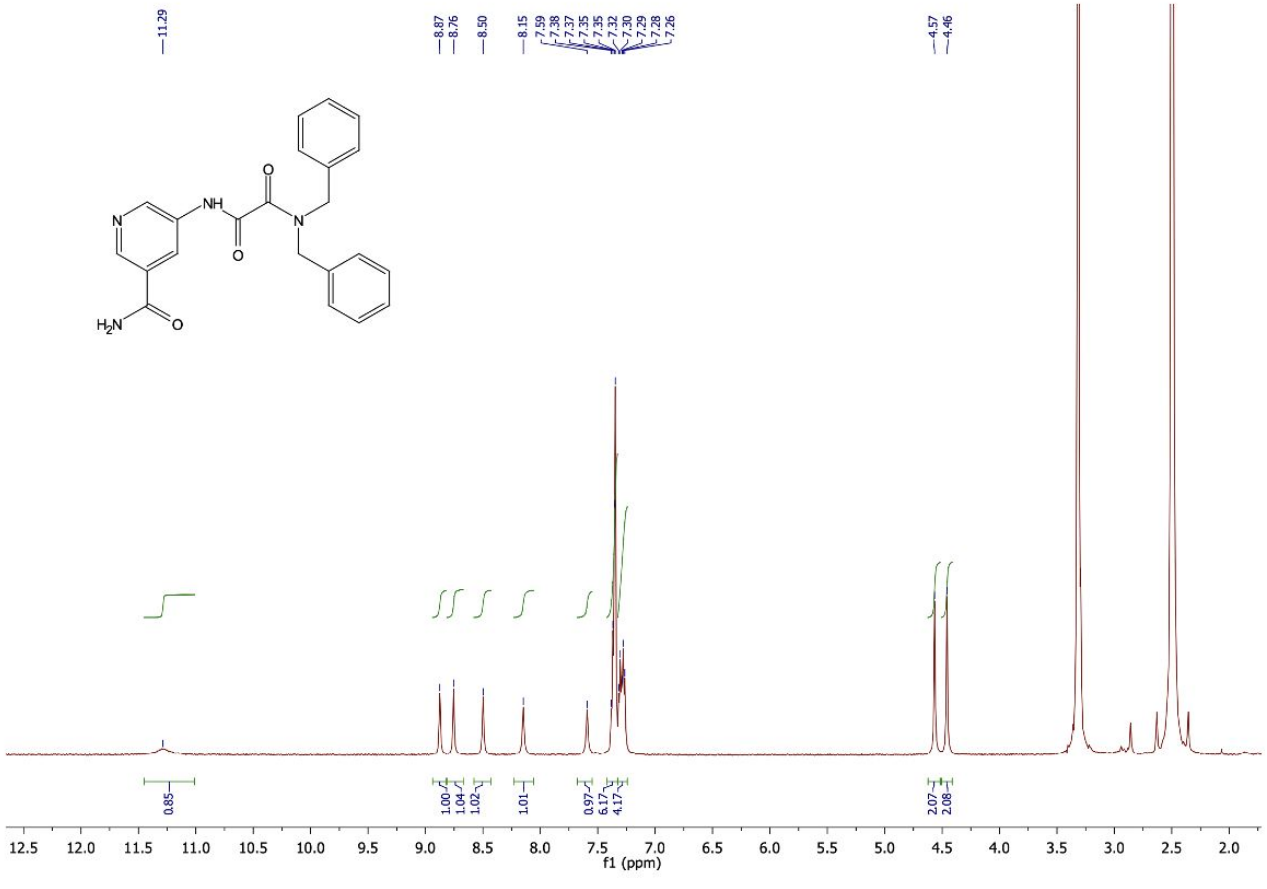

```
MaxPeak:  98.13%
Ret_Time: 3.147 min
```

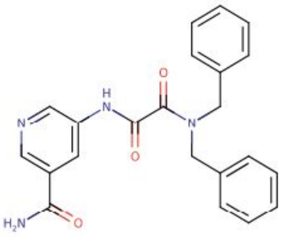


**Mol Wt 388.42**

**Exact Mass 388.17**

| # | Time | Area% |
|---|---|---|
| 1 | 2.556 | 1.87 |
| 2 | 3.147 | 98.13 |

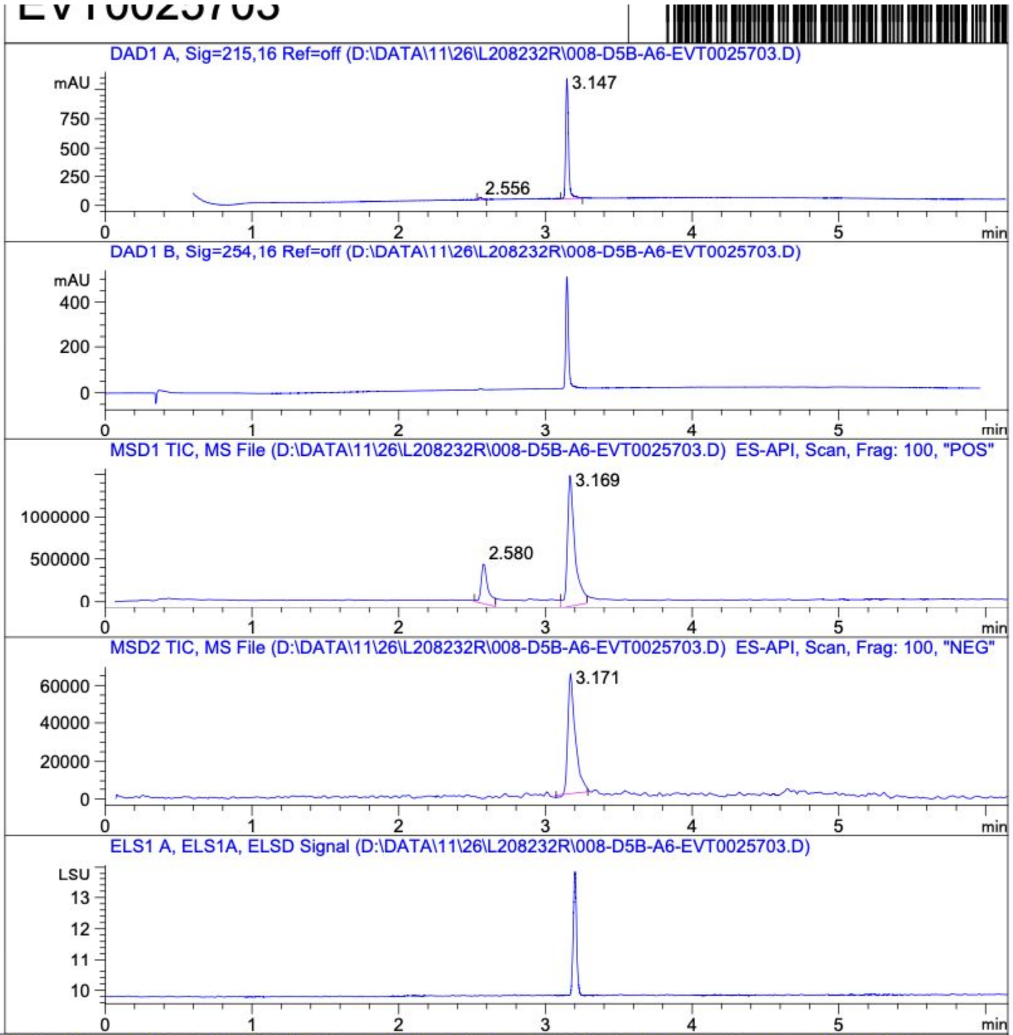


RT 2.580

RT 3.169

RT 3.171

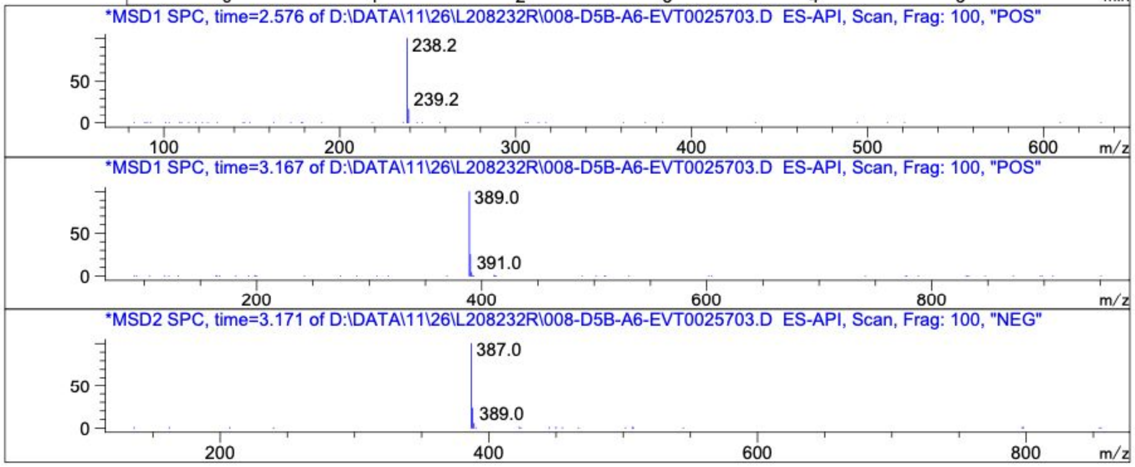


**COMPOUND 9**

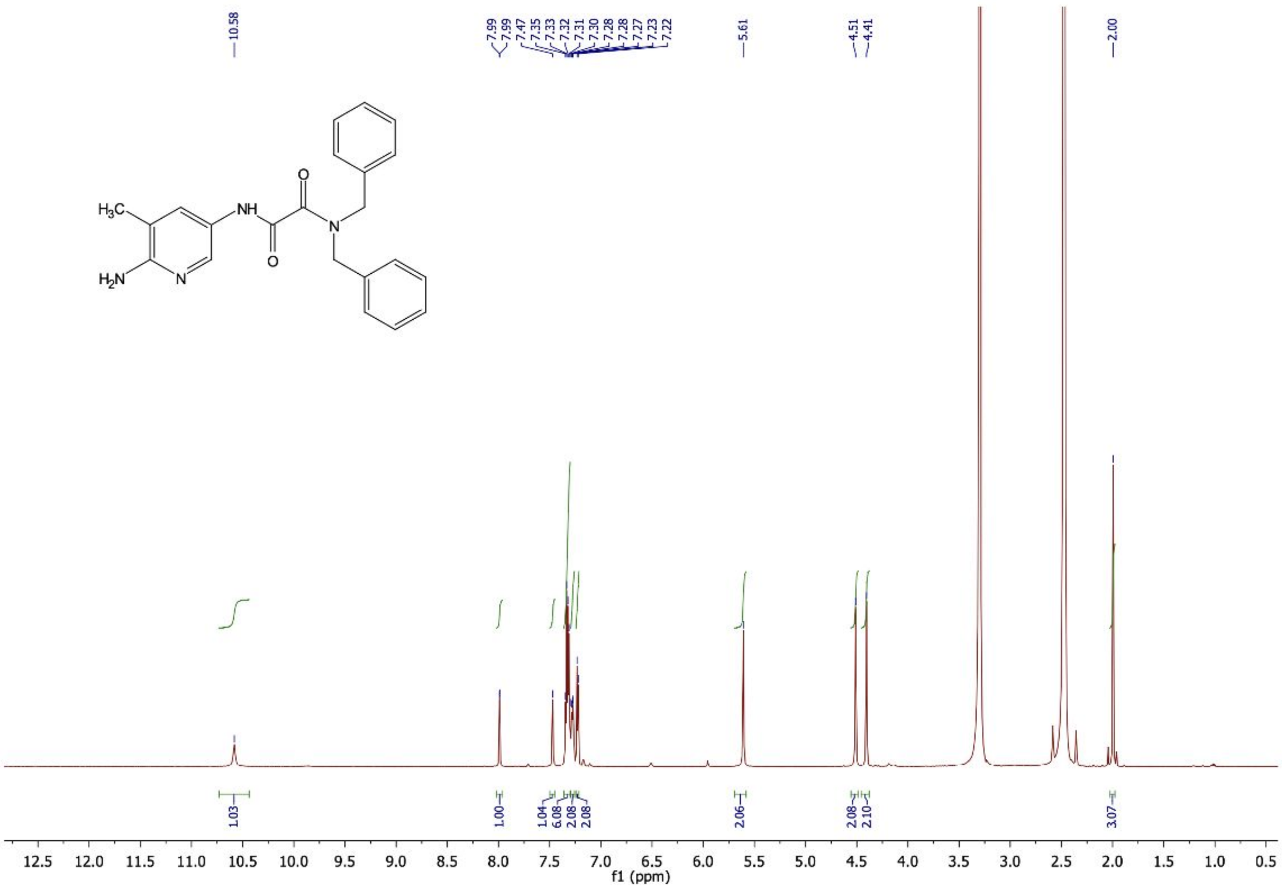

10.58
7.99
7.99
7.47
7.35
7.33
7.32
7.31
7.30
7.28
7.28
7.27
7.23
7.22
5.61
4.51
4.41
2.00
H3C
NH
O
O
N
H2N
N
1.03
1.00
1.04
6.08
2.08
2.08
2.06
2.08
2.10
3.07
12.5
12.0
11.5
11.0
10.5
10.0
9.5
9.0
8.5
8.0
7.5
7.0
6.5
6.0
5.5
5.0
4.5
4.0
3.5
3.0
2.5
2.0
1.5
1.0
0.5
f1 (ppm)

MaxPeak: 100.00%
Ret_Time: 2.486 min

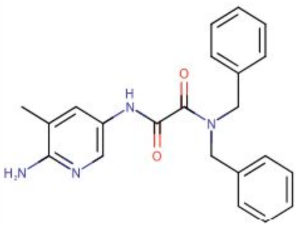

**Mol Wt 374.44**
**Exact Mass 374.2**

| # | Time | Area% |
|---|---|---|
| 1 | 2.486 | 100.00 |

H2345728

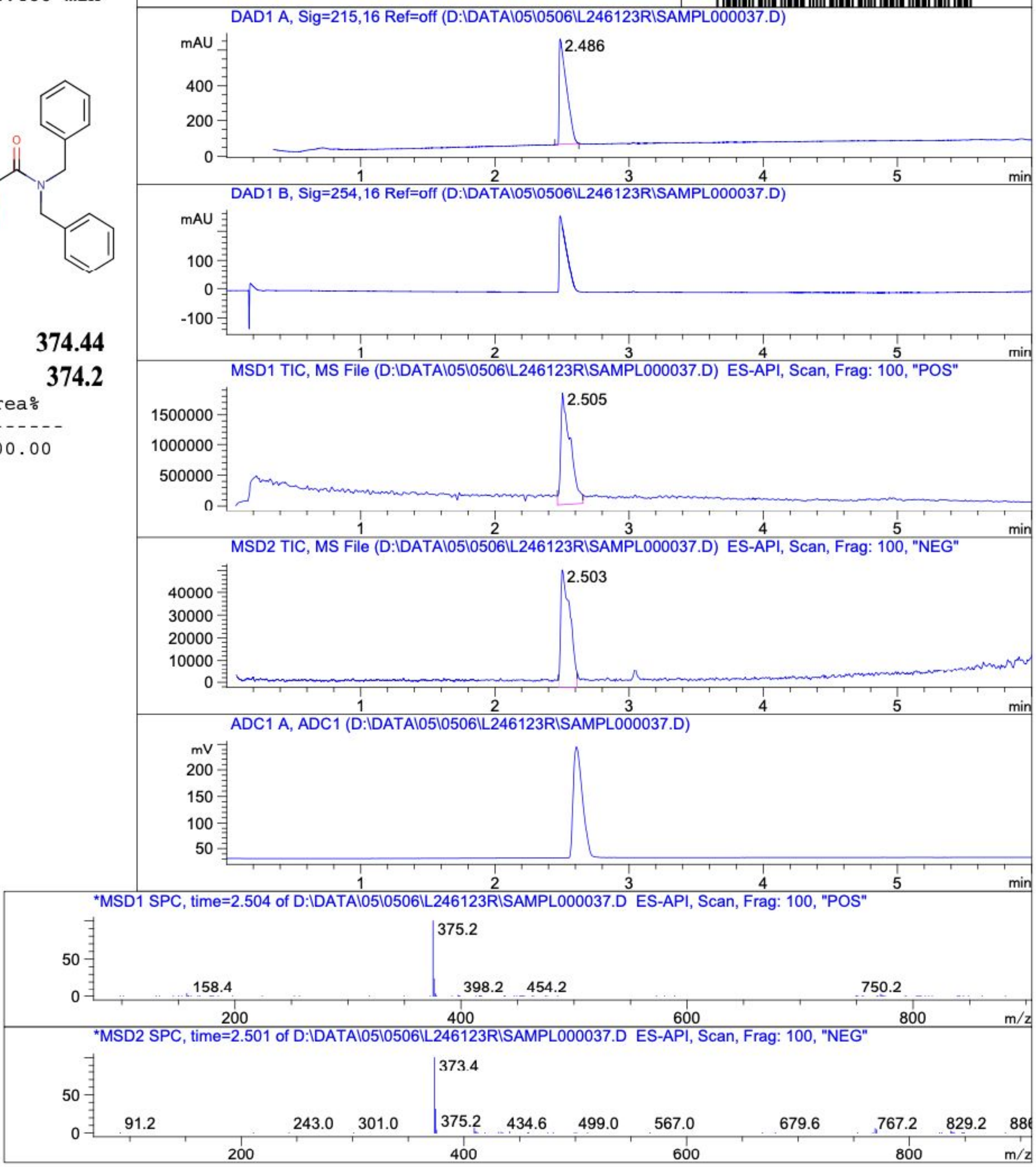


**COMPOUND 12**

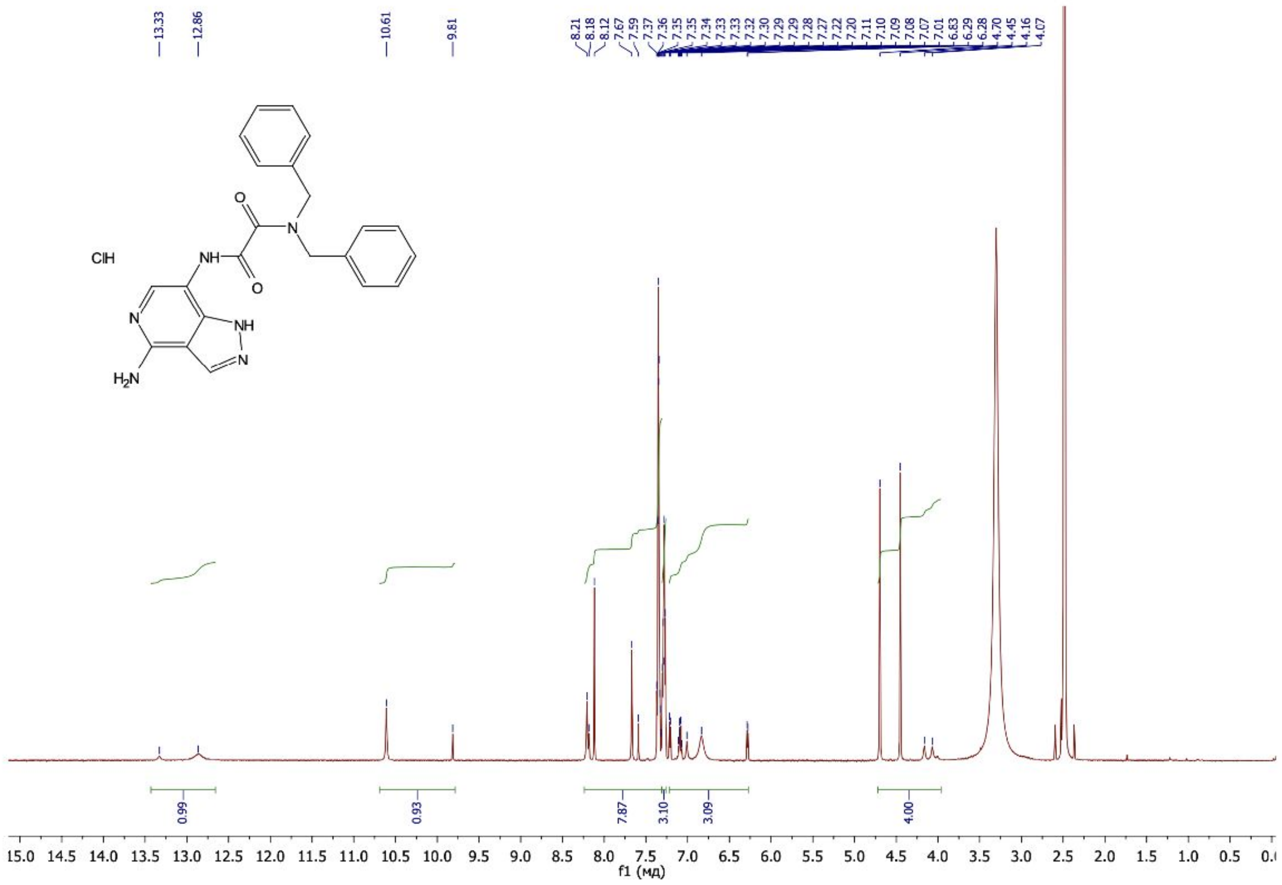

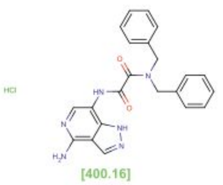


W314720$1

L407625F
LCMS12
6min_4-6x30_1-5_V.M
15:27 28.08.2021
MaxPeak: 66.6%

| # | RT | DAD1A | DAD1B | MSD1 | MSD2 | ELSD | MSD1 ions | MSD1 rt | MSD2 ions | MSD2 rt | Info |
|---|---|---|---|---|---|---|---|---|---|---|---|
| 1 | 2.014 | 2.4% | 7.0% | --- | --- | --- | --- | --- | --- | --- | |
| 2 | 2.514 | 97.6% | 93.0% | --- | 1.7% | --- | --- | --- | 401.0(100) | 2.512 | |
| 3 | 2.527 | --- | --- | 100.0% | 98.3% | --- | 401.2(100) | 2.527 | 399.2(100) | 2.527 | P +H+,P NEG |
| 4 | 2.612 | --- | --- | --- | --- | 100.0% | --- | --- | --- | --- | |

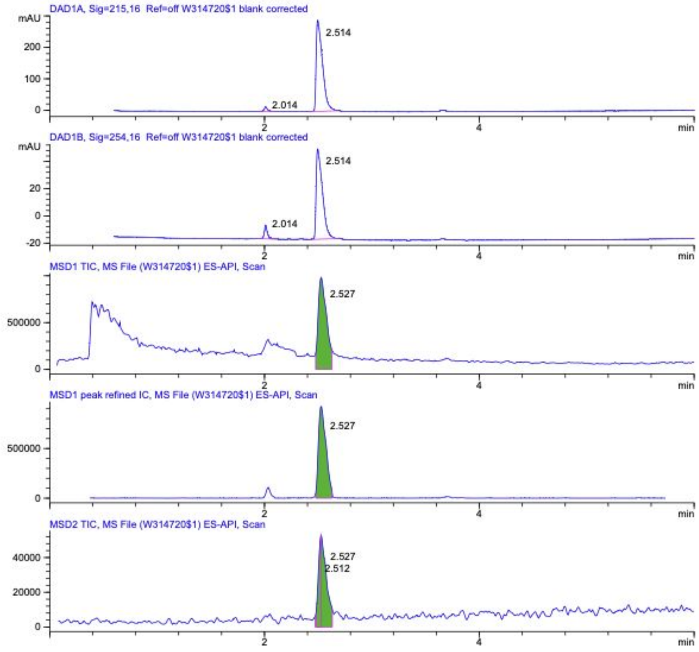




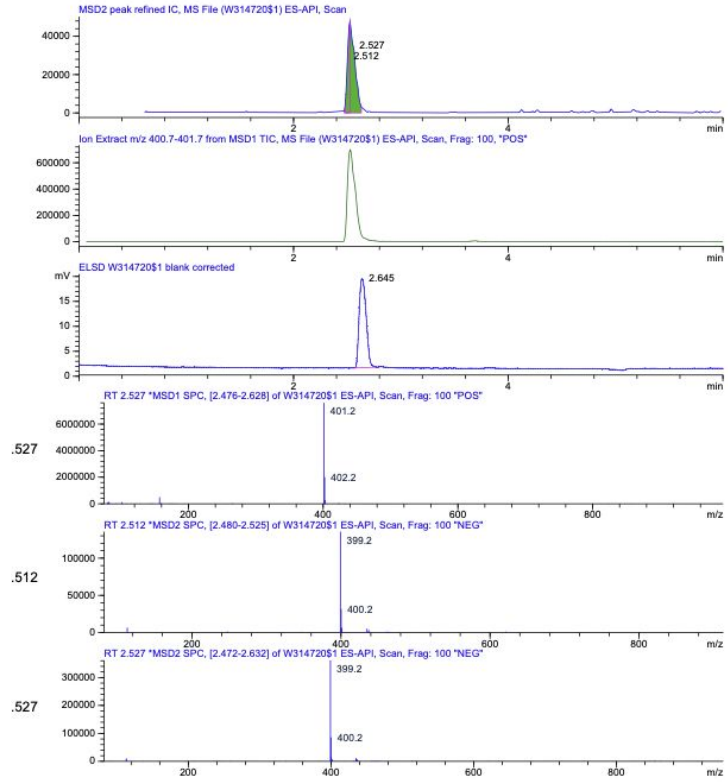

**COMPOUND 13**

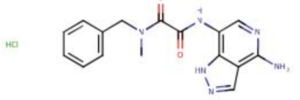


**Mol Wt** 360.8

**Exact Mass** 324.14

| # | Time | Area% |
|---|---|---|
| 1 | 1.355 | 100.00 |

RT 1.377

RT 1.370

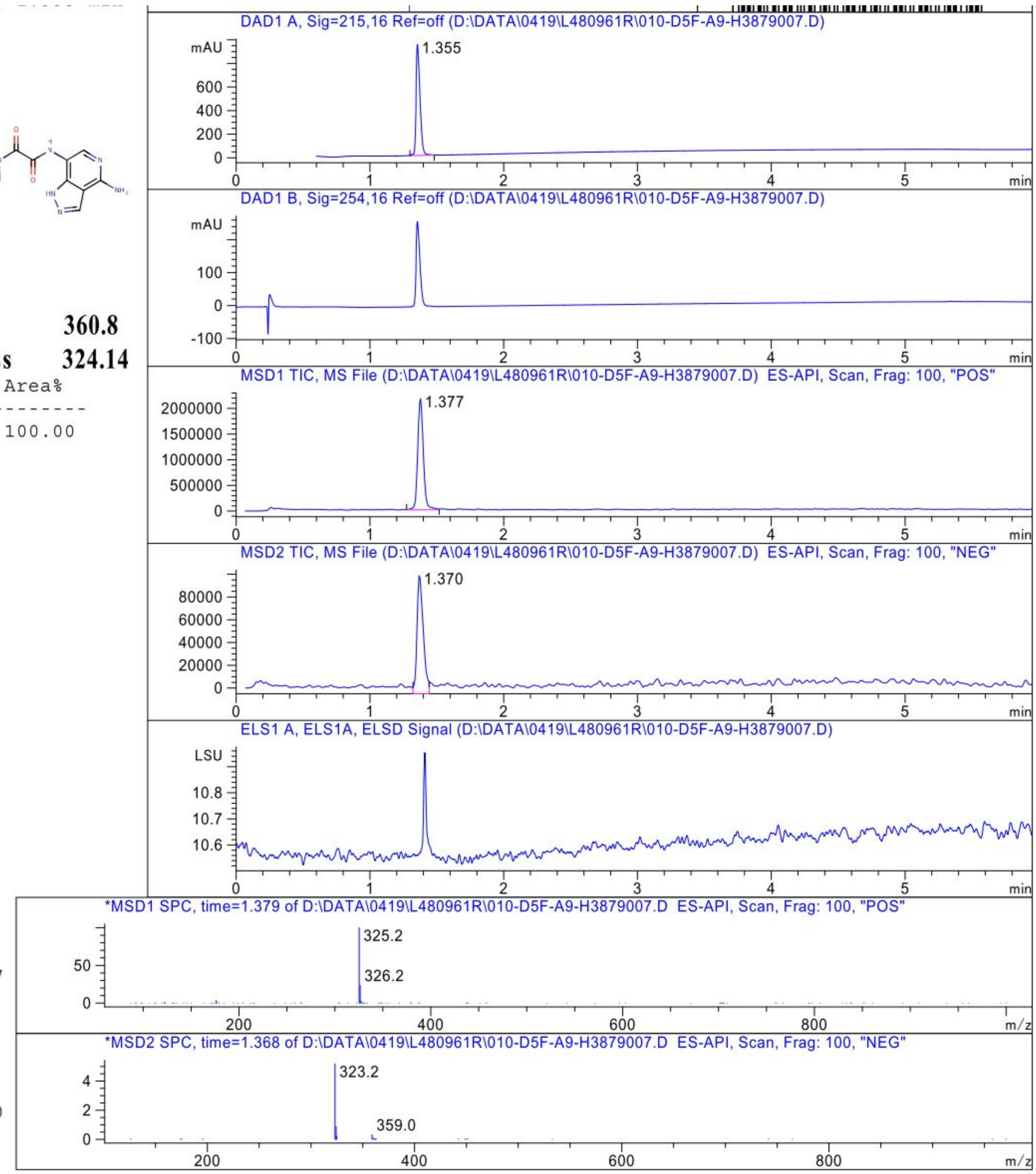

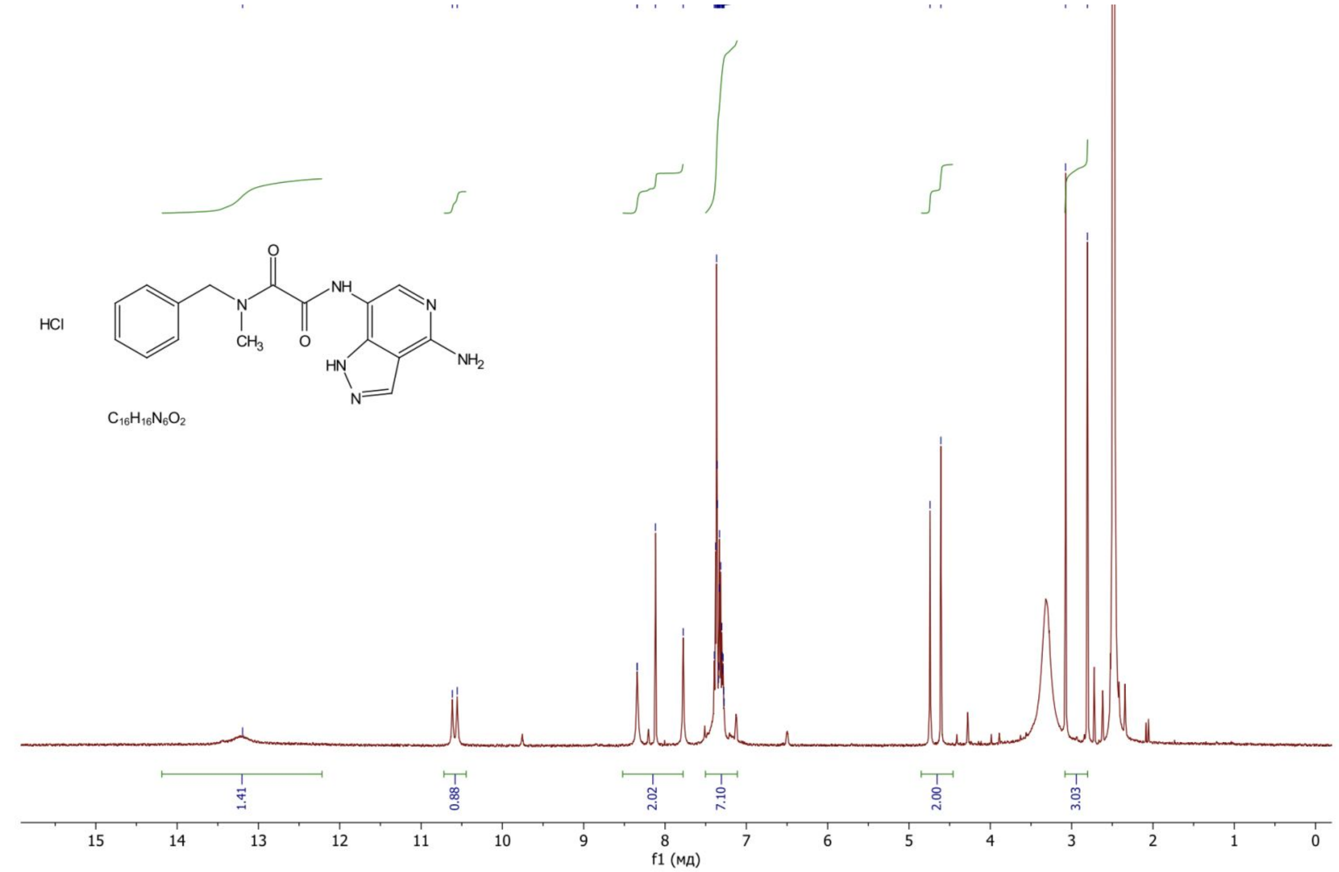
HCl
$C_{16}H_{16}N_6O_2$
1.41
0.88
2.02
7.10
2.00
3.03
f1 (мд)

**COMPOUND 14**

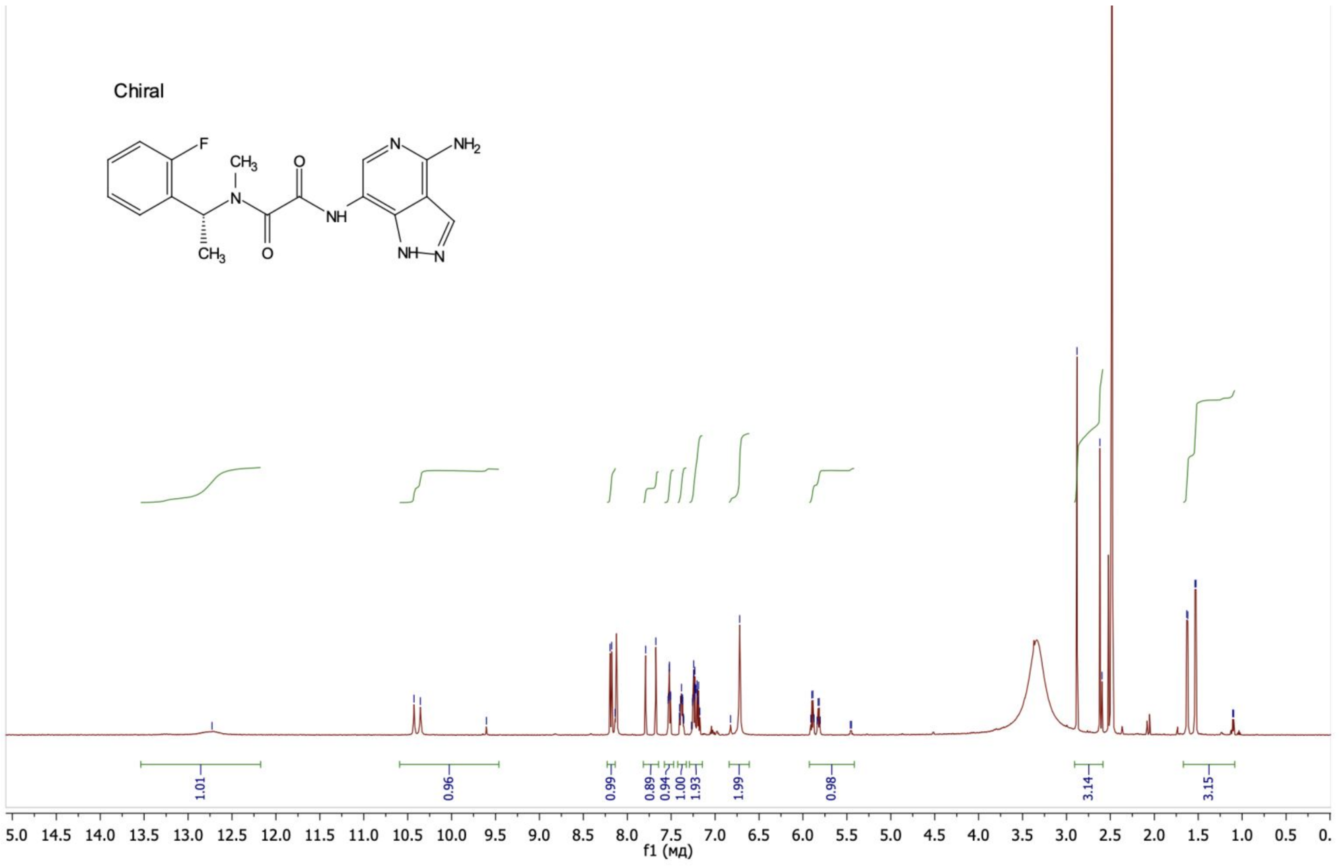
Chiral
1.01
0.96
0.99
0.89
0.94
1.00
1.93
1.99
0.98
3.14
3.15
f1 (мд)

**COMPOUND 15**

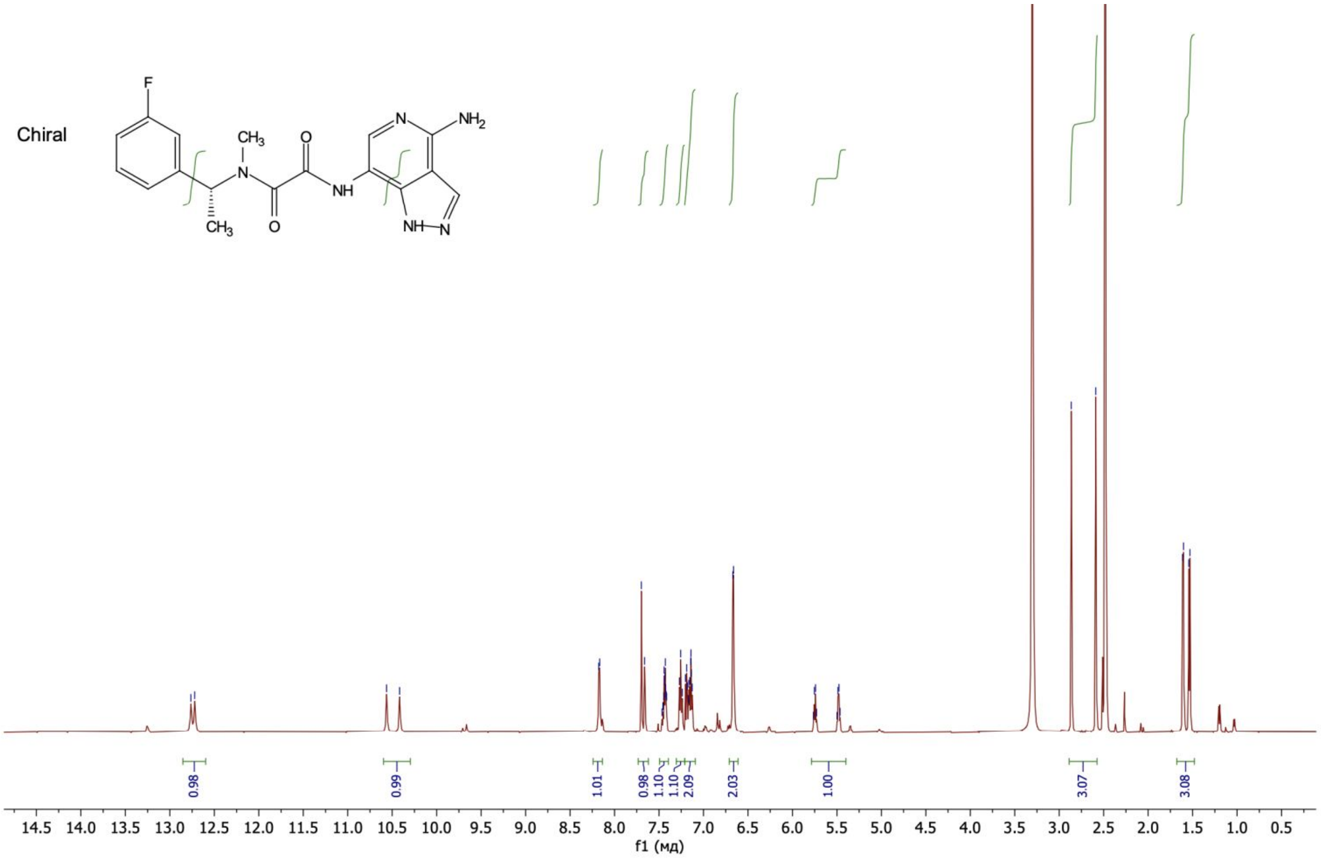

Chiral
F
CH3
O
N
NH2
N
NH
O
NH-N
CH3
0.98
0.99
1.01
0.98
1.10
1.10
2.09
2.03
1.00
3.07
3.08
14.5 14.0 13.5 13.0 12.5 12.0 11.5 11.0 10.5 10.0 9.5 9.0 8.5 8.0 7.5 7.0 6.5 6.0 5.5 5.0 4.5 4.0 3.5 3.0 2.5 2.0 1.5 1.0 0.5
f1 (мд)

MaxPeak: 100.00%
Ret_Time: 1.128 min

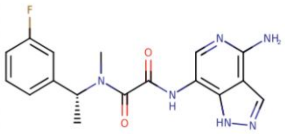

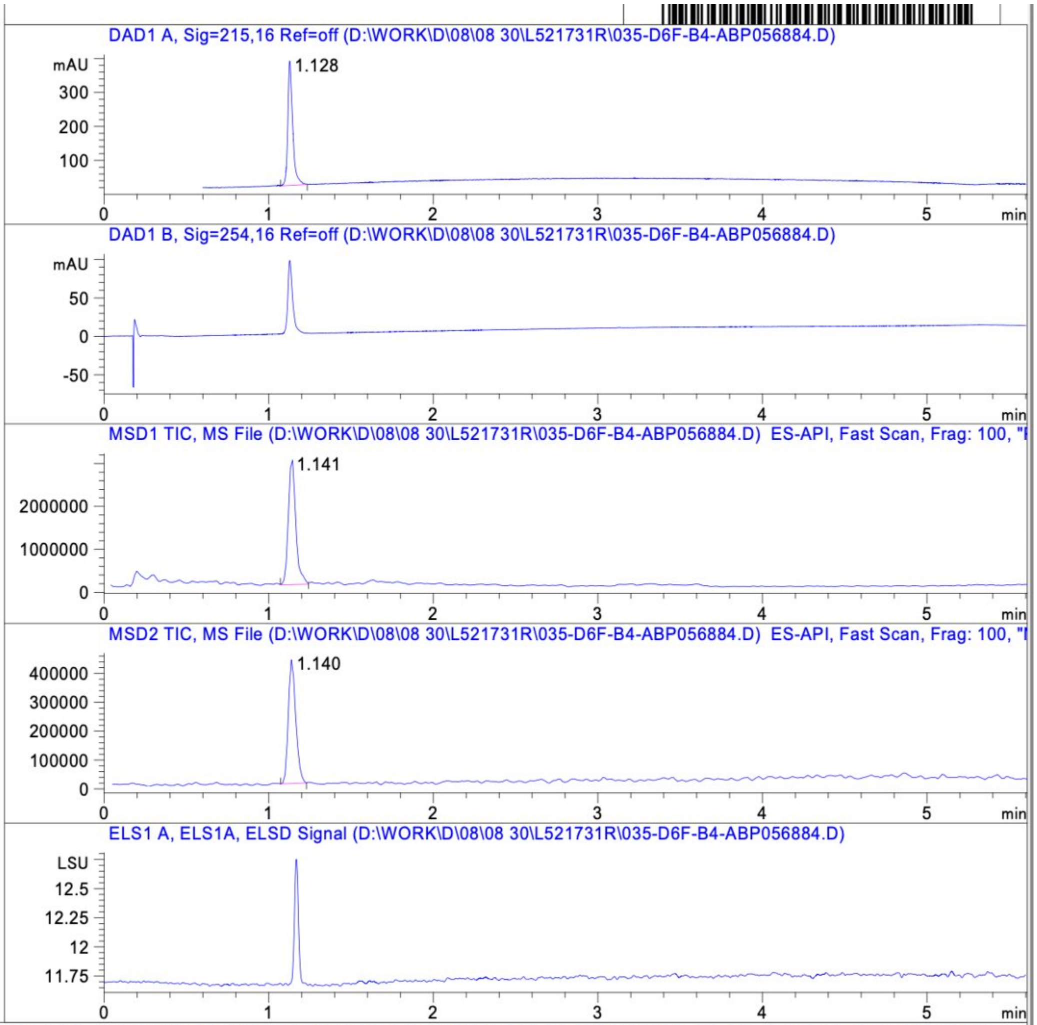


**Mol Wt** 356.35
**Exact Mass** 356.15

| # | Time | Area% |
|---|---|---|
| 1 | 1.128 | 100.00 |

RT 1.141

RT 1.140

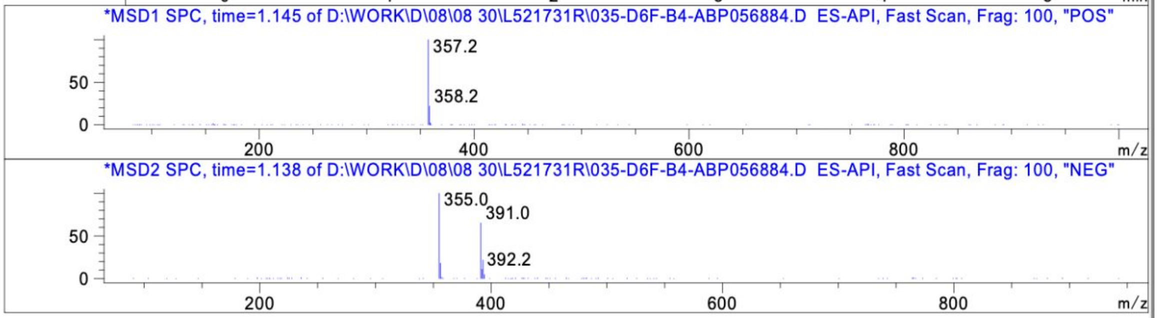


**COMPOUND 16**

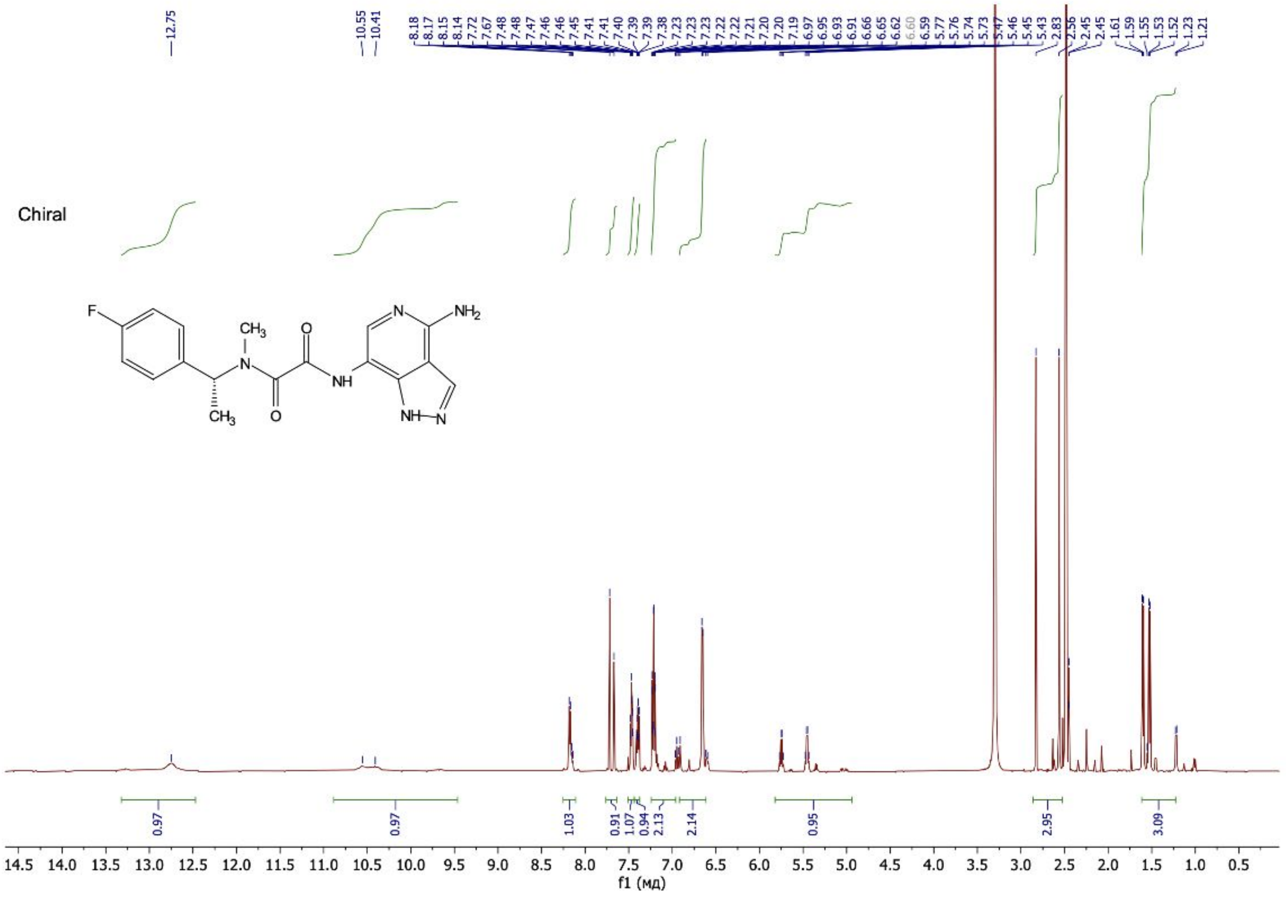


**COMPOUND 17**

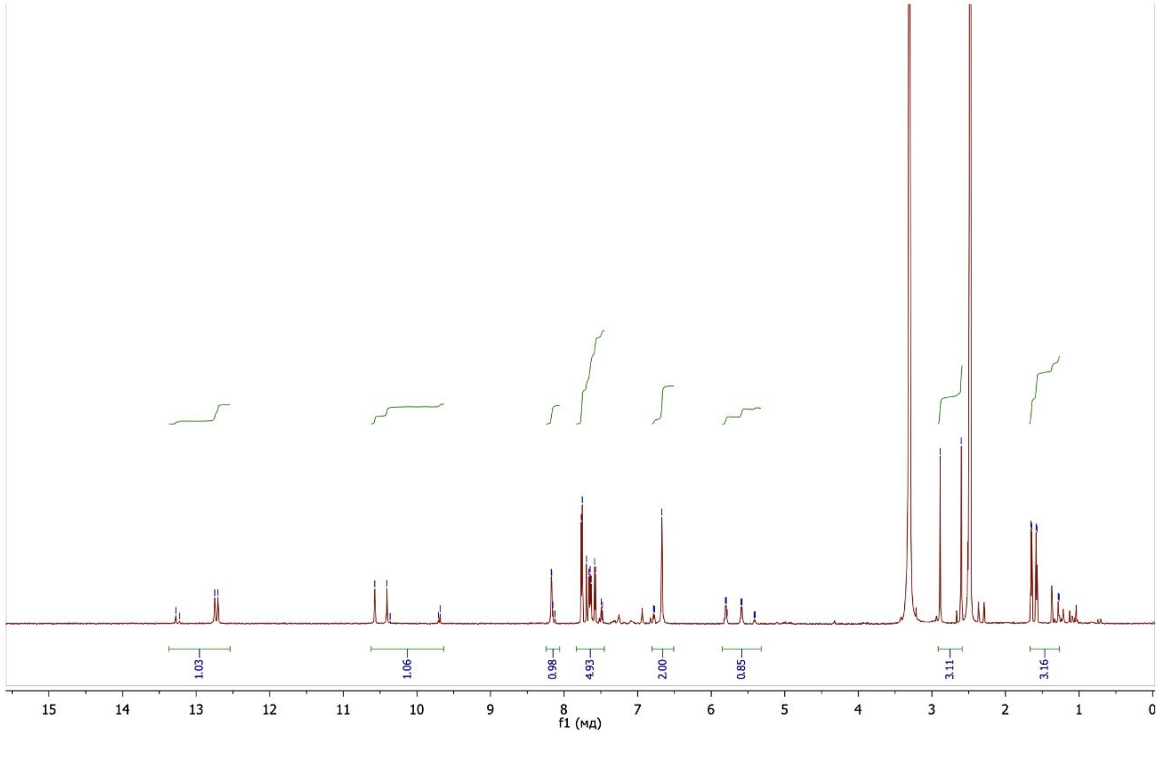

MaxPeak: 97.32%
Ret_Time: 2.265 min

**Mol Wt 406.36**
**Exact Mass 406.15**

| # | Time | Area% |
|---|---|---|
| 1 | 2.265 | 97.32 |
| 2 | 2.349 | 2.68 |

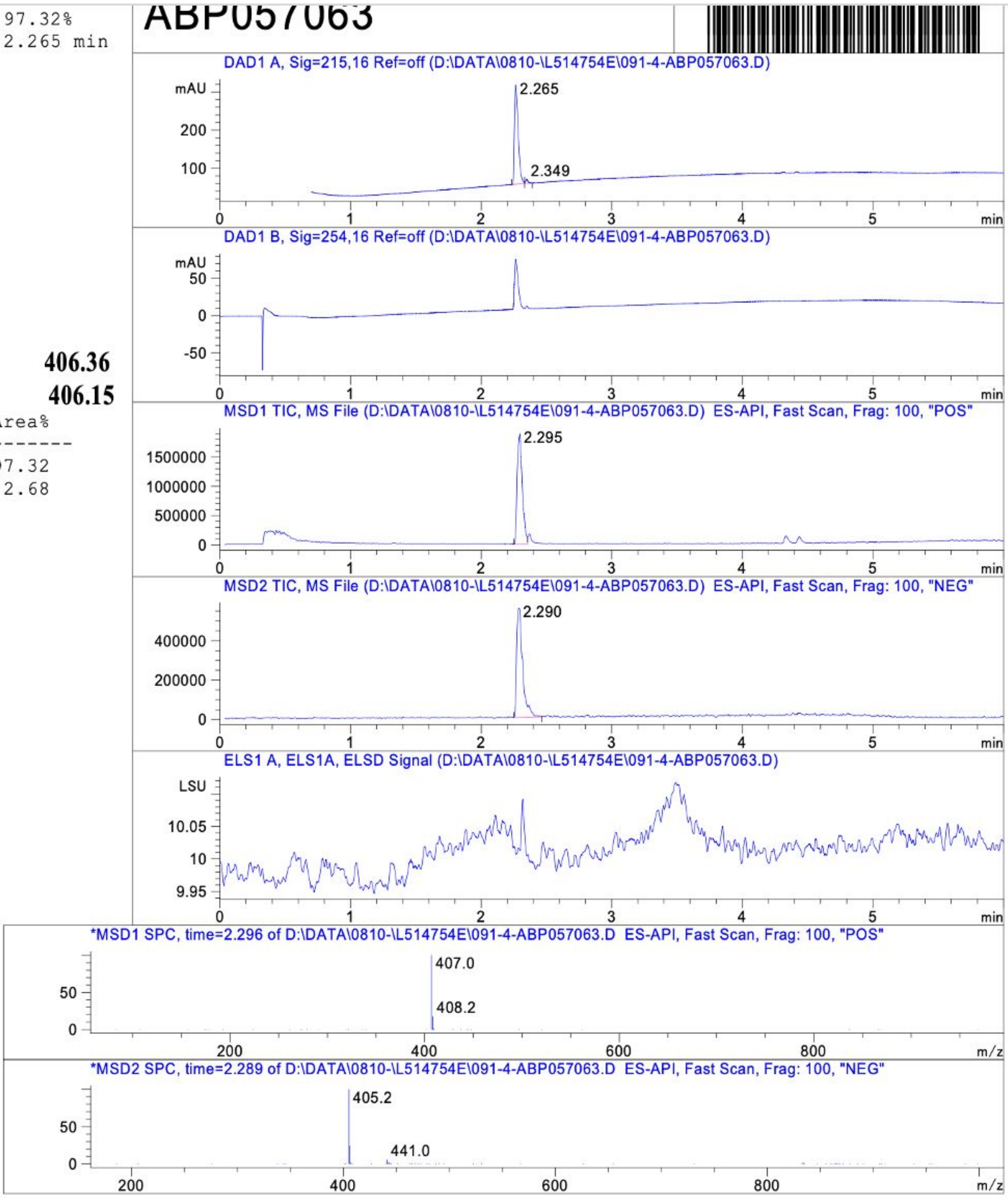


RT 2.295

RT 2.290

**COMPOUND 18**

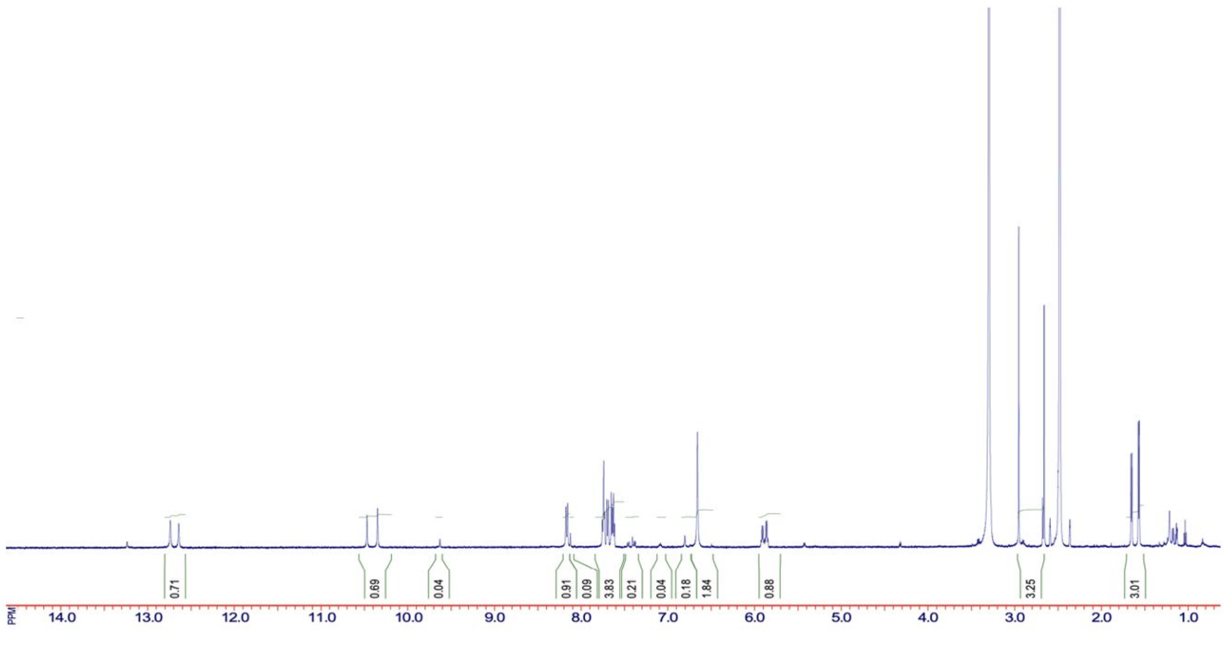

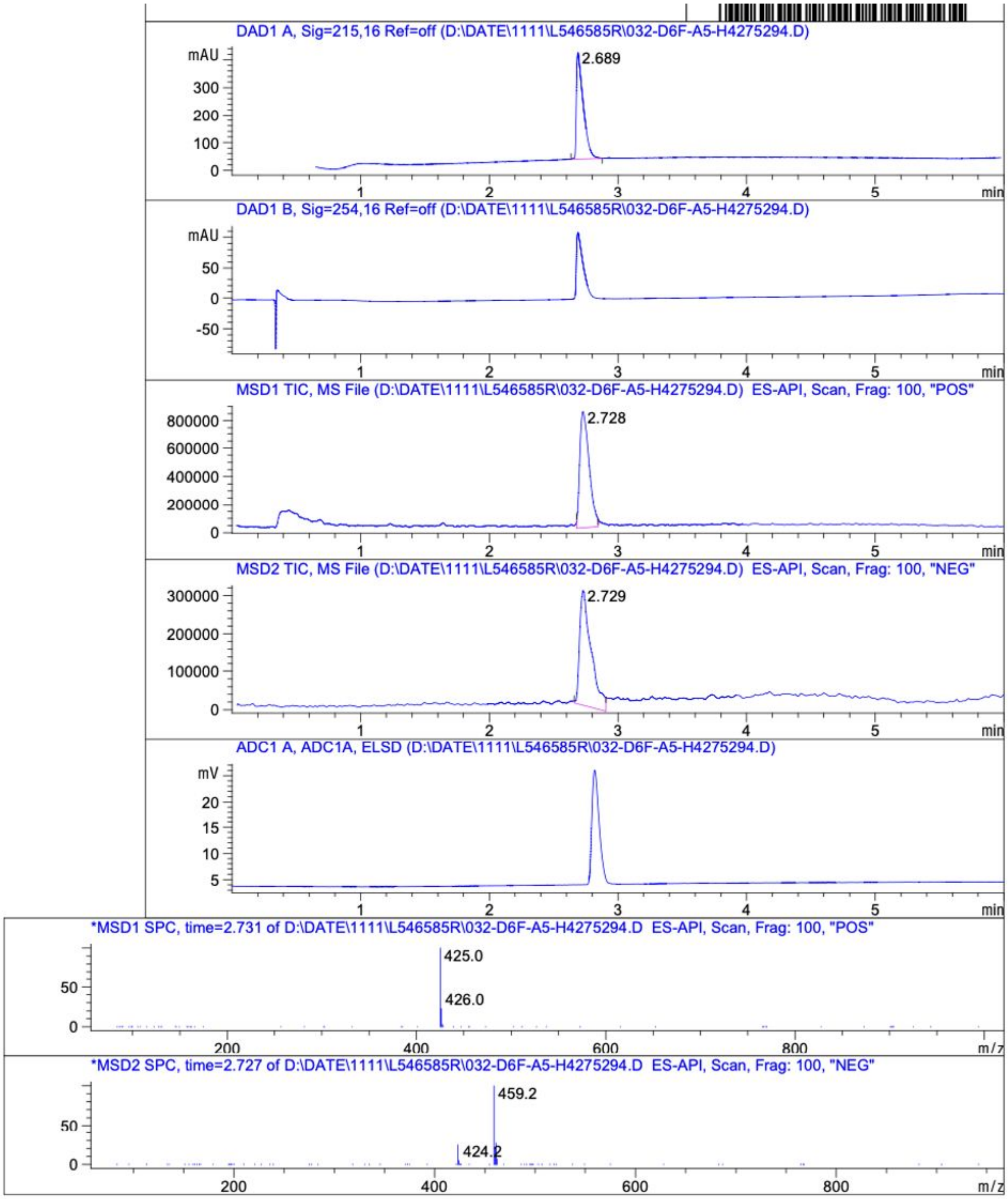


RT 2.728

RT 2.729

**COMPOUND 19**

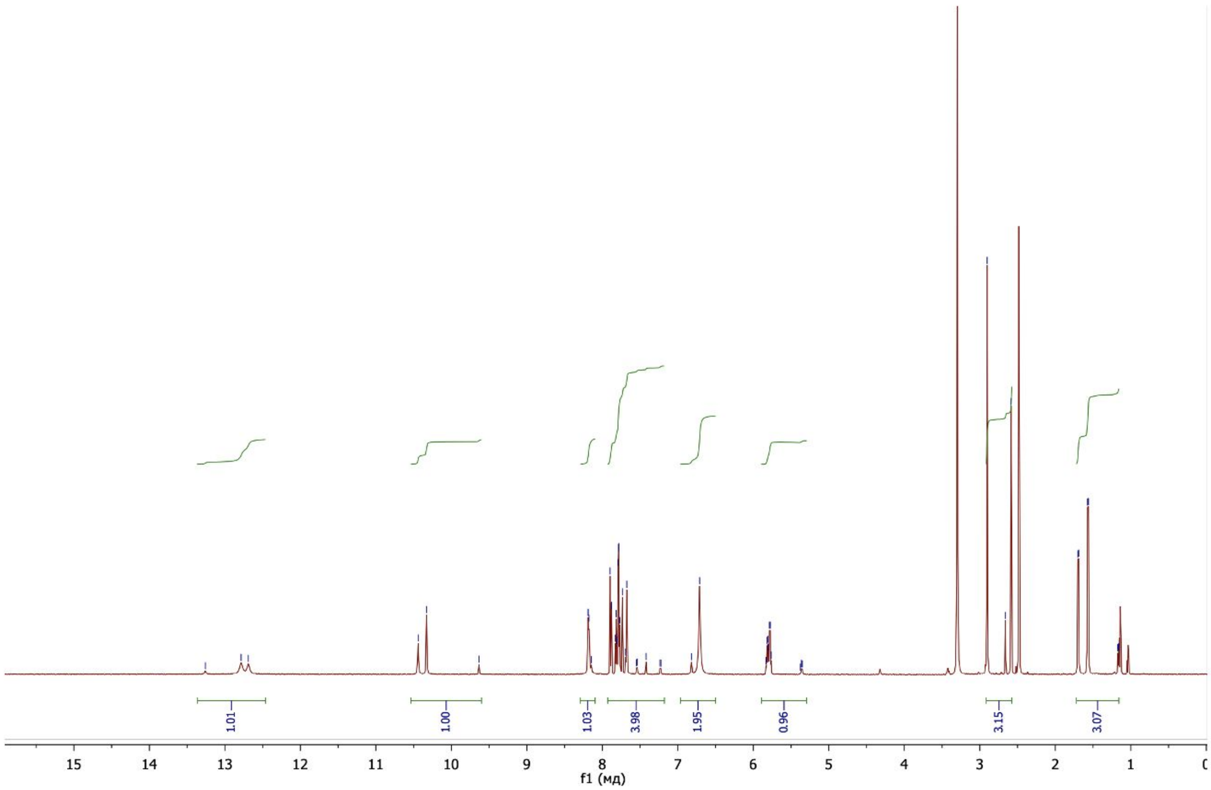
1.01
1.00
1.03
3.98
1.95
0.96
3.15
3.07
15
14
13
12
11
10
9
8
7
6
5
4
3
2
1
f1 (мд)

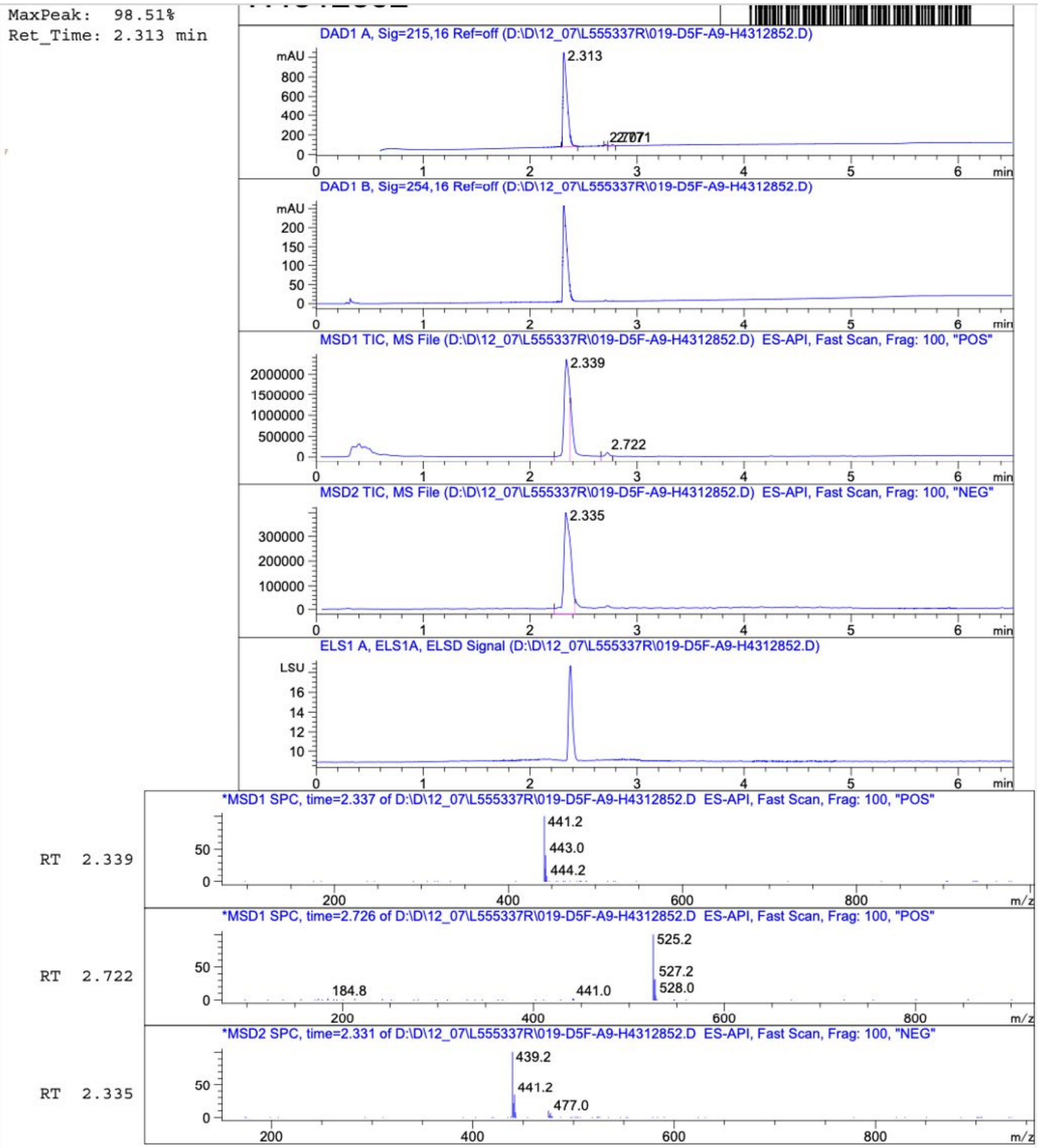
MaxPeak: 98.51%
Ret_Time: 2.313 min
DAD1 A, Sig=215,16 Ref=off (D:\D\12_07\L555337R\019-D5F-A9-H4312852.D)
DAD1 B, Sig=254,16 Ref=off (D:\D\12_07\L555337R\019-D5F-A9-H4312852.D)
MSD1 TIC, MS File (D:\D\12_07\L555337R\019-D5F-A9-H4312852.D) ES-API, Fast Scan, Frag: 100, "POS"
MSD2 TIC, MS File (D:\D\12_07\L555337R\019-D5F-A9-H4312852.D) ES-API, Fast Scan, Frag: 100, "NEG"
ELS1 A, ELS1A, ELSD Signal (D:\D\12_07\L555337R\019-D5F-A9-H4312852.D)
RT 2.339
*MSD1 SPC, time=2.337 of D:\D\12_07\L555337R\019-D5F-A9-H4312852.D ES-API, Fast Scan, Frag: 100, "POS"
RT 2.722
*MSD1 SPC, time=2.726 of D:\D\12_07\L555337R\019-D5F-A9-H4312852.D ES-API, Fast Scan, Frag: 100, "POS"
RT 2.335
*MSD2 SPC, time=2.331 of D:\D\12_07\L555337R\019-D5F-A9-H4312852.D ES-API, Fast Scan, Frag: 100, "NEG"

**COMPOUND 20**

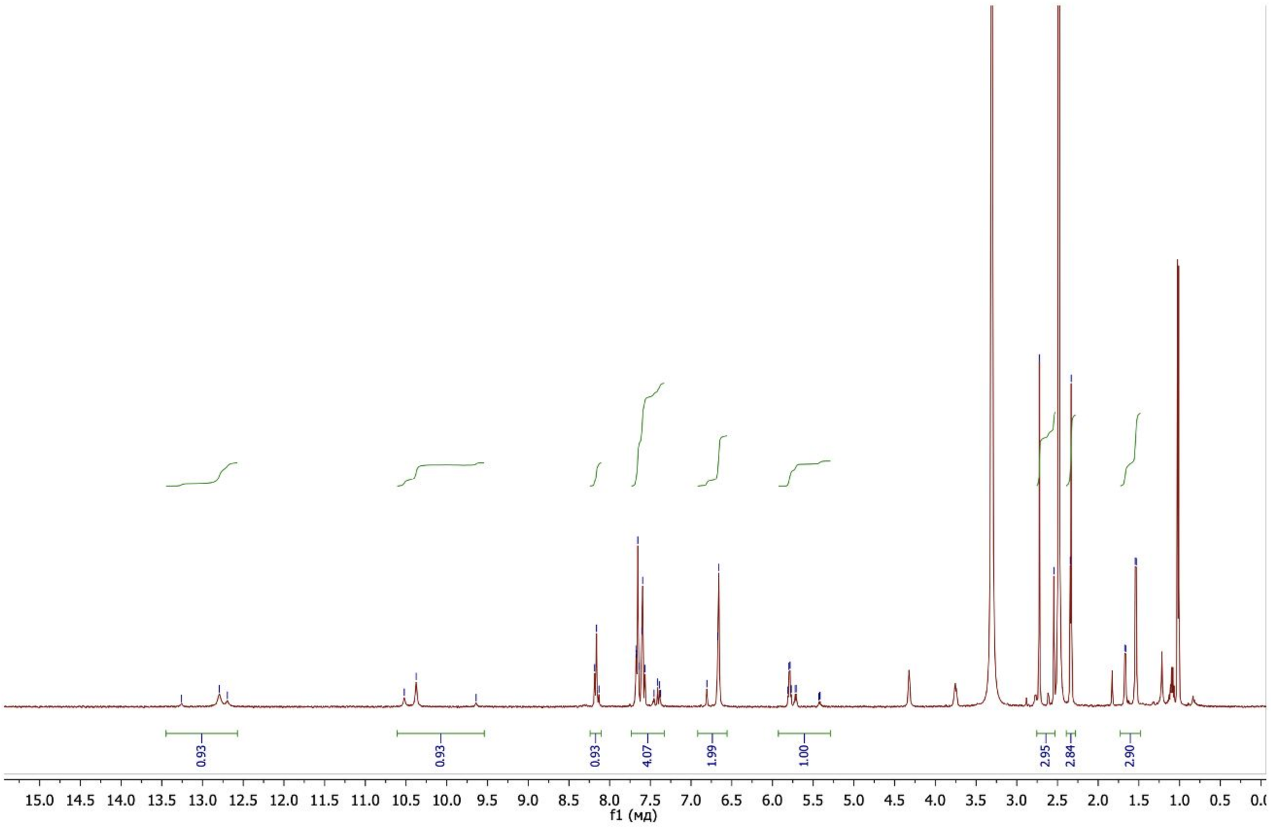
0.93
0.93
0.93
4.07
1.99
1.00
2.95
2.84
2.90
15.0 14.5 14.0 13.5 13.0 12.5 12.0 11.5 11.0 10.5 10.0 9.5 9.0 8.5 8.0 7.5 7.0 6.5 6.0 5.5 5.0 4.5 4.0 3.5 3.0 2.5 2.0 1.5 1.0 0.5
f1 (мд)

MaxPeak: 98.18%
Ret Time: 2.599 min

RT 2.614

RT 3.070

RT 2.613

RT 3.060

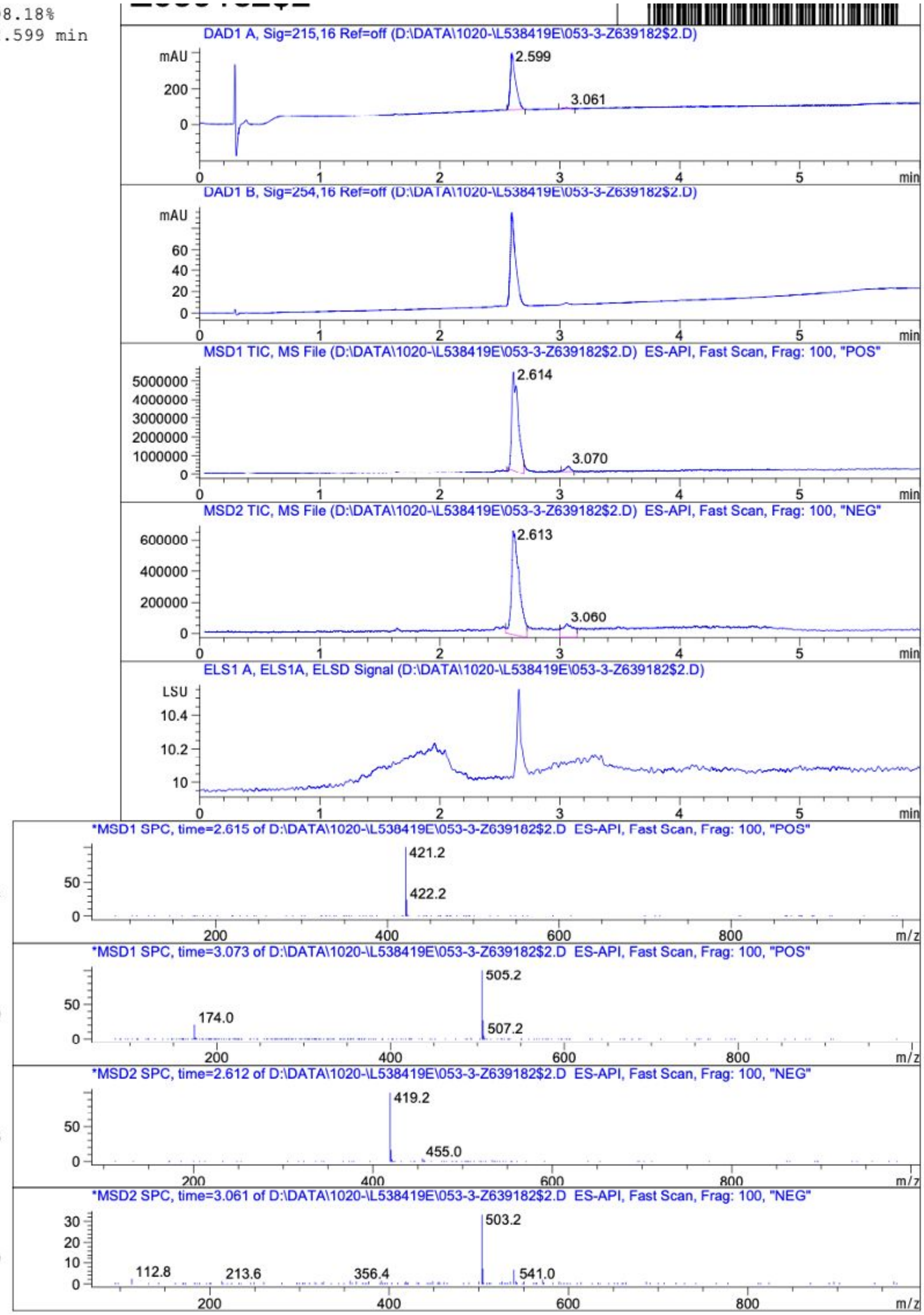


**COMPOUND 21**

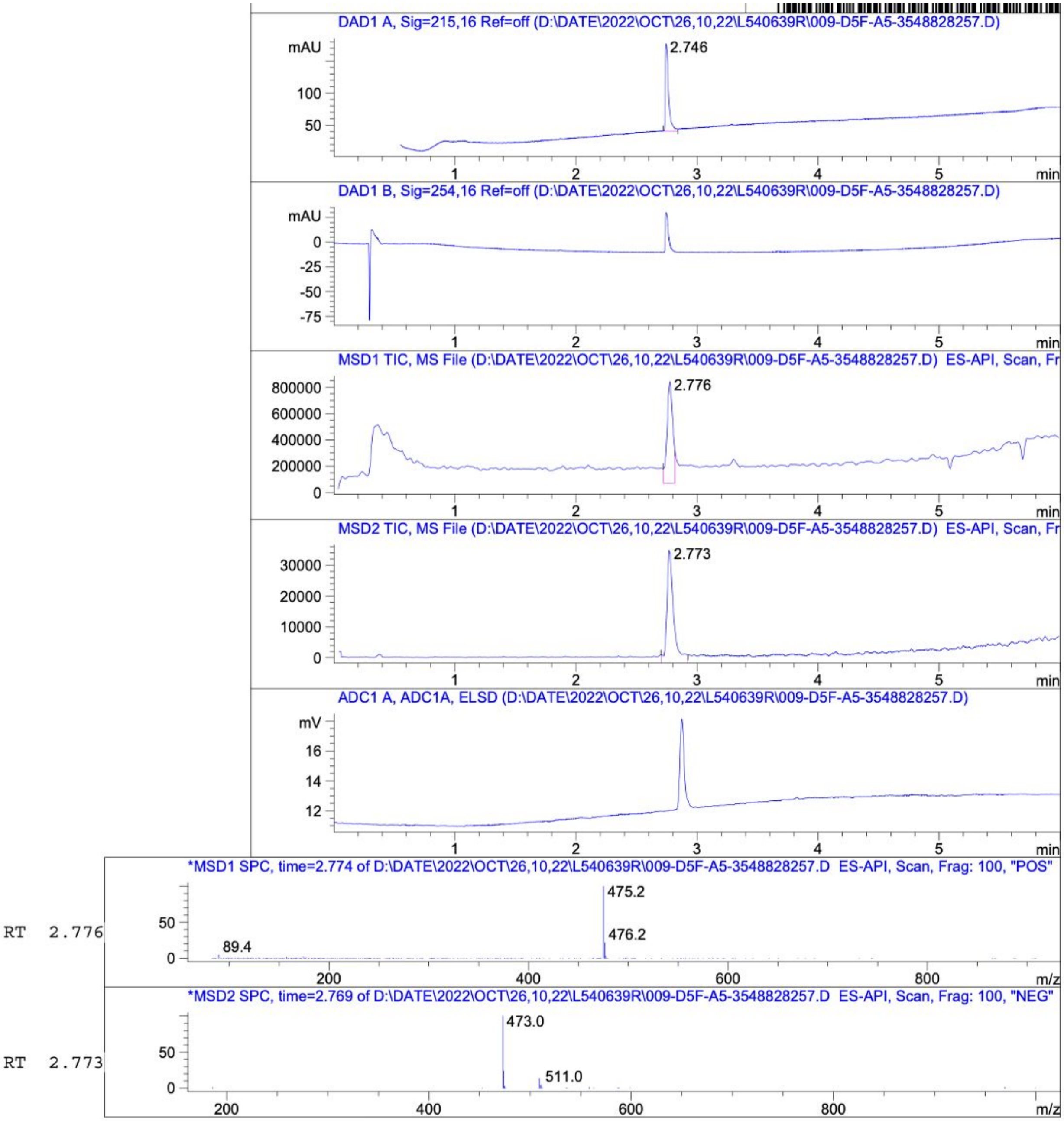


**TNG456**

**1H NMR TNG456**

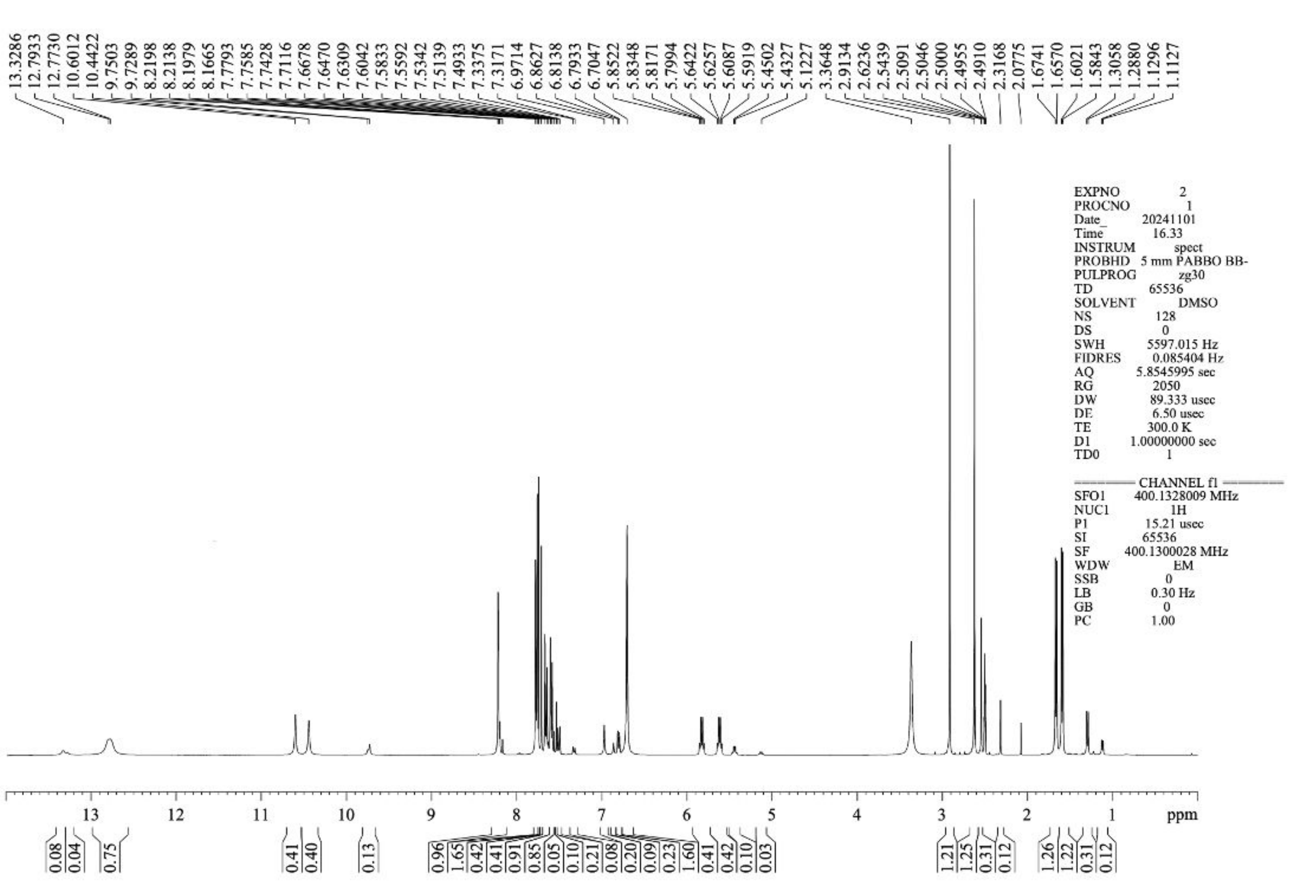

**13C NMR – TNG456**

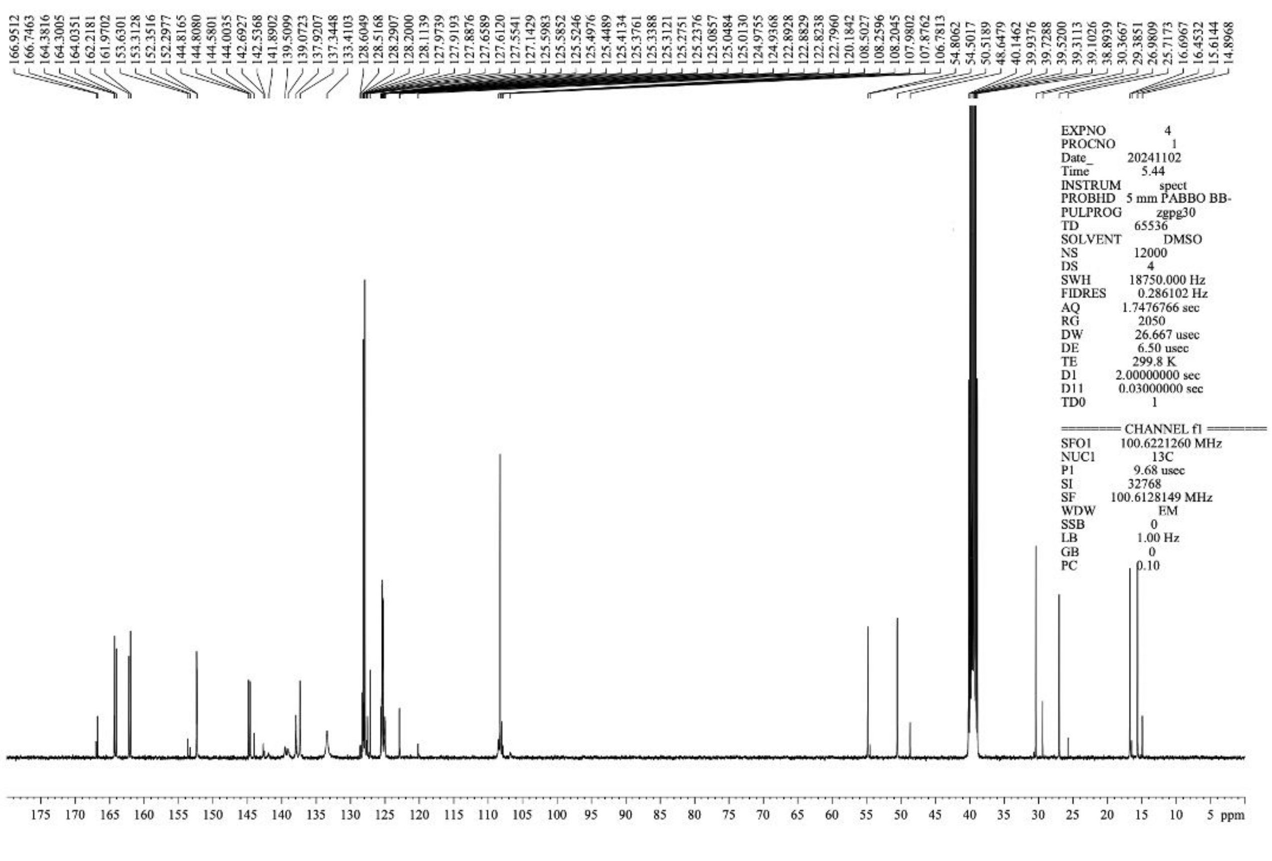

**HIGH RESOLUTION MASS SPEC – TNG456**

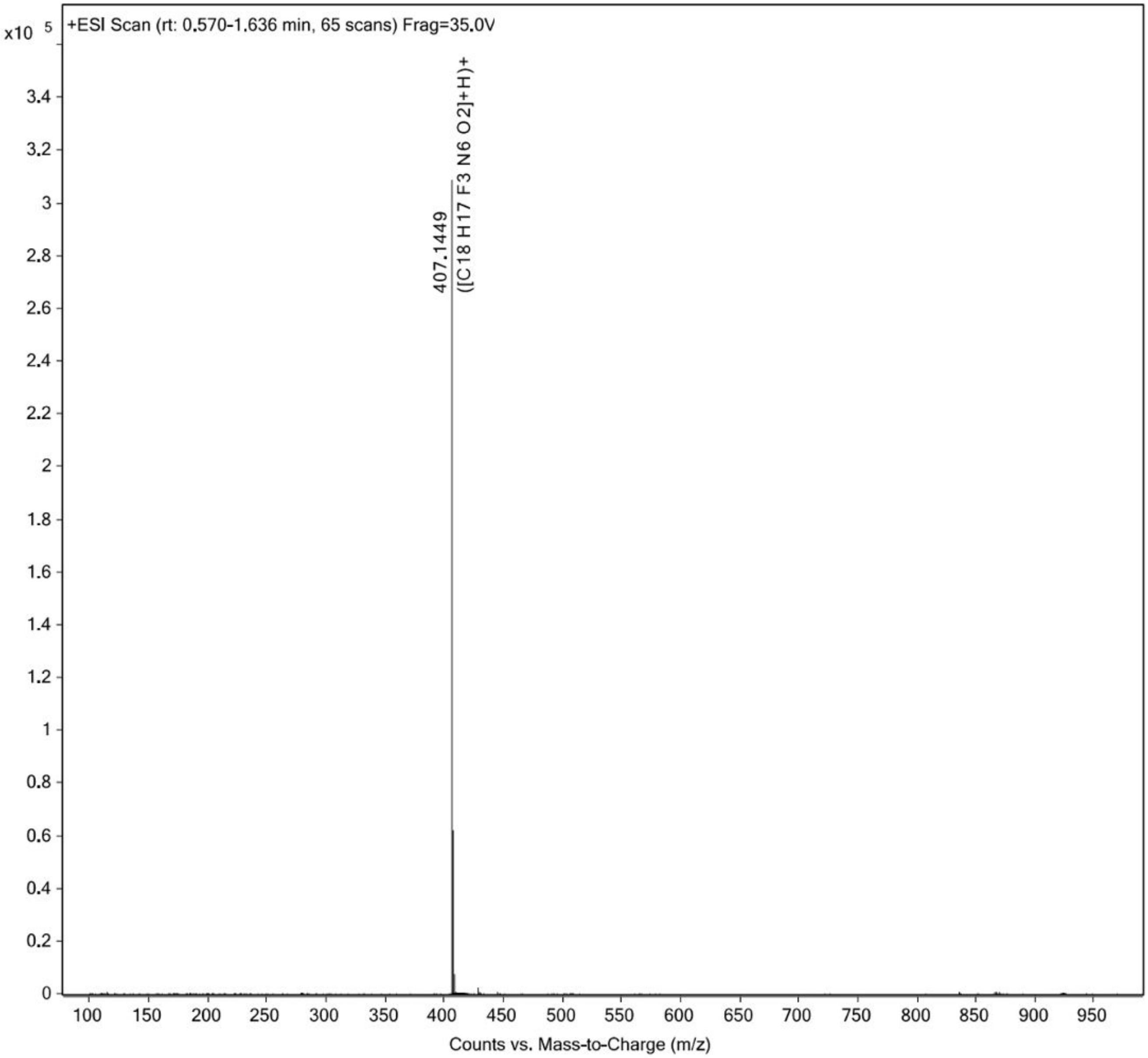


**HPLC – TNG456**

Method Information

Instrument:Shimadzu LC-20AD
Mobile Phase:A:0.04%TFA in H2O
Mobile Phase:B:0.02%TFA in ACN
Flow :1.2000
Column Temperature:40 C

| Time | Unit | Command | Value | Comment |
|---|---|---|---|---|
| 0.01 | Pumps | B.Conc | 10 | |
| 16.00 | Pumps | B.Conc | 80 | |
| 19.00 | Pumps | B.Conc | 100 | |
| 19.00 | Pumps | T.Flow | 1.2 | |
| 19.01 | Pumps | B.Conc | 10 | |
| 19.01 | Pumps | T.Flow | 1.2 | |
| 22.00 | Controller | Stop | | |

Chromatogram

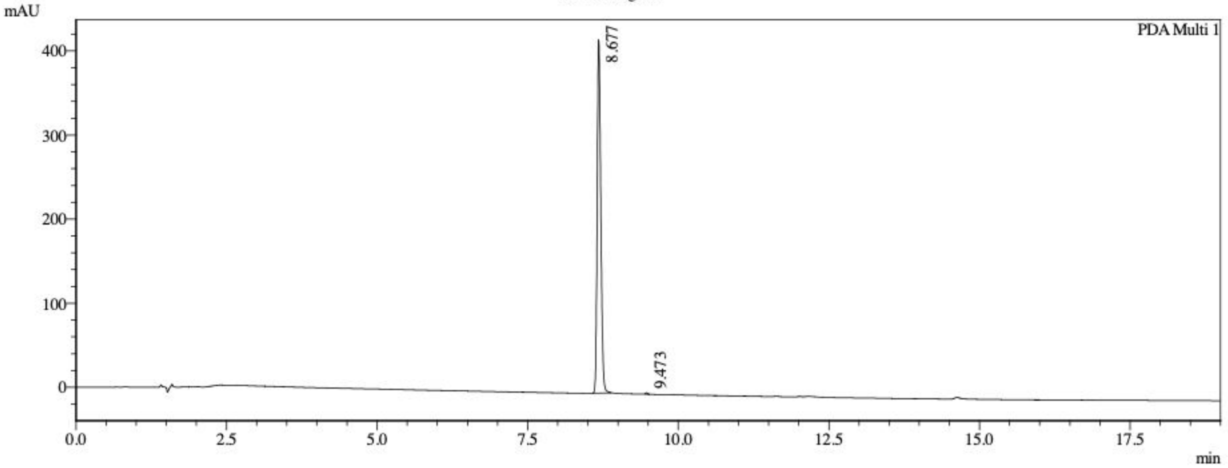


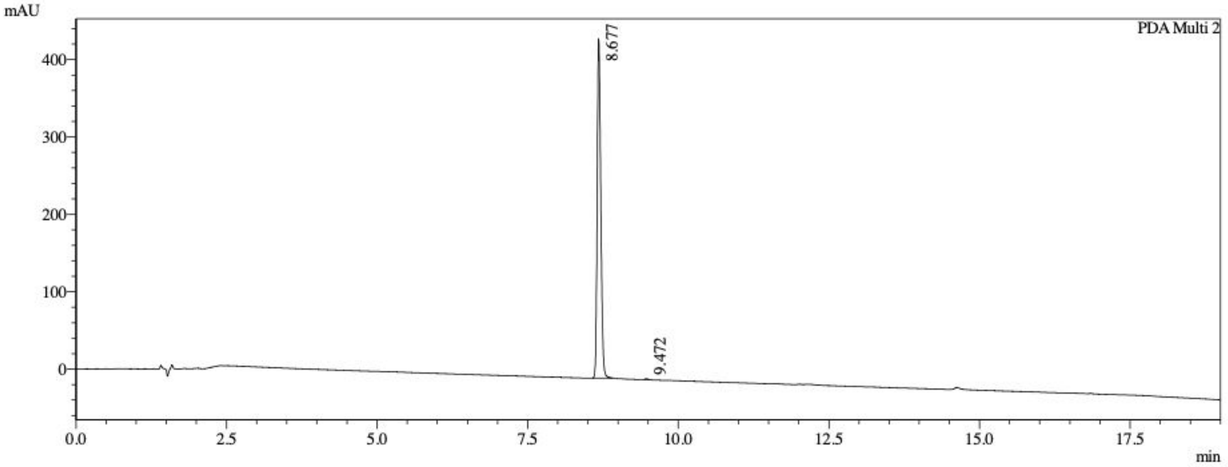

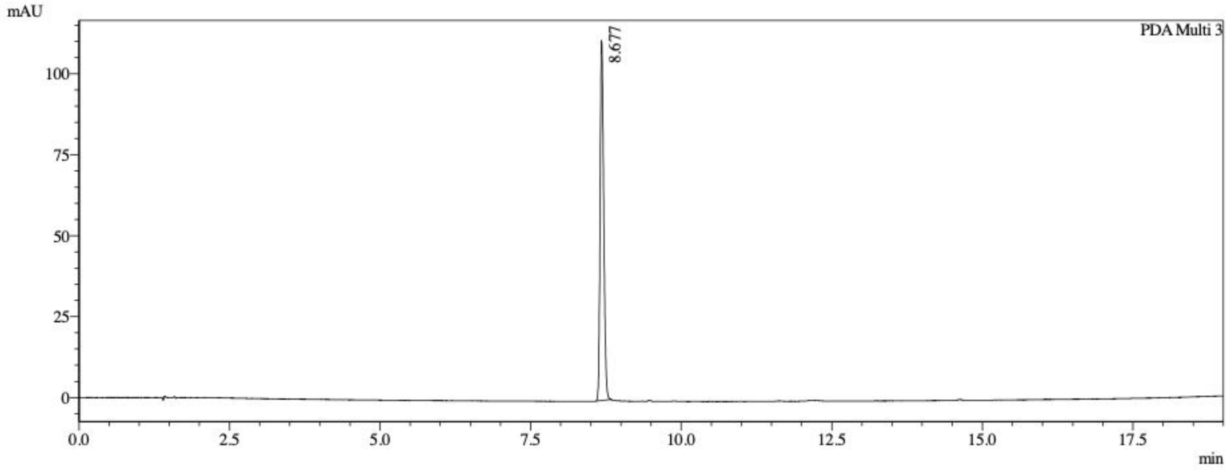


1 PDA Multi 1 / 220nm 4nm
2 PDA Multi 2 / 215nm 4nm
3 PDA Multi 3 / 254nm 4nm

Integration Result

PDA Ch1 220nm 4nm

| Peak# | Ret. Time | Height | Height % | USP Width | Area | Area % |
|---|---|---|---|---|---|---|
| 1 | 8.677 | 420175 | 99.694 | 0.112 | 1744512 | 99.817 |
| 2 | 9.473 | 1288 | 0.306 | 0.071 | 3202 | 0.183 |

PDA Ch2 215nm 4nm

| Peak# | Ret. Time | Height | Height % | USP Width | Area | Area % |
|---|---|---|---|---|---|---|
| 1 | 8.677 | 438383 | 99.698 | 0.112 | 1819433 | 99.810 |
| 2 | 9.472 | 1328 | 0.302 | 0.074 | 3459 | 0.190 |

PDA Ch3 254nm 4nm

| Peak# | Ret. Time | Height | Height % | USP Width | Area | Area % |
|---|---|---|---|---|---|---|
| 1 | 8.677 | 111130 | 100.000 | 0.112 | 458294 | 100.000 |

**LCMS – TNG456**

Method Information

Instrument: Shimadzu LC-20ADXR MSD: LCMS-2020
Mobile Phase A:0.04% TFA in H2O
Mobile Phase B:0.02% TFA in ACN
Total Flow : 0.5000 mL/min
Oven Temperature: 40 C

| Time | Module | Command | Value | Comment |
|---|---|---|---|---|
| 0.01 | Pumps | B.Conc | 10 | |
| 8.00 | Pumps | B.Conc | 80 | |
| 10.00 | Pumps | B.Conc | 80 | |
| 10.01 | Pumps | B.Conc | 10 | |
| 13.00 | Pumps | B.Conc | 10 | |
| 13.00 | Controller | Stop | | |

<<MIC table>>
<MS>
Page1
Added m/z Range

| No. | Start m/z | End m/z |
|---|---|---|
| 1 | 608.8000 | 610.8000 |

Chromatogram

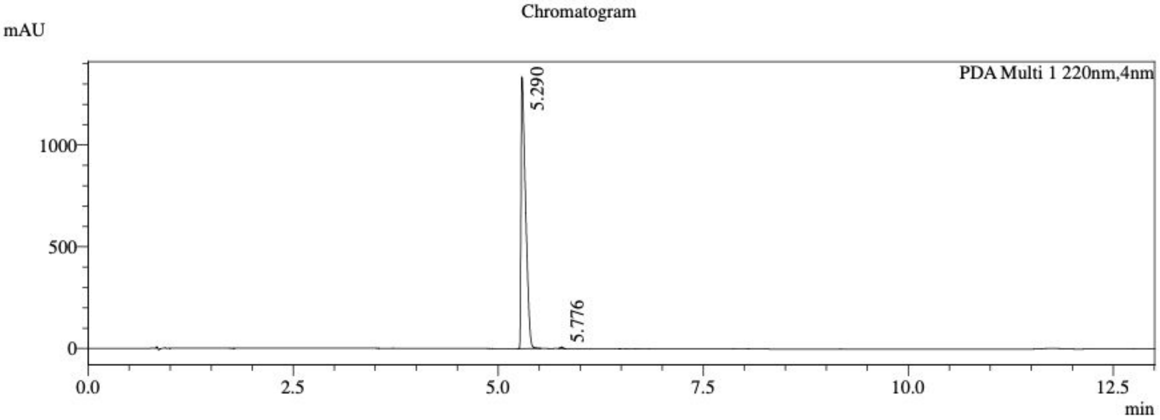


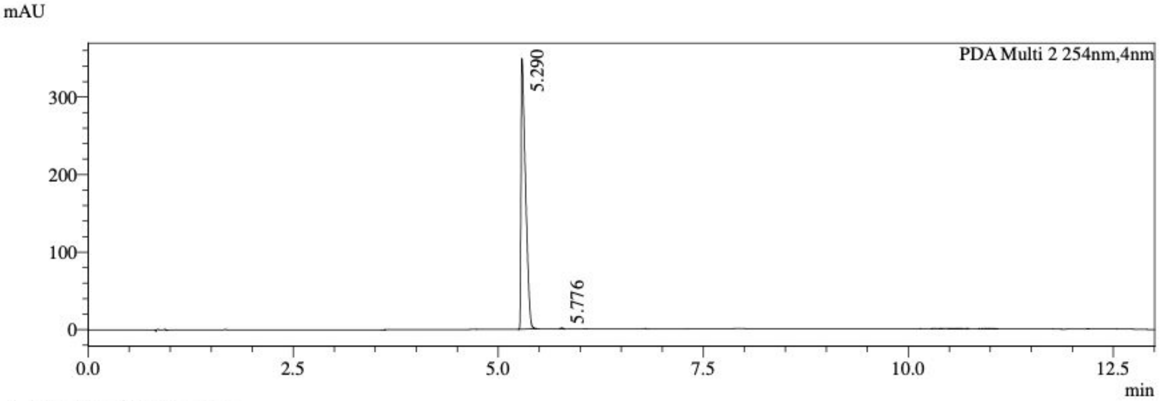


1 PDA Multi 1 / 220nm,4nm
2 PDA Multi 2 / 254nm,4nm

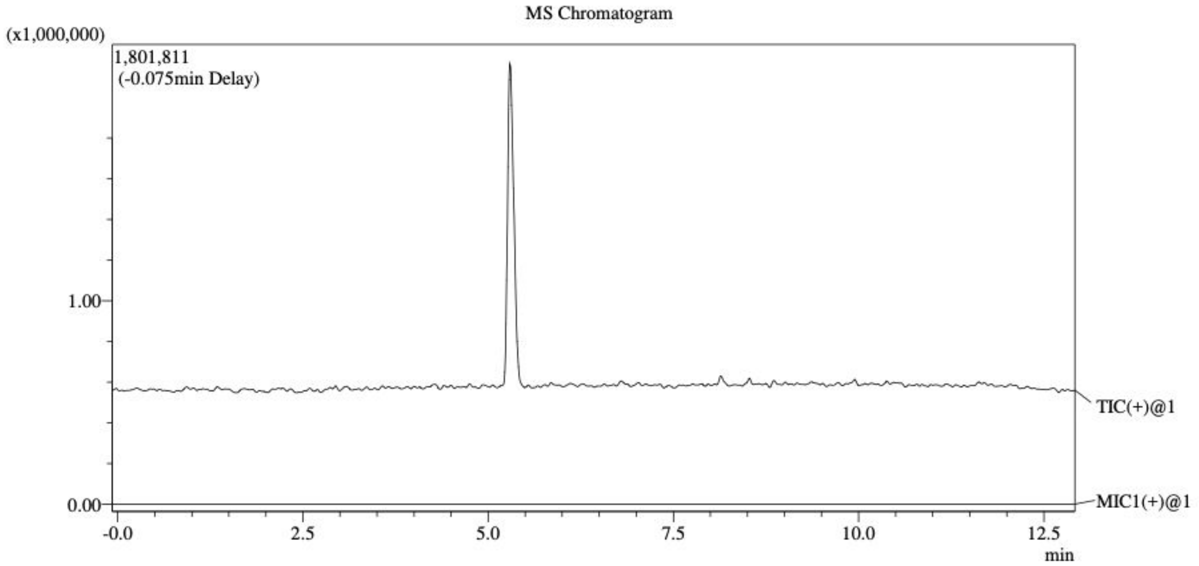


Integration Result

Peak Table

PDA Ch1 220nm

| Peak# | Ret. Time | USP Width | Resolution | Height | Area | Area% |
|---|---|---|---|---|---|---|
| 1 | 5.290 | 0.108 | -- | 1334740 | 5086817 | 99.762 |
| 2 | 5.776 | 0.049 | 6.196 | 6852 | 12125 | 0.238 |
| Total | | | | | 5098942 | 100.000 |

PDA Ch2 254nm

| Peak# | Ret. Time | USP Width | Resolution | Height | Area | Area% |
|---|---|---|---|---|---|---|
| 1 | 5.290 | 0.108 | -- | 349094 | 1322523 | 99.838 |
| 2 | 5.776 | 0.043 | 6.448 | 1403 | 2143 | 0.162 |
| Total | | | | | 1324666 | 100.000 |

MS Spectrum

Spectrum Mode:Averaged 5.283-5.300(644-646) Base Peak:407.1(1264454)Positive(ESI+)
D:\data\2023\2307\230712\ET74780-123-p1p32.lcd

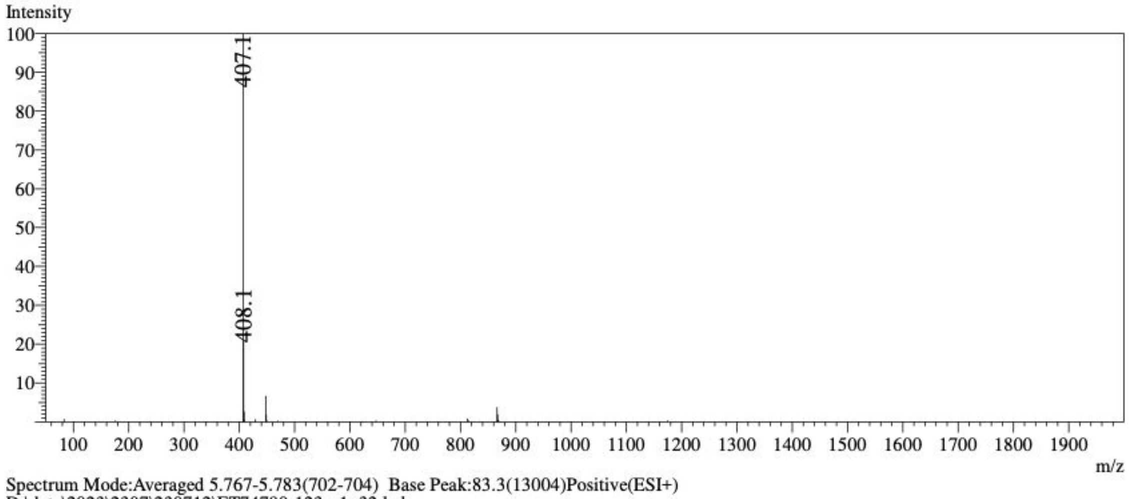


Spectrum Mode:Averaged 5.767-5.783(702-704) Base Peak:83.3(13004)Positive(ESI+)
D:\data\2023\2307\230712\ET74780-123-p1p32.lcd

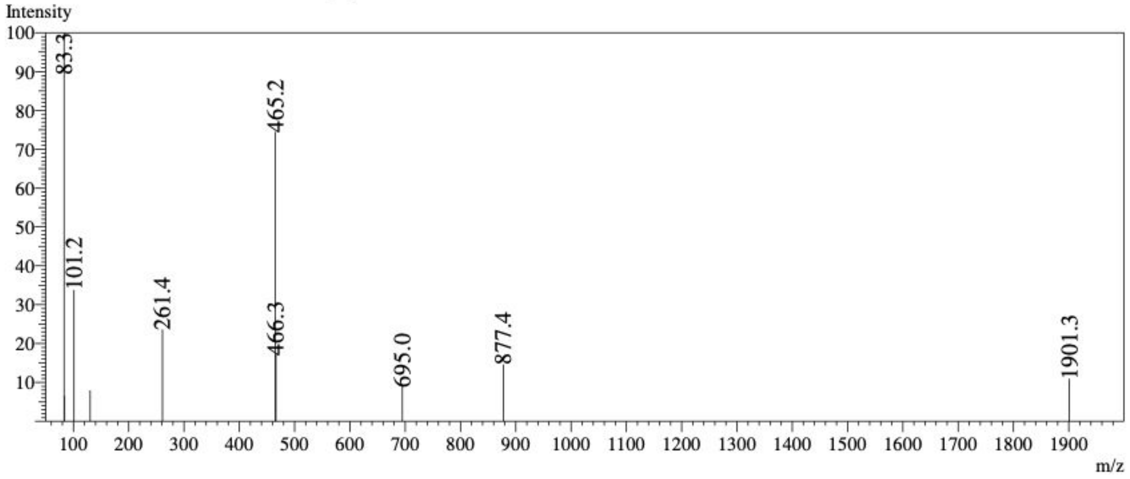


**CHIRAL HPLC -TNG456**

| | | | |
|---|---|---|---|
| Acq Method : | OJ_IPA_05IPAm_10_50_25_35_5_B4 | Processing Method: | 00 |
| Date Acquired: | 7/13/2023 10:41:35 AM CST | Injection Volume: | 1.00 ul |
| Date Processed: | 7/13/2023 11:06:30 AM CST | Vial: | 1:C,1 |
| Channel Name: | 288.0nm | Run Time: | 5.0 Minutes |
| Project Name: | 2023\SFC-M-20230627 | Raw Data: | D:\Data\ID_m32_result_ 34442 |
| User Name: | CASTJ_CA (CASTJ_CA) | Label: | AssayEE |
| Instrument: | CAS-TJ-ANA-SFC-M (Waters UPCC with SQ Detector 2) | | |
| Proc. Chnl. Descr.: | PDA Spectrum PDA 288.0 nm (PDA Spectrum (190-300)nm) | | |

## Test Results

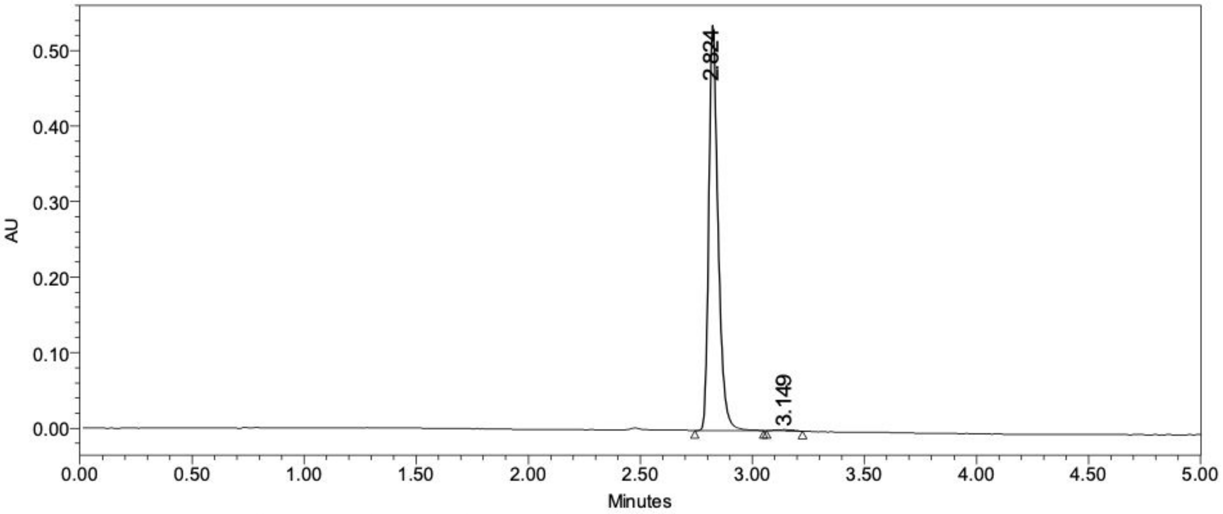


| | RT | Area | % Area | Height |
|---|---|---|---|---|
| 1 | 2.824 | 1551125 | 99.52 | 536026 |
| 2 | 3.149 | 7457 | 0.48 | 1459 |

Stored: 7/10/2023 3:32:14 PM CST

### Method Information

| | |
|---|---|
| Method Comments | "Column:Chiralcel OJ-3,150×4.6mm I.D.,3um<br>Mobile phase:A: $CO_2$ B:IPA(0.5%IPAm, v/v)<br>Gradient:<br>Time A% B%<br>0.0 90 10<br>0.5 90 10<br>3.5 50 50<br>4.5 50 50<br>5.0 90 10<br>Flow rate:2.5mL/min<br>Column temp.:35°C<br>ABPR:2000psi" |
| Method Modified User | CASTJ_CA |
| Method Locked | No |
| Method Id | 27079 |
| Old Id | |
| Method Version | 2 |
| Method Edit User | |
| Source S/W Info | Empower 3 Software Build 3471 SPs Installed: Service Release 3 DB ID: 29266954[illegible] |

**FT-IR SPECTRUM – TNG456**

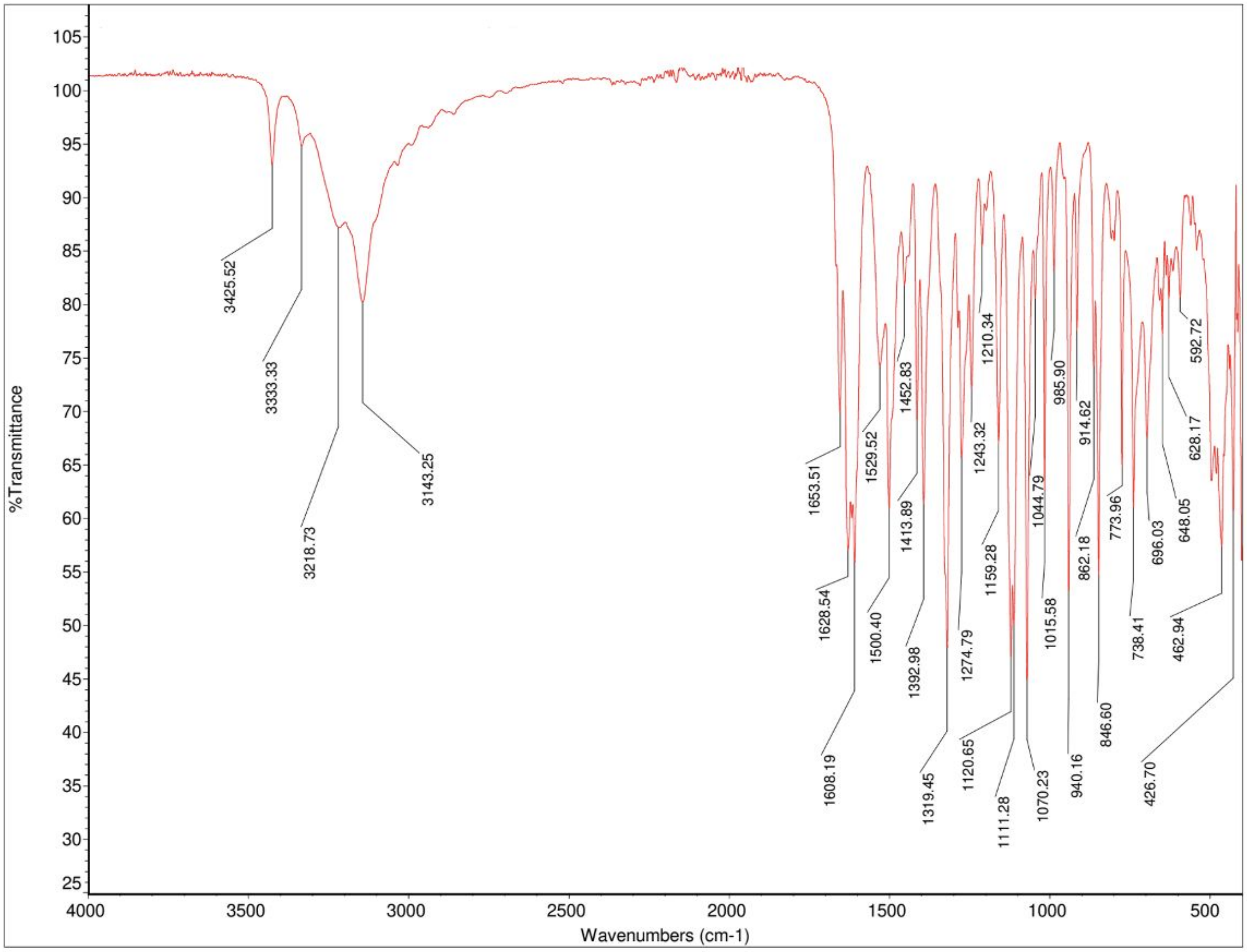


**SMALL MOLECULE CRYSTAL STRUCTURE OF TNG456 (FIGURE SI-1)**

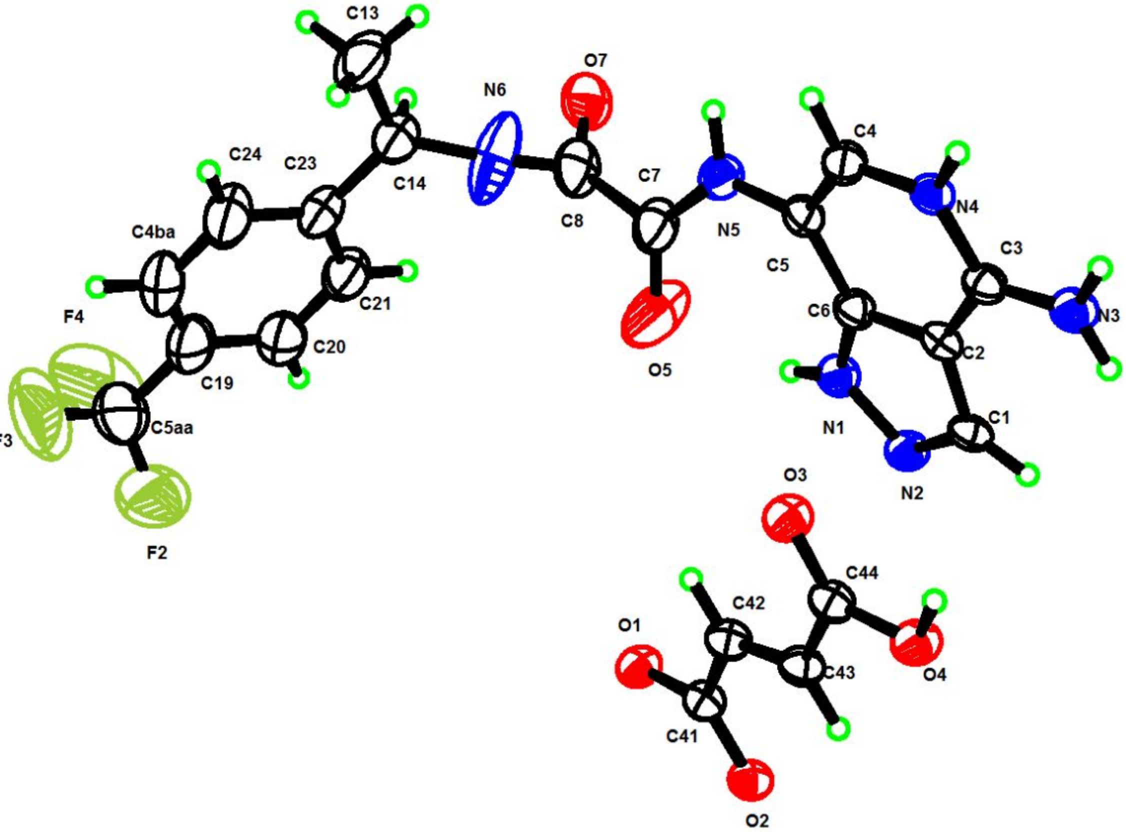


**Figure SI-1. ORTEP image of single crystals of TNG456 fumarate salt obtained from slow evaporation experiment in MeOH.**

## INTRAMOLECULAR HYDROGEN BONDING FOR A MODEL AMINOPYRAZOLOPYRIDINE OXAMIDE

The preferred tautomer and conformations in solution of the model $N^1$-(4-amino-1*H*-pyrazolo[4,3-*c*]pyridin-7-yl)-$N^2$,$N^2$-dimethyloxalamide was predicted on the Orion platform using the QM Tautomer prediction Floe. The other pyrazole tautomer was disfavored by 13.5 kcal/mol using B3LYP-D3/6-31G**.[3] The QM conformer ensemble workflow on the Orion platform was used to create up to 100 conformations that were then optimized using B3LYP-D3/6-31G* method with constrained dihedrals. 16 distinct conformations within the lowest 10 kcal/mol using a single point energy with a triple-zeta basis set were kept and exported. The conformers were then reoptimized with frozen dihedrals using B3LYP/6-311G** using Schrodinger Jaguar and then fully optimized.[4] Frequency calculations were used to confirm geometries were true minima with no imaginary frequencies. CPCM solvation energy calculations using Bondi radii were also done to further assess the preference for intramolecular hydrogen bonds in solution. The preference in solution (CPCM//B3LYP-D3/6-311G**) was decreased to 2.6 kcal/mol.

## PREDICTION OF CONFORMATIONAL RESTRICTION OF *R*-METHYLBENZYL AND *S*-METHYLBENZYL OXAMIDES

The pyridine and oxamide core from the the TNG908 crystal structure (pdb 8VEY) were constrained to match the bound conformation up to the furthest nitrogen, and the torsion of interest shown in Figure SI-2 formed by the C(N-methyl) - N(oxamide) - C(benzylic) - C(phenyl) was varied. The rotation was done in sequential 5 degree increments and optimized using B3LYP/6-31G* with all atoms starting with the nitrogen fixed leaving the phenyl group free to rotate. The relaxed coordinate scan was performed in Schrodinger Jaguar with other default settings.

## DOCKING OF *R*-METHYLBENZYL AND *S*-METHYLBENZYL OXAMIDES

To better understand the potential binding preference for the two possible stereoisomers, they were docked into the previously reported TNG908-bound PRMT5 crystal structure (pdb 8VEY) using Schrodinger Glide.[5,6] The Schrodinger protein preparation wizard was used to add hydrogens and choose protonation states (Glu444 was set to be neutral as predicted by the integrated PROPKA algorithm), and after a restrained optimization, all waters were removed except for the water hydrogen bonding to the oxamide carbonyl and the water interacting with it. The MTA cofactor was kept and the Glide docking grid was calculated using default options centered on the position of the ligand (TNG908). The fixed conformations of the *R*- and *S*-methylbenzyl from the torsional scans were docked rigidly into the receptor using Glide SP. The scan conformations already

incorporated a constrained core to match TNG908. The docked *R*-methyl conformers showed good heavy atom overlap with the original bound ligand and had docking scores between -9.6 and -10.7 for the rotamers around 0.3 kcal/mol relative to the minimum in the gas phase. In contrast, the low relative energy *S*-methyl conformers that docked successfully had significantly worse docking scores (-7 kcal/mol best) and did not align well with the TNG908 piperidine. Completely flexible ligand docking was able to find an *S*-methyl isomer pose with only a slightly worse score (0.4 kcal/mol) from the *R*-methyl isomer but the pose had a high strain energy and did not align as well to the crystal structure ligand. As described in the text, the obtained **TNG456**-bound PRMT5 crystal structure showed an unexpected pose for **TNG456** with a change in protein side chains. Using this **TNG456**-bound structure (pdb 9ZL4) for docking yielded a similar result, however. The *S*-methyl isomer had poses with similar, but always slightly worse, docking scores than the *R*-methyl isomer and a high strain energy (>5 kcal/mol using the Schrodinger Strain Energy Rescoring). The alignment with the native ligand was also not as good. The *R*-methyl isomer pose with the best docking score had a low strain energy (0.0 kcal/mol) and the heavy atoms matched the TNG456 ligand heavy atoms in common with a low RMSD of 0.28 Å. As shown in Figure SI-3, the lowest energy rotamer from Figure SI-2 aligned well with an RMSD between the shared heavy atoms of only 0.46Å.

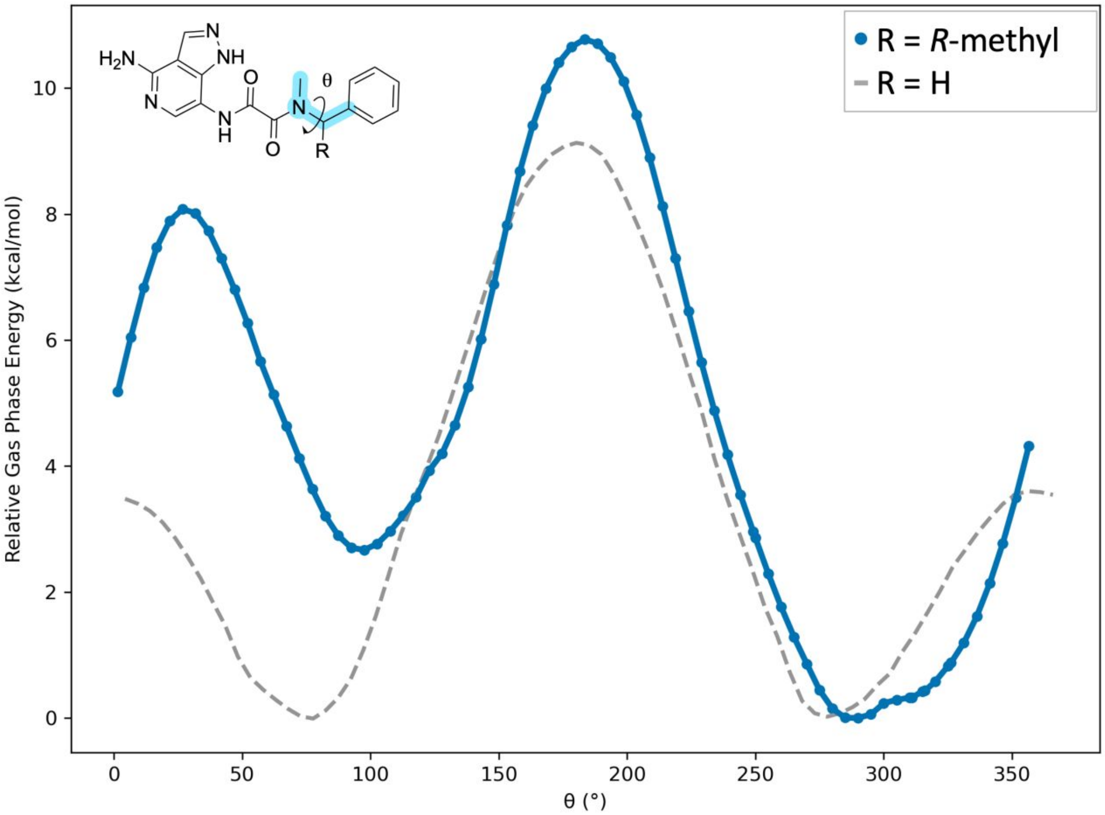


**Figure SI-2. Torsional energy profile for *N*-(methylbenzyl) isomers vs. *N*-benzyl for the angle with a constrained aminopyrazolopyridineoxamide core. By adding a methyl group to the benzyl position, one of the two minima for benzyl becomes strongly disfavored. The *R*-methyl isomer had the best docking score and aligned perfectly with the piperidine-oxamide of TNG908 when docked into the TNG908-bound structure, while the *S*-methyl had a significantly poorer docking score (-7 vs. -11). The atoms of the *S*-methylbenzyl core also did not overlap with the native ligand.**

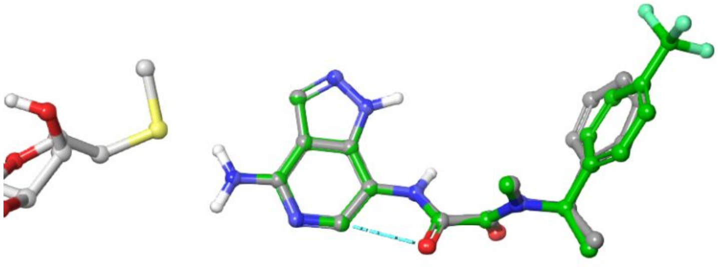

**Figure SI-3. Overlay of lowest energy rotamer from SI-2 (constrained, bound oxamide core) with the bound crystal structure of TNG456.**

### BIOCHEMICAL RECOVERY ASSAY TO MEASURE $K_I$ OF TNG456 IN THE ABSENCE AND PRESENCE OF MTA

The biochemical measurement of PRMT5 enzyme activity recovery and calculation of $K_i$ of **TNG456** in the absence and presence of MTA follow the methods described in supporting information for TNG908[1]. The H4 peptide concentration was 10 µM and MTA concentration was 15 µM.

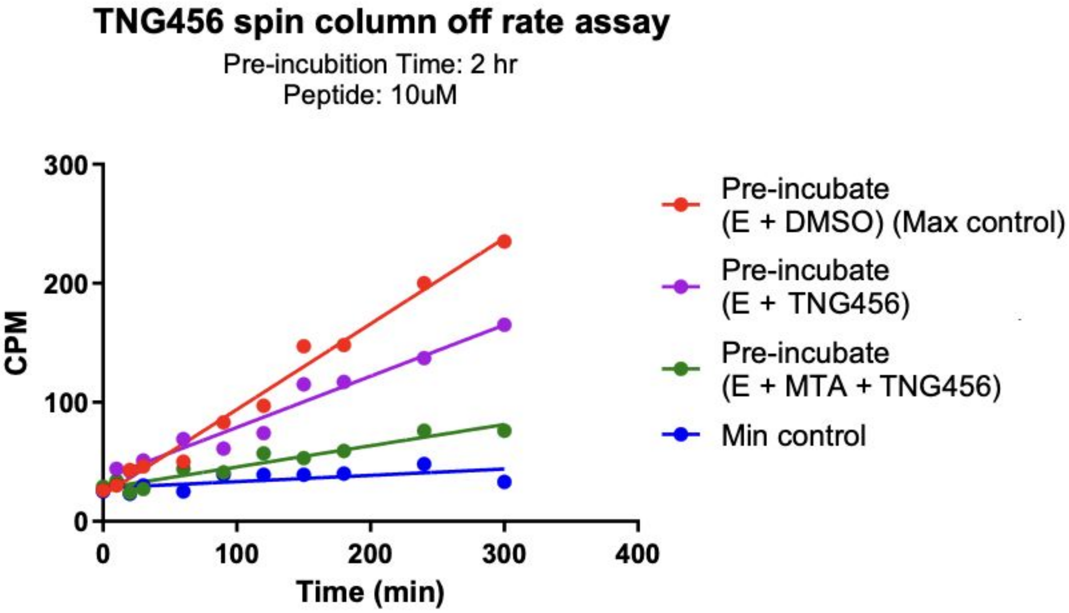


**Figure SI-4. Representative progress curves of PRMT5 enzyme activity recovery from PRMT5·TNG456 binary complex (purple) and PRMT5·MTA·TNG456 ternary complex (green).**

**Table SI-1. Calculated $K_i$ values of TNG456 against PRMT5/MEP50 in the absence and presence of MTA**

| Experiment No. | $K_i$ in the absence of MTA, pM | $K_{i\_}$ in the presence of MTA, pM | Fold Change |
|---|---|---|---|
| N=1 | 24.3 | 2.5 | > 10 |
| N=2 | 36.6 | 2.2 | > 16 |
| Average | 30.4 | 2.3 | >13 |

**METHYLTRANSFERASE PANEL DATA FOR TNG456**

**Table SI-2. TNG462 inhibition profile across 40 methyltransferases**

| Methyltransferase | Substrate | Substrate Concentration | **TNG456**, % Enzyme Activity (@ 10 μM) Data 1 | **TNG456**, % Enzyme Activity (@ 10 μM) Data 2 | Control $IC_{50}$ (M) |
|---|---|---|---|---|---|
| ASH1L | Nucleosomes | 0.05 mg/mL | 93.44 | 98.52 | 2.99E-08 |
| DNMT | Poly dl-dC | 0.001 mg/mL | 102.50 | 94.36 | 1.19E-07 |
| DNMT3a | Lambda DNA | 0.0075 mg/mL | 90.60 | 87.38 | 3.25E-07 |
| DNMT3b | Lambda DNA | 0.0075 mg/mL | 99.04 | 95.25 | 1.02E-07 |
| DNMT3b/3L | Lambda DNA | 0.0075 mg/mL | 92.49 | 93.79 | 4.65E-08 |
| DOT1L | Nucleosomes | 0.05 mg/mL | 93.60 | 91.52 | 1.72E-07 |
| EZH1 Complex | Core Histone | 0.05 mg/mL | 95.84 | 99.58 | 4.40E-05 |
| EZH2 Complex | Core Histone | 0.05 mg/mL | 97.87 | 101.15 | 1.85E-05 |
| EZH2(Y641F) Complex | Core Histone | 0.05 mg/mL | 90.70 | 98.03 | 6.09E-05 |
| G9a | Histone H3 1-21 | 2.5 μM | 81.01 | 87.40 | 1.22E-06 |
| GLP | Histone H3 1-21 | 2.5 μM | 91.04 | 86.45 | 3.45E-07 |
| MLL1 Complex | Nucleosomes | 0.05 mg/mL | 80.12 | 82.27 | 1.64E-07 |
| MLL2 Complex | Nucleosomes | 0.05 mg/mL | 80.24 | 81.91 | 1.74E-05 |
| MLL3 Complex | Nucleosomes | 0.05 mg/mL | 86.89 | 93.48 | 6.74E-06 |
| MLL4 Complex | Nucleosomes | 0.05 mg/mL | 94.13 | 93.22 | 7.54E-07 |
| NSD1 | Nucleosomes | 0.05 mg/mL | 82.86 | 83.45 | 7.87E-06 |
| NSD2 | Nucleosomes | 0.05 mg/mL | 101.21 | 94.08 | 1.37E-06 |
| NSD2 (E1099K) | Nucleosomes | 0.05 mg/mL | 96.60 | 96.04 | 1.45E-06 |
| NSD2 (T1150A) | Nucleosomes | 0.05 mg/mL | 92.34 | 90.19 | 1.35E-06 |
| NSD3 | Nucleosomes | 0.05 mg/mL | 108.55 | 102.98 | 7.63E-08 |
| PRDM9 | Histone H3 | 5 μM | 99.21 | 96.92 | 2.79E-06 |
| PRMT1 | Histone H4 | 5 μM | 97.33 | 94.53 | 3.86E-07 |
| PRMT3 | Histone H4 | 5 μM | 91.48 | 99.01 | 9.03E-07 |
| PRMT4 | Histone H3 | 5 μM | 94.50 | 95.29 | 3.44E-07 |
| PRMT5/MEP50 Complex | Histone H2A | 5 μM | 1.56 | 2.10 | 5.36E-07 |

| | | | | | |
|---|---|---|---|---|---|
| PRMT5 (C449S) /MEP50 Complex | Histone H2A | 5 µM | 1.19 | 1.12 | 1.70E-06 |
| PRMT6 | GST-GAR | 5 µM | 96.30 | 98.66 | 7.70E-08 |
| PRMT7 | GST-GAR | 5 µM | 102.74 | 106.63 | 1.00E-07 |
| PRMT8 | Histone H4 | 5 µM | 100.21 | 98.08 | 2.91E-07 |
| SET1b Complex | Core Histone | 0.05 mg/mL | 98.64 | 96.25 | 4.37E-06 |
| SET7/9 | Core Histone | 0.05 mg/mL | 99.78 | 93.98 | 8.79E-05 |
| SET8 | Nucleosomes | 0.05 mg/mL | 99.31 | 98.31 | 1.03E-07 |
| SETDB1 | Histone H3 | 5 µM | 95.99 | 92.91 | 3.87E-06 |
| SETD2 | Nucleosomes | 0.05 mg/mL | 59.97 | 60.16 | 2.36E-06 |
| SMYD2 | Histone H4 | 5 µM | 107.08 | 102.92 | 1.89E-07 |
| SMYD3 | MEKK2 | 0.5 µM | 97.84 | 90.70 | 3.63E-05 |
| SUV39H1 | Histone H3 | 5 µM | 100.98 | 102.06 | 1.32E-04 |
| SUV39H2 | Histone H3 | 5 µM | 86.01 | 87.58 | 1.80E-05 |
| SUV420H1TV2 | Nucleosomes | 0.05 mg/mL | 94.00 | 95.93 | 1.22E-04 |

PRMT9 = no signficant inhibition at 30µM with 200ng protein in 10µL buffer solution.

### EUROFINS SAFETYSCAN PANEL FOR TNG456

**Table SI-3. TNG456 E/IC50 ELECT data across 78 assays**

| Target Class | Assay Name | Assay Target | Mode | Result Type | RC50 (uM) | Max Response |
|---|---|---|---|---|---|---|
| GPCR | Calcium Flux | ADORA2A | Agonist | EC50 | >30 | 0 |
| GPCR | Calcium Flux | ADRA1A | Agonist | EC50 | >30 | 6.02 |
| GPCR | Calcium Flux | AVPR1A | Agonist | EC50 | >30 | 5.14 |
| GPCR | Calcium Flux | CCKAR | Agonist | EC50 | >30 | 0.39 |
| GPCR | Calcium Flux | CHRM1 | Agonist | EC50 | >30 | 2.03 |
| GPCR | Calcium Flux | CHRM3 | Agonist | EC50 | >30 | 2.26 |
| GPCR | Calcium Flux | EDNRA | Agonist | EC50 | >30 | 4.37 |
| GPCR | Calcium Flux | HRH1 | Agonist | EC50 | >30 | 4.23 |
| GPCR | Calcium Flux | HTR2A | Agonist | EC50 | >30 | 0.29 |
| GPCR | Calcium Flux | HTR2B | Agonist | EC50 | >30 | 0 |
| GPCR | Calcium Flux | ADORA2A | Antagonist | IC50 | >30 | 0 |
| GPCR | Calcium Flux | ADRA1A | Antagonist | IC50 | >30 | 30.86 |
| GPCR | Calcium Flux | AVPR1A | Antagonist | IC50 | >30 | 0.65 |
| GPCR | Calcium Flux | CCKAR | Antagonist | IC50 | >30 | 19.71 |
| GPCR | Calcium Flux | CHRM1 | Antagonist | IC50 | >30 | 1.09 |
| GPCR | Calcium Flux | CHRM3 | Antagonist | IC50 | >30 | 4.58 |

| | | | | | | |
|---|---|---|---|---|---|---|
| GPCR | Calcium Flux | EDNRA | Antagonist | IC50 | >30 | 0 |
| GPCR | Calcium Flux | HRH1 | Antagonist | IC50 | >30 | 1.76 |
| GPCR | Calcium Flux | HTR2A | Antagonist | IC50 | 20.34043 | 54.57 |
| GPCR | Calcium Flux | HTR2B | Antagonist | IC50 | >30 | 25.26 |
| GPCR | cAMP | ADRA2A | Agonist | EC50 | >30 | 0.8 |
| GPCR | cAMP | ADRB1 | Agonist | EC50 | >30 | 0 |
| GPCR | cAMP | ADRB2 | Agonist | EC50 | >30 | 0.55 |
| GPCR | cAMP | CHRM2 | Agonist | EC50 | >30 | 37.82 |
| GPCR | cAMP | CNR1 | Agonist | EC50 | >30 | 0 |
| GPCR | cAMP | CNR2 | Agonist | EC50 | >30 | 0 |
| GPCR | cAMP | DRD1 | Agonist | EC50 | >30 | 1.82 |
| GPCR | cAMP | DRD2S | Agonist | EC50 | >30 | 10.56 |
| GPCR | cAMP | HRH2 | Agonist | EC50 | >30 | 0 |
| GPCR | cAMP | HTR1A | Agonist | EC50 | >30 | 4.32 |
| GPCR | cAMP | HTR1B | Agonist | EC50 | >30 | 16.96 |
| GPCR | cAMP | OPRD1 | Agonist | EC50 | >30 | 24.93 |
| GPCR | cAMP | OPRK1 | Agonist | EC50 | >30 | 13.82 |
| GPCR | cAMP | OPRM1 | Agonist | EC50 | >30 | 17.49 |
| GPCR | cAMP | ADRA2A | Antagonist | IC50 | >30 | 15.33 |
| GPCR | cAMP | ADRB1 | Antagonist | IC50 | >30 | 9.79 |
| GPCR | cAMP | ADRB2 | Antagonist | IC50 | >30 | 14.78 |
| GPCR | cAMP | CHRM2 | Antagonist | IC50 | >30 | 0 |
| GPCR | cAMP | CNR1 | Antagonist | IC50 | >30 | 22.73 |
| GPCR | cAMP | CNR2 | Antagonist | IC50 | >30 | 14.17 |
| GPCR | cAMP | DRD1 | Antagonist | IC50 | >30 | 13.67 |
| GPCR | cAMP | DRD2S | Antagonist | IC50 | >30 | 49.24 |
| GPCR | cAMP | HRH2 | Antagonist | IC50 | >30 | 2.03 |
| GPCR | cAMP | HTR1A | Antagonist | IC50 | >30 | 0 |
| GPCR | cAMP | HTR1B | Antagonist | IC50 | >30 | 4.59 |
| GPCR | cAMP | OPRD1 | Antagonist | IC50 | >30 | 0 |
| GPCR | cAMP | OPRK1 | Antagonist | IC50 | >30 | 20.59 |

| | | | | | | |
|---|---|---|---|---|---|---|
| GPCR | cAMP | OPRM1 | Antagonist | IC50 | >30 | 2.72 |
| Ion Channel | Ion Channel | CAV1.2 | Blocker | IC50 | >30 | 0.51 |
| Ion Channel | Ion Channel | GABAA | Blocker | IC50 | >30 | 8.36 |
| Ion Channel | Ion Channel | hERG | Blocker | IC50 | >30 | 49.58 |
| Ion Channel | Ion Channel | HTR3A | Blocker | IC50 | 19.08271 | 61.03 |
| Ion Channel | Ion Channel | KvLQT1/minK | Blocker | IC50 | >30 | 0 |
| Ion Channel | Ion Channel | nAChR(a4/b2) | Blocker | IC50 | >30 | 22.65 |
| Ion Channel | Ion Channel | NAV1.5 | Blocker | IC50 | 12.10599 | 76.37 |
| Ion Channel | Ion Channel | NMDAR (1A/2B) | Blocker | IC50 | >30 | 18.05 |
| Ion Channel | Ion Channel | GABAA | Opener | EC50 | >30 | 0.08 |
| Ion Channel | Ion Channel | HTR3A | Opener | EC50 | >30 | 14.3 |
| Ion Channel | Ion Channel | KvLQT1/minK | Opener | EC50 | >30 | 0.66 |
| Ion Channel | Ion Channel | nAChR(a4/b2) | Opener | EC50 | >30 | 33.56 |
| Ion Channel | Ion Channel | NMDAR (1A/2B) | Opener | EC50 | >30 | 11.5 |
| Kinases | Binding | INSR | Inhibitor | IC50 | >30 | 6.3 |
| Kinases | Binding | LCK | Inhibitor | IC50 | >30 | 2.31 |
| Kinases | Binding | ROCK1 | Inhibitor | IC50 | >30 | 37.37 |
| Kinases | Binding | VEGFR2 | Inhibitor | IC50 | >30 | 26.14 |
| NHR | NHR Nuclear Translocation | AR | Agonist | EC50 | >30 | 0 |
| NHR | NHR Nuclear Translocation | AR | Antagonist | IC50 | >30 | 0 |
| NHR | NHR Protein Interaction | GR | Agonist | EC50 | >30 | 0 |
| NHR | NHR Protein Interaction | GR | Antagonist | IC50 | >30 | 11.28 |
| Non-Kinase Enzymes | Enzymatic | AChE | Inhibitor | IC50 | >30 | 6.24 |
| Non-Kinase Enzymes | Enzymatic | COX1 | Inhibitor | IC50 | >30 | 10.61 |
| Non-Kinase Enzymes | Enzymatic | COX2 | Inhibitor | IC50 | >30 | 11.67 |
| Non-Kinase Enzymes | Enzymatic | MAOA | Inhibitor | IC50 | >30 | 11.28 |
| Non-Kinase Enzymes | Enzymatic | PDE3A | Inhibitor | IC50 | >30 | 1.87 |
| Non-Kinase Enzymes | Enzymatic | PDE4D2 | Inhibitor | IC50 | >30 | 10.45 |
| Transporter | Transporter | DAT | Blocker | IC50 | >30 | 40.61 |
| Transporter | Transporter | NET | Blocker | IC50 | >30 | 13.03 |
| Transporter | Transporter | SERT | Blocker | IC50 | 20.28289 | 58.22 |

### MDCKII AND MDR1-MDCKII ASSAYS.

Wild type (WT) MDCKII cells or MDR1-MDCKII cells (both obtained from Piet Borst at the Netherlands Cancer Institute) were seeded onto the polycarbonate membranes in the 96- well insert system at 4.44 x $10^5$ cells/mL and cultured for 4-7 days until confluence before being used for the transport studies. Test compounds were diluted with the transport buffer (HPSS with 10 mM HEPES, pH 7.4) from DMSO stock solution to a concentration of 2 µM (DMSO < 1%) and applied to the apical or basolateral side of the cell monolayer. The plate was incubated for 2.5 h in $CO_2$ incubator at 37±1 °C, with 5% $CO_2$ at saturated humidity without shaking. Permeation of the test compounds from A to B or B to A direction was determined in duplicate. In addition, the efflux ratio of each compound was also calculated. For each transport assay, digoxin (P-gp efflux substrate) was tested at 10.0 µM bidirectionally, while nadolol (low permeability marker) and metoprolol (high permeability marker) were tested at

2.00 μM in A to B direction in duplicate. Test and reference compounds were quantified by LC-MS/MS analysis based on the peak area ratio of analyte/internal standard (IS). After transport assay, Lucifer yellow fluorescence rejection assay was performed to confirm the integrity of the cell monolayer.

#### HUMAN LIVER MICROSOME METABOLIC STABILITY ASSAY.

Test and reference compounds (testosterone, diclofenac and propafenone) at 1 μM were incubated individually in human liver microsome (0.5 mg protein/mL, from mixed-gender donors) supplemented with 1 mM NADPH at 37 °C for 60 minutes while shaking. Aliquots of 60 μl were taken at 5, 15, 30, 45 and 60 minutes of incubation and reactions were stopped by adding 180 ul of quenching solution. After which all sampling plates were shaken for 10 minutes, then centrifuged at 4000 rpm for 20 minutes at 4 °C. Supernatants were transferred to HPLC water (1:3) and mixed for 10 minutes prior to the LC-MS/MS analysis. Test and reference compounds were quantified by LC-MS/MS analysis based on the peak area ratio of analyte/internal standard (IS).

#### HEPATOCYTE STABILITY ASSAY METHOD

Cryopreserved hepatocytes were thawed, washed, and resuspended in pre-warmed incubation medium to achieve a final concentration of $0.5\times10^6$ cells/mL. Test compounds and positive controls were prepared as 100 μM dosing solutions in DMSO. In a 96-well plate, 198 μL of hepatocyte suspension was mixed with 2 μL of dosing solution to achieve a final test compound concentration of 1 μM. Plates were incubated at 37 °C in a 5% $CO_2$ atmosphere with constant shaking (600 rpm). At specified time points (0, 15, 30, 60, and 90 minutes), 20 μL aliquots were transferred to wells containing 100 μL of ice-cold stop solution (acetonitrile with internal standards). Samples were mixed, centrifuged at 3,220 × g for 20 minutes at 4 °C, and supernatants were transferred to pre-labeled plates. Sample analysis was conducted using LC-MS/MS, and the percentage of compound remaining was calculated to assess stability.

#### HUMAN ETHER-A-GO-GO-RELATED GENE (HERG) ASSAY.

CHO cells stably expressing hERG potassium channels from Sophion Biosciences were used for this test. The cells were cultured in a humidified and air-controlled (5 % $CO_2$) incubator at 37 °C. The CHO cells which were at least two days after plating and more than 75 % confluent would be used for experiments. Before testing, cells were harvested using TrypLE and resuspended in the physiological solution at the room temperature. For the electrophysiological recordings the following solutions were used (Table SI-4).

**Table SI-4. Composition of Physiological, External, and Internal Solutions**

| Reagent | Physiological Solution (mM) | External Solution (mM) | Internal Solution (mM) |
|---|---|---|---|
| NaCl | 140 | 80 | 10 |
| KCl | 4 | 4 | 10 |
| KF | - | - | 110 |
| $CaCl_2$ | 2 | 2 | - |
| $MgCl_2$ | 1 | 1 | - |
| Glucose | 5 | 5 | - |
| NMDG | - | 60 | - |
| HEPES | 10 | 10 | 10 |
| EGTA | - | - | 10 |
| pH | 7.4 with NaOH | 7.4 with NaOH | 7.4 with KOH |
| Osmolarity | ~298 mOsm | ~289 mOsm | ~280 mOsm |

The physiological solution and external solution were prepared at least one month. The intracellular solution was prepared in batches aliquoted and stored at 4°C until used. Test compounds were dissolved in 100% DMSO to obtain stock solutions for different test concentrations. Then the stock solutions were further diluted into external solution to achieve final concentrations for testing. Visual check for precipitation was conducted before testing. Final DMSO concentration in external solution was not more than 0.30% for the test compounds. Voltage command protocol: From this holding potential of -80 mV, the voltage was first stepped to -50 mV for 80 ms for leak subtraction, and then stepped to +20 mV for 4800 ms to open hERG channels. After that, the voltage was stepped back down to -50 mV for 5000 ms, causing a "rebound" or tail current, which was measured and collected for data analysis. Finally, the voltage was stepped back to the holding potential (-80 mV, 1000 ms). This voltage command protocol was repeated every 20000 msec. This command protocol was performed continuously during the test (vehicle control and test compound). hERG SyncroPatch assay was conducted at room temperature. The Setup, Prime Chip, Catch and Seal Cells, Amplifier Settings, Voltage and Application Protocols were established with Biomek Software (Nanion). One addition of 40 μL of the vehicle was applied, followed by 300s for a baseline period. Then the doses of the compounds were added with 40 μL. The exposure of test compound at each concentration was no less than 300s. The recording for the whole process had to pass the quality control, or the well was abandoned and the compound was retested, all automatically set by PatchControl. Five concentrations (0.30 μM, 1.00 μM, 3.00 μM, 10.00 μM and 30.00 μM) were tested for each compound. A minimum 2 replicates per concentration were obtained. Data analysis was carried out using DataControl, Excel 2013 (Microsoft) and GraphPad Prism 5.0. Within each well recording, percent of control values were calculated for each test compound concentration current response based on peak current in presence of reference control (current response/ peak current) ×100%. The Dose-Response curves were fit to the standard Hill equation as shown below: Ipost cpd/Ipre cpd=Bottom+(Top-Bottom)/(1+10^((Log$IC_{50}$-X)*HillSlope)) Where X is the logarithm of concentration, Ipost cpd/Ipre cpd is the normalized peak current amplitude, Top is 1 and Bottom is equal to 0. Curve-fitting and IC50 calculations were performed

by GraphPad Prism 5.0. If the inhibition obtained at the lowest concentration tested was over 50%, or at the highest concentration tested was less than 50 %, we reported the $IC_{50}$ as less than lowest concentration, or higher than highest concentration, respectively.

## IN VIVO PHARMACOKINETIC STUDIES

The animal studies were conducted in accordance with the testing facilities local IACUC guidelines that are in compliance with the Animal Welfare Act, the Guide for the Care and Use of Laboratory Animals.
The plasma pharmacokinetics of the test compound (TC) were evaluated after dosing male Sprague Dawley rats, beagle dogs, or cynomolgus monkeys. TC was administered either as an intravenous (IV) bolus at 1 mg/kg (in 20% w/v HPβCD and 1% v/v DMSO in saline) or orally (PO) at 3 mg/kg [in 20% w/v HPβCD and 1% v/v DMSO in water for rats, or in 0.5% MC for dogs and monkeys]. Animals were fed prior to IV dosing and fasted prior to PO dosing. Plasma samples were collected from three animals per group at 0.05, 0.25, 0.5, 1, 2, 3, 4, 8, and 24 hours post-dose. TC concentrations were quantified using a qualified liquid chromatography–tandem mass spectrometry (LC-MS/MS) method.

## PLASMA PROTEIN BINDING PROCEDURE

The plasma protein binding of **TNG456** (2 µM) in [cynomolgus monkey, Sprague Dawley rat, or beagle dog] plasma was assessed using an HT-dialysis plate (Model HTD 96b, Cat#1066) and a dialysis membrane (molecular weight cutoff 12–14 kDa, Cat#1101), both purchased from HT Dialysis LLC (Gales Ferry, CT). Samples were incubated at 37 °C for 4 h. **TNG456** concentrations in the donor and receiver chambers were quantified using a fit-for-purpose LC-MS/MS method, and the unbound fraction was calculated as the ratio of receiver to donor chamber concentrations.

## HAP1 MTAP WT AND MTAP-NULL IN-CELL WESTERN ASSAY

Detailed methods can be found in Cottrell et al., 2024.[2] In brief, the HAP1 *MTAP*-isogenic cell line pair was acquired from Horizon Discovery (HZGHC004894c005) and maintained in DMEM (high glucose) + 10 % FBS in a humidified, 10% $CO_2$ tissue culture incubator. The SAM-cooperative PRMT5 inhibitor, GSK3326595, was sourced from Selleck Chemicals and maintained as a 10 mM DMSO stock. HAP1 MTAP WT and MTAP-null cells were treated with compounds for 24 h in 384-well microtiter plates, and then normalized SDMA levels were determined using a multi-mAb SDMA antibody (Cell Signaling 13222) and DRAQ5 (LiCor 926-32211 and VWR 10761-508). Background signal was determined by signal from wells treated with 1 µM GSK3326595. Data analysis was performed using the 4-parameter logistic (4-PL) Hill equation with maximal effect constrained to 0. The fit was performed using GraphPad Prism or in Dotmatics Studies 5.4 as part of a customized data analysis protocol.

## CELL LINE VIABILITY ASSAYS

Detailed methods can be found in Cottrell et al., 2024.[2] In brief, the HAP1 and HCT116 *MTAP*-isogenic cell line pairs were acquired from Horizon Discovery (HZGHC004894c005 and HD R02-033, respectively), the LU99 and LN18 MTAP-isogenic cell line pairs were engineered by stable introduction of a full-length MTAP cDNA under the control of a UbiC promoter. All cell lines were maintained in DMEM (high glucose) + 10% FBS in a humidified, 10% $CO_2$ tissue culture incubator and confirmed for their MTAP status by immunoblot. Cell viability was determined by CellTiter-Glo following 7-days of compound treatment. Data are plotted as % of the DMSO control wells and fit using a 4-parameter logistic (4-PL) Hill equation with maximal effect or baseline constrained to 0. The fit was performed using GraphPad Prism or the default $IC_{50}$ fitting procedure in Dotmatics Studies 5.4 as part of a customized data analysis protocol. Absolute $IC_{50}$s are reported for each cell line.

For the 143-cancer cell line panel potency is reported as a relative IC50 as determined by a 4-parameter logistic (4-PL) Hill equation (GraphPad Prism) and selectivity was visualized by plotting the maximum effect (Amax) of **TNG456** at 2200 nM according to the curve fit.

## IN VIVO PHARMACOLOGY

All protocols for in vivo pharmacology studies were approved by the relevant Institutional Animal Care and Use Committees (Pharmaron, Beijing, China; CrownBio, San Diego, CA, and Taicang and Beijing, China; Champions Oncology, Rockville, MD; and XenoSTART, San Antonio, TX; following the guidance of the Association of Assessment and Accreditation of Laboratory Animal Care.

Following acclimatization, cancer cells were injected subcutaneously into the right flank of 6- to 8-week-old female BALB/c nude mice and allowed to form palpable tumors. Mice were randomized to treatment groups with a mean tumor volume of approximately 160 $mm^3$ (U87MG efficacy), 325 $mm^3$ (U87MG PK/PD) or 205 $mm^3$ (AM38 combination efficacy) in size. **TNG456** was formulated in 5% DMA/20% Captisol. Abemaciclib was formulated in 1% hydroxyethyl cellulose + 0.1% antifoam in 25 mM PB pH 2. PDX studies were conducted with similar study designs. The glioblastoma PDX models used in Figure 12 and Figure 13 are two different models. Tumor volumes were measured using calipers and calculated as (length x width x width) / 2. For data analysis, tumor growth inhibition % TGI = [1-(Treated $TV_{final}$-Treated $TV_{initial}$)/(Vehicle $TV_{final}$-Vehicle $TV_{initial}$)] × 100; tumor regression % TV= [mean $TV_{final}$ – mean $TV_{initial}$] × 100. Tumor volume data were analyzed using GraphPad Prism software.

## WESTERN BLOTTING

Protein lysates were generated by lysis of frozen tumor tissue using RIPA buffer. Samples were normalized by protein concentration using Pierce Rapid Gold BCA Protein Assay Kit (A53225). SDS-PAGE was run using Invitrogen NuPAGE 4-12% Bis-Tris Midi Protein Gels (WG1402BOX). Antibodies SDMA (CST#13222), ACTB (CST#3700) were used at 1:1000 dilution.

## PRMT5:MEP50 EXPRESSION AND PURIFICATION.

Recombinant PRMT5:MEP50 protein for use in biochemical assays and crystallography work was expressed in insect cells and purified as described previously.[2,7] Purified protein was stored as frozen aliquots at -80 °C until further use.

## PRMT5:MEP50 CRYSTALLOGRAPHY.

Crystal structures of MTA-cooperative inhibitors bound to PRMT5 were obtained by first crystallizing the PRMT5:MEP50 + MTA complex as described previously[2] and then soaking crystals with 1 mM compound at room temperature for between 2 - 5 h. The crystals were flash-cooled prior to data collection using a cryo solution that consisted of mother liquor supplemented with 30 % ethylene glycol. Diffraction data were collected at synchrotron sources as noted in Table SI-5. Data reduction and scaling were performed with XDS and AIMLESS[8,9] and structures were refined using REFMAC from the CCP4 software suite and PHENIX.[10,11] Model building was done using COOT[12]. Final structural models

exhibited good geometry, and all final coordinates and structure factors have been deposited in the PDB with accession codes as noted in Table SI-5.

**Table SI-5. X-ray data collection and refinement statistics for PRMT5-MEP50 crystal structures.**

| Compound | Compound 8 | Compound 18 | **TNG456** |
|---|---|---|---|
| Cofactor | MTA | MTA | MTA |
| PDB ID | 9ZL2 | 9ZL3 | 9ZL4 |
| Data Collection | | | |
| Synchrotron source | CLS | SSRF | Spring-8 |
| Beamline | 08ID-1 | BL10U2 | BL45XU |
| Space group | I222 | I222 | I222 |
| Cell dimensions | | | |
| *a, b, c* (Å) | 101.4,136.8,178.1 | 102.4,138.1,178.3 | 100.5,138.3,178.3 |
| Resolution (Å) | 45.9 – 3.18 | 109 – 1.71 | 45.7 – 1.95 |
| Unique reflections | 21184 (3779) | 67184 (3360) | 65491 (3275) |
| Redundancy | 6.7 (6.9) | 8.3 (7.4) | 12.9 (7.2) |
| Completeness (%)† | 99.8 (99.7) | 94.3 (69.9) | 96.2 (83.9) |
| $R_{merge}$† | 0.101 (0.690) | 0.070 (1.20) | 0.093 (0.777) |
| I/σ(I)† | 12.2 (2.4) | 17.9 (1.9) | 18.4 (2.3) |
| $CC_{1/2}$ | 0.999 (0.970) | 0.999 (0.669) | 0.999 (0.840) |
| Refinement | | | |
| Reflections used | 21026 | 67151 | 65482 |
| $R_{work}/R_{free}$ | 0.260 / 0.293 | 0.189 / 0.239 | 0.176 / 0.236 |
| Avg B-value (Å$^2$) | 107 | 39.6 | 33.9 |
| Number of atoms | | | |
| Protein | 7347 | 7388 | 7435 |
| Cofactor /Inhibitor | 49 | 50 | 78 |
| Solvent/Other | 24 | 684 | 875 |
| R.m.s. deviations | | | |
| Bond lengths (Å) | 0.004 | 0.005 | 0.007 |
| Bond angles (º) | 0.875 | 0.720 | 0.872 |

† Values in parentheses are for the highest resolution shell.

‡ Completeness is lower due to anisotropic diffraction correction.

## REFERENCES

(1) Cottrell, K. M.; Briggs, K. J.; Tsai, A.; Tonini, M. R.; Whittington, D. A.; Gong, S.; Liang, C.; McCarren, P.; Zhang, M.; Zhang, W.; Huang, A.; Maxwell, J. P. Discovery of TNG462: A Highly Potent and Selective MTA-Cooperative PRMT5 Inhibitor to Target Cancers with MTAP Deletion. *J. Med. Chem.* **2025**, *68* (5), 5097–5119. https://doi.org/10.1021/acs.jmedchem.4c03067.

(2) Cottrell, K. M.; Briggs, K. J.; Whittington, D. A.; Jahic, H.; Ali, J. A.; Davis, C. B.; Gong, S.; Gotur, D.; Gu, L.; McCarren, P.; Tonini, M. R.; Tsai, A.; Wilker, E. W.; Yuan, H.; Zhang, M.; Zhang, W.; Huang, A.; Maxwell, J. P. Discovery of TNG908: A Selective, Brain Penetrant, MTA-Cooperative

PRMT5 Inhibitor That Is Synthetically Lethal with MTAP-Deleted Cancers. *J. Med. Chem.* **2024**, *67* (8), 6064–6080. https://doi.org/10.1021/acs.jmedchem.4c00133.

(3) Smith, D. G. A.; Burns, L. A.; Simmonett, A. C.; Parrish, R. M.; Schieber, M. C.; Galvelis, R.; Kraus, P.; Kruse, H.; Remigio, R. D.; Alenaizan, A.; James, A. M.; Lehtola, S.; Misiewicz, J. P.; Scheurer, M.; Shaw, R. A.; Schriber, J. B.; Xie, Y.; Glick, Z. L.; Sirianni, D. A.; O'Brien, J. S.; Waldrop, J. M.; Kumar, A.; Hohenstein, E. G.; Pritchard, B. P.; Brooks, B. R.; Schaefer, H. F.; Sokolov, A. Yu.; Patkowski, K.; DePrince, A. E.; Bozkaya, U.; King, R. A.; Evangelista, F. A.; Turney, J. M.; Crawford, T. D.; Sherrill, C. D. PSI4 1.4: Open-Source Software for High-Throughput Quantum Chemistry. *J. Chem. Phys.* **2020**, *152* (18), 184108. https://doi.org/10.1063/5.0006002.

(4) Bochevarov, A. D.; Harder, E.; Hughes, T. F.; Greenwood, J. R.; Braden, D. A.; Philipp, D. M.; Rinaldo, D.; Halls, M. D.; Zhang, J.; Friesner, R. A. Jaguar: A High-performance Quantum Chemistry Software Program with Strengths in Life and Materials Sciences. *Int. J. Quantum Chem.* **2013**, *113* (18), 2110–2142. https://doi.org/10.1002/qua.24481.

(5) Friesner, R. A.; Banks, J. L.; Murphy, R. B.; Halgren, T. A.; Klicic, J. J.; Mainz, D. T.; Repasky, M. P.; Knoll, E. H.; Shelley, M.; Perry, J. K.; Shaw, D. E.; Francis, P.; Shenkin, P. S. Glide: A New Approach for Rapid, Accurate Docking and Scoring. 1. Method and Assessment of Docking Accuracy. *J Med Chem* **2004**, *47* (7), 1739–1749. https://doi.org/10.1021/jm0306430.

(6) Halgren, T. A.; Murphy, R. B.; Friesner, R. A.; Beard, H. S.; Frye, L. L.; Pollard, W. T.; Banks, J. L. Glide: A New Approach for Rapid, Accurate Docking and Scoring. 2. Enrichment Factors in Database Screening. *J. Med. Chem.* **2004**, *47* (7), 1750–1759. https://doi.org/10.1021/jm030644s.

(7) Chan-Penebre, E.; Kuplast, K. G.; Majer, C. R.; Boriack-Sjodin, P. A.; Wigle, T. J.; Johnston, L. D.; Rioux, N.; Munchhof, M. J.; Jin, L.; Jacques, S. L.; West, K. A.; Lingaraj, T.; Stickland, K.; Ribich, S. A.; Raimondi, A.; Scott, M. P.; Waters, N. J.; Pollock, R. M.; Smith, J. J.; Barbash, O.; Pappalardi, M.; Ho, T. F.; Nurse, K.; Oza, K. P.; Gallagher, K. T.; Kruger, R.; Moyer, M. P.; Copeland, R. A.; Chesworth, R.; Duncan, K. W. A Selective Inhibitor of PRMT5 with in Vivo and in Vitro Potency in MCL Models. *Nat. Chem. Biol.* **2015**, *11* (6), 432–437. https://doi.org/10.1038/nchembio.1810.

(8) Kabsch. XDS. *Acta Crystallographica* **2010**, No. d66, 125–132. https://doi.org/10.1107/s0907444909047337.

(9) Evans, P. R.; Murshudov, G. N. How Good Are My Data and What Is the Resolution? *Acta Crystallogr. Sect. D: Biol. Crystallogr.* **2013**, *69* (7), 1204–1214. https://doi.org/10.1107/s0907444913000061.

(10) Murshudov, G. N.; Vagin, A. A.; Dodson, E. J. Refinement of Macromolecular Structures by the Maximum-Likelihood Method. *Acta Crystallogr. Sect. D: Biol. Crystallogr.* **1997**, *53* (3), 240–255. https://doi.org/10.1107/s0907444996012255.

(11) Liebschner, D.; Afonine, P. V.; Baker, M. L.; Bunkóczi, G.; Chen, V. B.; Croll, T. I.; Hintze, B.; Hung, L.-W.; Jain, S.; McCoy, A. J.; Moriarty, N. W.; Oeffner, R. D.; Poon, B. K.; Prisant, M. G.; Read, R. J.; Richardson, J. S.; Richardson, D. C.; Sammito, M. D.; Sobolev, O. V.; Stockwell, D. H.; Terwilliger, T. C.; Urzhumtsev, A. G.; Videau, L. L.; Williams, C. J.; Adams, P. D. Macromolecular Structure Determination Using X-Rays, Neutrons and Electrons: Recent Developments in Phenix. *Acta Crystallogr. Sect. D* **2019**, *75* (Pt 10), 861–877. https://doi.org/10.1107/s2059798319011471.

(12) Emsley, P.; Cowtan, K. Coot: Model-Building Tools for Molecular Graphics. *Acta Crystallogr. Sect. D: Biol. Crystallogr.* **2004**, *60* (12), 2126–2132. https://doi.org/10.1107/s0907444904019158.

## AUTHOR INFORMATION

### Corresponding Author

* **Kevin M. Cottrell** – *Tango Therapeutics, Boston, MA 02215, United States;* Phone: (+1) 857-320-4900; Email: kcottrell@tangotx.com

### Authors

**Kimberly J. Briggs** – *Tango Therapeutics, Boston, MA 02215, United States*
**Alice Tsai** – *Tango Therapeutics, Boston, MA 02215, United States*
**Colin Liang** – *Tango Therapeutics, Boston, MA 02215, United States*
**Patrick McCarren** – *Tango Therapeutics, Boston, MA 02215, United States*
**Douglas A. Whittington** – Tango Therapeutics, Boston, MA 02215, United States
**Minjie Zhang** – *Tango Therapeutics, Boston, MA 02215, United States*
**Wenhai Zhang** – *Tango Therapeutics, Boston, MA 02215, United States*
**Alan Huang** – *Tango Therapeutics, Boston, MA 02215, United States*
**Jannik Andersen** – *Tango Therapeutics, Boston, MA 02215, United States*
**John P. Maxwell** – *Tango Therapeutics, Boston, MA 02215, United States*

## AUTHOR CONTRIBUTIONS

All authors have given approval to the final version of the manuscript.

## ACKNOWLEDGMENTS

We thank the following teams/people for their valuable contributions to this work: Oleg Michurin, Tanya Galushka and colleagues (Enamine, Kyiv, Ukraine). Chen Wei, Wan Shuangyi and colleagues (IDSU, WuXi AppTec, China). Shang Deju and colleagues (CSU, WuXi AppTec, Tianjin, China). Huazhen Wang and colleagues (Viva Biotech, Ltd., Shanghai, China) for X-ray crystallography work. Gang Chen, Xiaoyu Zhu, Kang Yan, and colleagues (WuXi AppTec, Shanghai, China) for peptide displacement and cellular assays. Yuzhou Xu and colleagues (ChemPartner, Shanghai, China) for biochemical characterization studies of **TNG456**. Yingying Ma, Tan Pang and colleagues (Pharmaron Inc, Beijing, China), Johnnie Flores and colleagues (XenoSTART, Texas), Andrew Layne and colleagues (Champions Oncology, Maryland), Jenny Wu and colleagues (Crown Bioscience, Taicang and Beijing, China PK) for in vivo pharmacology studies. Ying Zhou and colleagues (WuXi AppTec, Shanghai, China) for in vitro ADME and studies. Yuxing Chen (ChemPartner, Shanghai, China) and Xiling Wang and colleagues (Biometas, Shanghai, China) for additional cellular assays. Scott Throner for assistance in manuscript editing. All research described in this manuscript was funded by Tango Therapeutics.

## ABBREVIATIONS

ADME, absorption, distribution, metabolism, and excretion; BBB, blood-brain barrier; BID, *bis in die*; CL, clearance; CNS, central nervous system; CPCM, conductor-like polarizable continuum model; CSF, cerebrospinal fluid; DDI, drug-drug interaction; DFT, density functional theory; DMPK, drug metabolism and pharmacokinetics; GBM, glioblastoma multiforme; $GI_{50}$, growth inhibition 50%; GLP, good laboratory practice; HBD, hydrogen bond donor; hERG, human ether-a-go-go related gene; IMHB, intramolecular hydrogen bond; MDCKII, Madin-Darby Canine Kidney-II cells; MDR1, multidrug resistant 1; MTA, 5'-methylthioadenosine; MTAP, methylthioadenosine phosphorylase; PDX, patient-derived xenograft; PRMT5, protein arginine methyltransferase 5; QD, *quaque die*; SAM, *S*-adenosylmethionine; SDMA, symmetric dimethylarginine; SPR, surface plasmon resonance; TGI, tumor growth inhibition; TPSA, topological polar surface area.
